# Identification of Bacteriophage-Encoded Anti-sRNAs in Pathogenic *Escherichia coli*

**DOI:** 10.1016/j.molcel.2014.05.006

**Published:** 2014-07-17

**Authors:** Jai J. Tree, Sander Granneman, Sean P. McAteer, David Tollervey, David L. Gally

**Affiliations:** 1 Wellcome Trust Centre for Cell Biology, The University of Edinburgh, Edinburgh EH9 3JR, UK; 2 The Roslin Institute and Royal (Dick) School of Veterinary Studies, University of Edinburgh, Edinburgh EH25 9RG, UK; 3 Centre for Synthetic and Systems Biology (SynthSys), University of Edinburgh, Edinburgh EH9 3JD, UK

## Abstract

In bacteria, Hfq is a core RNA chaperone that catalyzes the interaction of mRNAs with regulatory small RNAs (sRNAs). To determine in vivo RNA sequence requirements for Hfq interactions, and to study riboregulation in a bacterial pathogen, Hfq was UV crosslinked to RNAs in enterohemorrhagic *Escherichia coli* (EHEC). Hfq bound repeated trinucleotide motifs of A-R-N (A-A/G-any nucleotide) often associated with the Shine-Dalgarno translation initiation sequence in mRNAs. These motifs overlapped or were adjacent to the mRNA sequences bound by sRNAs. In consequence, sRNA-mRNA duplex formation will displace Hfq, promoting recycling. Fifty-five sRNAs were identified within bacteriophage-derived regions of the EHEC genome, including some of the most abundant Hfq-interacting sRNAs. One of these (AgvB) antagonized the function of the core genome regulatory sRNA, GcvB, by mimicking its mRNA substrate sequence. This bacteriophage-encoded “anti-sRNA” provided EHEC with a growth advantage specifically in bovine rectal mucus recovered from its primary colonization site in cattle.

## Highlights

• Transcriptome-wide map of Hfq binding reveals targeting rules
• Many sRNAs identified in pathogenicity islands
• Pathogenicity-associated anti-sRNAs antagonize sRNAs encoded in core genome
• Anti-sRNAs alter cell metabolism as part of niche colonization

## Graphical Abstract

**Figure:**
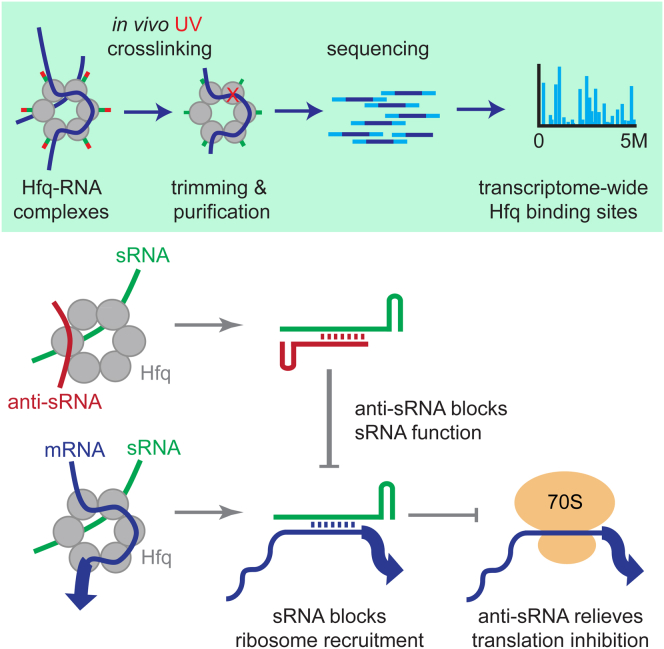

## Teaser

Hfq catalyzes the interaction of small RNAs (sRNA) with mRNAs. Tree et al. use UV-induced RNA-protein crosslinking to define Hfq substrates in pathogenic *Escherichia coli*, identifying abundant sRNAs derived from integrated bacteriophages, including “anti-sRNAs” that inhibit the activity of sRNAs.

## Introduction

RNA-based regulation (riboregulation) plays a pivotal role in modulating transcript stability and translation efficiency in all domains of life. In bacteria, small regulatory RNAs (sRNAs) have emerged as a major class of regulators of mRNA translation and stability. The canonical pathway for repression of mRNA translation involves an sRNA annealing at or close to the Shine-Dalgarno (SD) ribosome binding site to prevent recognition of the transcript by the 30S ribosomal subunit (Bouvier et al., 2008). sRNA-mRNA duplex formation may be coupled to recruitment of RNase E and lead to accelerated turnover of the transcript (Lalaouna et al., 2013; Pfeiffer et al., 2009). However, a broad range of additional sRNA regulatory mechanisms are being uncovered (Bossi et al., 2012).

sRNA regulation in bacteria is best understood in *Escherichia coli* and *Salmonella* Typhimurium, in which select sRNA-mRNA interactions have been intensely studied. The majority of sRNA-mRNA interactions in these bacteria are mediated by Hfq, a pleiotrophic regulator required for posttranscriptional control of bacterial stress responses and for virulence in a range of pathogens (Chao and Vogel, 2010; Papenfort and Vogel, 2010).

Knowledge of how Hfq recognizes RNA targets has largely been derived from in vitro studies using purified Hfq and RNA. Homo-hexamers of Hfq form doughnut-shaped ring structures, with faces defined as “distal” and “proximal.” Cocrystallization of Hfq and poly(A) or poly(U) substrates indicated that the distal face can accommodate a repeated trinucleotide motif composed of A-R(A/G)-N(any nucleotide) (Link et al., 2009), and the proximal face binds hexauridine substrates with a preference for interactions with the 3′OH of poly(U) motifs, such as those found in Rho-independent terminators (Otaka et al., 2011; Sauer and Weichenrieder, 2011). A third RNA-binding site, located on the rim of the Hfq hexamer (“lateral” face) is thought to accommodate the body of the sRNA (Ishikawa et al., 2012; Sauer et al., 2012; Zhang et al., 2013). Conserved arginines at the rim are essential for the chaperone activity of Hfq and have been proposed to nucleate helix formation between complementary mRNA-sRNA pairs (Panja et al., 2013). Global analysis of Hfq binding has been carried out in *Salmonella*, greatly expanding our knowledge of target transcripts and sRNAs in this pathogen (Chao et al., 2012; Sittka et al., 2008).

The enteric pathogen enterohemorrhagic *E*. *coli* (EHEC) has a mosaic genome structure generated by horizontal gene transfer (HGT) into a core genome that is largely conserved in the related but nonpathogenic *E*. *coli* K12 str. MG1655 (Hayashi et al., 2001). Pathogen-specific virulence factors can be encoded within this acquired DNA, which has led to the concept of “pathogenicity islands.” These can be transferred between bacteria following infection with bacteriophages. In addition, lysogenic bacteriophages integrate their prophage genome into that of the recipient bacterium. Over time, these can become cryptic (i.e., unable to produce viable new bacteriophages) due to sequence mutation and loss. EHEC encodes two major virulence factors, both expressed from horizontally acquired regions: Shiga toxins that are responsible for potentially fatal capillary damage within the kidneys and brain (hemolytic uremic syndrome [HUS]) (Tarr et al., 2005) and a type 3 secretion system (T3SS) that is required for colonization of the reservoir host, cattle (Naylor et al., 2005). Many effector proteins injected into host cells by the T3SS are expressed from cryptic bacteriophage genomes, providing one reason for retention of these regions as part of the EHEC genome.

Here the technique of UV-induced RNA-protein *cr*osslinking and *a*nalysis of *c*DNA by high throughput sequencing (CRAC) was applied to identify transcriptome-wide targets of Hfq binding in EHEC O157:H7.

## Results

### UV-Crosslinking of Hfq to Target RNAs In Vivo

The chromosomal copy of Hfq was modified by the addition of dual affinity tags in two *E*. *coli* strains (K12 and EHEC O157) (see Supplementary Information available online). To confirm the functionality of the tagged-Hfq (Hfq-HTF), translational repression of OmpF was measured, since this is known to be Hfq-dependent via targeting of the sRNA MicF (Corcoran et al., 2012). While Hfq-HTF demonstrated mildly reduced activity compared to wild-type Hfq, MicF still repressed OmpF translation by 75%, demonstrating that Hfq-HTF is functional and mediates riboregulation (Figure S1).

**Figure 1:**
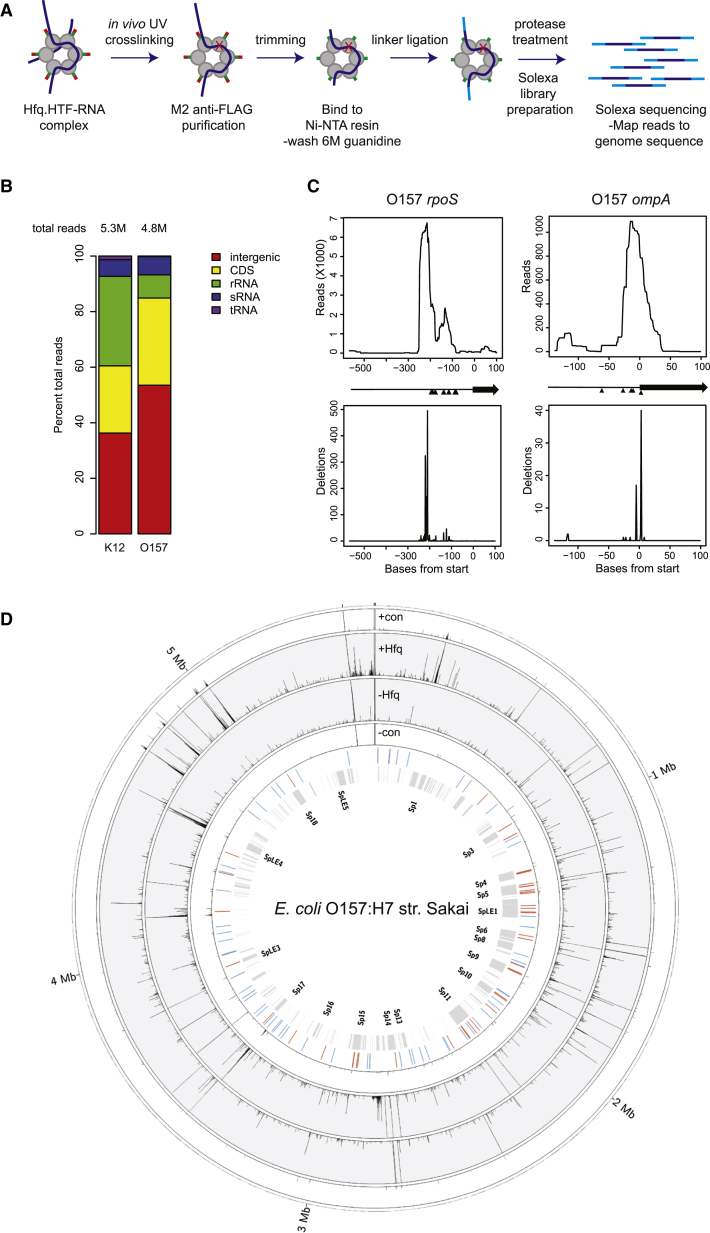
UV Crosslinking of Hfq-RNA Correlates with In Vitro Footprinting of Hfq to Abundant mRNAs (A) Workflow for CRAC analysis of Hfq. A detailed protocol is presented in Supplemental Experimental Procedures and Figure S1. (B) Distribution of Hfq-bound reads between transcript classes in *E*. *coli* K12 str. MG1655 and *E*. *coli* O157 str. Sakai. Total reads are indicated above bars. (C) Sequencing reads recovered from Hfq CRAC that map to *rpoS* or *ompA* mRNAs (top) and deletions recovered within sequencing reads (below). Black arrows between plots indicate the position of coding sequence (arrow) and 5′ UTR (line). Black triangles indicate position of nucleotides protected by Hfq in footprinting experiments in vitro (Moll et al., 2003; Soper and Woodson, 2008). (D) Transcriptome-wide profiling of Hfq binding sites. Numbers of Hfq-associated reads mapped to the positive strand (+Hfq) and negative strand (−Hfq) are plotted in the gray line plots (y axis maximum 20,000 reads). Control experiments with untagged protein are plotted in the white outer and inner line plots (con±; y axis maximum 10,000 reads). From the inner-most track: text indicates designations for pathogenicity islands, with the position of all pathogenicity islands indicated by the gray boxes in the next track. The positions of sRNAs identified in this study are indicated in red, with previously described sRNAs in blue.

The HTF tag allowed highly stringent purification of Hfq from both strains (Figure 1A; Supplemental Information). To assess the crosslinking efficiency, RNA bound to purified, denatured Hfq was 5′ end labeled with ^32^P (Figures S1A and S1C). Following protease digestion, the recovered RNA was identified by RT-PCR amplification (Figure S1D) and sequencing. Crosslinking was performed independently five times in O157 and twice in K12. Proportions of functional classes of RNA recovered in K12 and O157 are compared in Figure 1B. The most highly enriched protein coding regions (CDS), intergenic regions, and sRNAs are listed in Tables S1A, S1B, and S1C, respectively.

The CRAC data were consistent with interactions established in previous studies on individual RNAs. For example, Hfq crosslinked reads in the *rpoS* mRNA peaked at −215 adjacent to the AAYAA element (−196 to −185, and at −133 adjacent to the U_4_ element (−120 to −123) (Figure 1C), in agreement with in vitro binding sites (Moll et al., 2003; Soper and Woodson, 2008). A similar binding pattern was observed for the *rpoS* leader from CRAC analyses in *E*. *coli* K12 (data not shown). Previous in vitro footprinting of Hfq on *ompA* mRNA demonstrated protection of the SD sequence, the binding site for 30S ribosomal subunits, and the start codon (Moll et al., 2003). In the CRAC data, maximal reads were recovered from the SD at positions −12 to −14 (Figure 1C). The genome-wide Hfq binding profile from a representative data set for O157 is presented in Figure 1D.

### Hfq Targeting: Hfq Preferentially Associates with AGR Trimers and Ribosome Binding Sites in mRNAs

**Figure 2:**
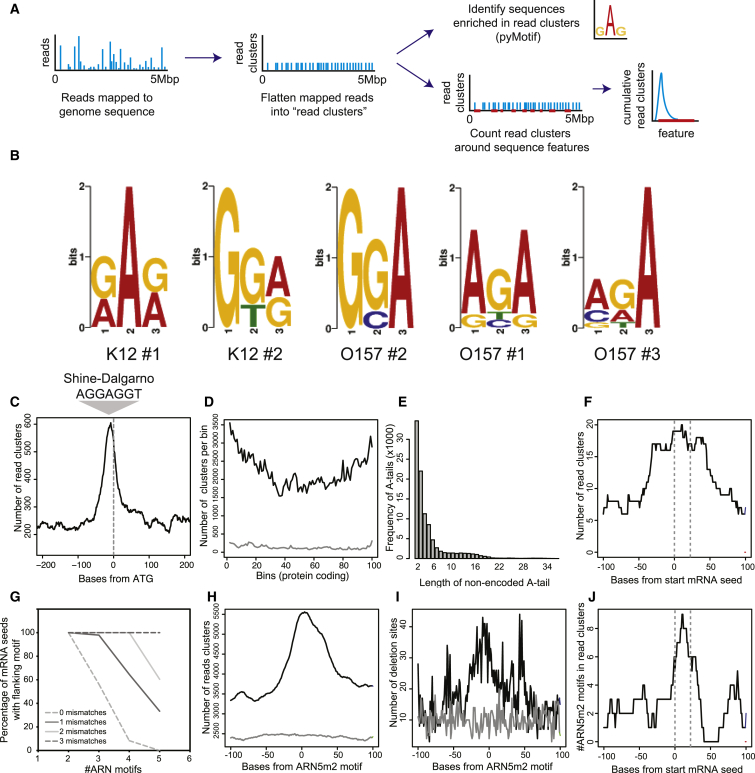
Hfq Binds an ARN Motif Adjacent or Overlapping the mRNA Seed Sequence (A) Workflow for analysis of Hfq crosslinked reads. Mapped reads were flattened into read clusters to prevent bias toward highly enriched sites. Read clusters are analyzed for enriched motifs (as in [B]) or their culmulative distrubution around sequence features such as CDS and mRNA seed regions (as in [C]–[J]). (B) pyMotif from the pyCRAC software package was used to identify trimers that were enriched within RNAs crosslinked to Hfq in five independent experiments. Hfq was crosslinked in either nonpathogenic *E*. *coli* K12 str. MG1655 (K12) or enterohemorhaggic *E*. *coli* O157:H7 str. Sakai (O157). All five logos fit either a repeated AGG or AGA sequence (indicated below). (C) Cumulative Hfq-bound read clusters are plotted relative to the start codon (indicated by gray dashed line). The sequence and approximate position of the Shine-Dalgarno sequence is indicated above. (D) Cumulative Hfq binding within coding sequences. CDS were divided into 100 bins and scored for overlapping read clusters. The cumulative score (genome wide) for each bin is indicated in black and the cumulative score for shuffled CDS coordinates in gray (CDS were assigned random positions within the genome). (E) Frequency of non-genomically encoded oligo(A)-tail length recovered from Hfq-bound reads. (F) Cumulative Hfq-bound read clusters within 100 nt of experimentally verified mRNA seed sequences. Grey dashed lines indicate the position and width for the average mRNA seed. (G) Percent of mRNA seeds with ARN motifs within 100 nt allowing mismatched postions. The x axis represents the number of ARN repeats within a motif, and the y axis represents the percentage of mRNA seeds with that motif within 100 nt. The percentage of mRNA seeds with a flanking ARN motif is plotted for zero to three mismatched postions. (H) Transcriptome-wide cumulative count of Hfq bound read clusters at ARN5m2 motifs (black) and control shuffled ARN5m2 coordinates (gray). (I) Transcriptome-wide cumulative count of deletions in Hfq-bound read clusters at ARN5m2 motifs (indicating direct Hfq contact; black) and control shuffled ARN5m2 coordinates (gray). (J) Position of ARN5m2 motifs within Hfq bound reads at experimentally verified mRNA seed sequences (see also Figure S2 for sequences). Grey dashed lines indicate the position and average width of mRNA seed sequences.

The distal surface of Hfq is proposed to bind repeats of ARN, with one trimer bound in a pocket on each monomer (Link et al., 2009). pyMotif from the pyCRAC software package (Webb et al., 2014) was used to identify trimers enriched within Hfq-bound read clusters (Figure 2A). Analysis of the CRAC data sets identified an overrepresented, purine-rich trimer in each data set that would match repeats of AGA or AGG (Figure 2B). These results are consistent with recognition of an ARN trimer by the distal face of Hfq.

The canonical mechanism of negative regulation by Hfq involves promoting seed sequence binding of an sRNA to an mRNA 5′ UTR to preclude 30S ribosomal subunit association with the SD sequence (Bouvier et al., 2008). In line with this mechanism, a sharp spike in reads was observed 13 (±2.1) nt 5′ to the start codon, corresponding to the consensus SD site (Figure 2C). In addition, binding of sRNAs within the first five codons of the coding sequences (CDS) impedes SD recognition, while interactions further 3′ may affect translation by recruiting the RNA degradosome to the transcript (Bouvier et al., 2008; Pfeiffer et al., 2009). To analyze the distrubition of read clusters across all CDSs, we divided each coding sequence into 100 bins and plotted read cluster density (Figure 2D). Around 39% of recovered reads mapped within CDSs and, of these, 82% (32% of total reads) were outside the 5-codon window for SD inhibition. This indicates that targeting the transcript for cleavage may be the mechanism of repression for approximately one third of Hfq-bound mRNAs.

Transcripts that are targeted for degradation in *E*.*coli* can be oligo(A) tailed by poly(A) polymerase I, providing a single-stranded tail that promotes degradation by 3′→5′ exonucleases (reviewed in Bandyra and Luisi, 2013). Loss of Hfq increases the frequency and length of oligo(A) tails, consistent with functional interactions (Le Derout et al., 2003). Analysis of nonencoded 3′ A tails revealed 5% of sequences crosslinked to Hfq were adenylated, 81% of which carried short oligo(A) tails of 2–6 nt (Figure 2E). This, however, is likely to be an underestimate of the frequency of oligo(A) tails in Hfq-associated RNAs, since these will be detected only if (1) Hfq is bound sufficiently close to the 3′ end of the RNA for their inclusion in short sequence reads, and (2) the nonencoded A sequence is sufficiently short for the remaining sequence to be mapped to the transcriptome.

### Hfq Targeting: Hfq Binds Specific Motifs Within or Overlapping mRNA Seed Sequences

The position of Hfq-bound read clusters was examined around established mRNA-sRNA seed sequences. Read clusters were found to be enriched directly over mRNA seed sequences for 46 experimentally verified interactions (Figure 2F, average mRNA seed size indicated by dashed gray lines) (Beisel et al., 2012; Cao et al., 2010; Corcoran et al., 2012; Sharma et al., 2011) with no clear bias for association 5′ or 3′ to the seed sequence. These results indicated that Hfq binds mRNA targets directly at, or immediately adjacent to, the mRNA seed. We next determined whether mRNA seeds were also associated with an ARN motif. As most Hfq binding sites were identified within 100 nt of the mRNA seed (Figure 2F), we assessed whether repeated ARN motifs were present within this region. A strict ARN4 or ARN3 repeat was present within 100 nt of 3/46 or 23/46 mRNA seeds, respectively (Figure 2G). Allowing a single mismatch in the ARN4 motif (ARN4m1) allowed matches to be found flanking 30/46 mRNA seeds, whereas allowing two mismatches within an ARN5 motif (ARN5m2) matched 31/46 seed sites. Mapping Hfq read density around ARN5m2 motifs transcriptome-wide confirmed strong enrichment relative to random genomic positions (Figures 2H and 2I). Plotting Hfq-bound ARN5m2 motifs relative to mRNA seed sequences showed a clear peak within the seed sequence, confirming that the motif for distal-side binding often overlaps with the mRNA seed (Figure 2J with sequences presented in Figure S2).

We conclude that most sites of Hfq-associated sRNA-mRNA basepairing overlap or are closely associated with a repeated ARN motif in the mRNA, which binds the distal face of Hfq.

### Hfq Targeting: Hfq Binds U-Rich ssRNA Sequences in sRNAs

**Figure 3:**
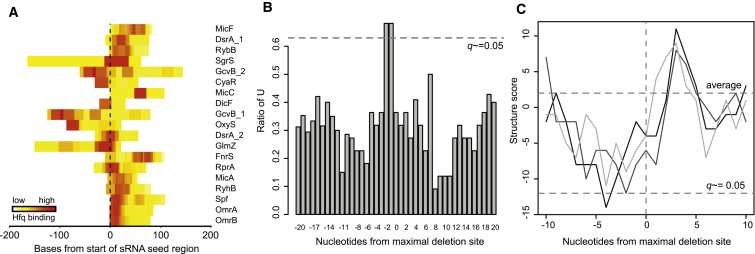
Hfq Binds Single-Stranded, U-Rich Sequences in sRNAs (A) Hfq binding relative to sRNA seed sequences. Small RNAs (indicated right) are aligned to the start of their respective seed regions (dashed line). Each heatmap indicates Hfq binding along the sRNA. (B) A 2U sequence is enriched 5′ of the site of maximal deletions (indicating direct Hfq contact). Positions relative to the site of maximal deletions within 20 Hfq-dependant sRNAs were scored for frequency of a uridine nucleotide. The probability of randomly enriching U at a given position (FDR) is given by the gray dashed line (*q* ≈ 0.05). (C) Hfq is crosslinked to single-stranded nucleotides within sRNAs. The secondary structure of 20 Hfq-dependent sRNAs was predicted using the UNAfold software package and nucleotides surrounding the site of maximal deletions were scored as base paired (+1) or unpaired (−1). The cumulative score for nucleotides from 20 Hfq-dependent sRNAs are plotted against their position relative to the maximal crosslinking site for three independent experiments. False discovery rate is given by the gray dashed line (*q* ≈ 0.05).

The proximal face of Hfq is reported to bind single-stranded A/U rich sequences, which are present in many sRNAs (Ishikawa et al., 2012; Otaka et al., 2011; Sauer and Weichenrieder, 2011; Schumacher et al., 2002). The locations of Hfq-bound read clusters were assessed relative to 21 experimentally verified sRNA seed regions (from 46 seed sequences, overlapping seeds were condensed into a single seed “region”). The Hfq binding peak overlapped the known sRNA seed sequence in a majority of sRNAs (Figure 3A). To examine the sequence and structural requirements for Hfq binding within sRNAs, we examined verified Hfq-dependant sRNAs (22 sRNAs extracted from sRNATarbase) for common features associated with the location of maximum point deletions from the CRAC analysis, as these signify sites of direct Hfq contact. Analysis of nucleotide frequencies revealed strong enrichment for a U-U dinucleotide immediately 5′ to the crosslinking site (Figure 3B). Secondary structure prediction showed that the region 5′ to the crosslinking site was also significantly (*q* < 0.05) enriched for unpaired nucleotides (low values in Figure 3C). In contrast, the region 3′ to the Hfq binding site showed enrichment for basepaired nucleotides. Peaks of Hfq binding were not recovered at Rho-independent terminators. However, the 3′ OH of the U_6_ sequence is in direct contact with Hfq, and UV crosslinking here may inhibit 3′ linker ligation, potentially biasing our results against recovery of poly(U) tails.

We propose that the consensus Hfq binding site on many sRNAs includes a U-U dinucleotide associated with an unpaired region.

### sRNAs Are Encoded within Pathogenicity Islands of EHEC O157

Around 25% of the O157 chromosome is comprised of bacteriophage-derived pathogenicity islands, and 27% of total Hfq-bound reads were mapped to these regions.

To locate noncoding RNAs, we filtered our data for reproducible Hfq targets located antisense to, or >100 bp away from, coding regions (see Supplemental Experimental Procedures). This analysis identified 63 unannotated, potential noncoding sRNAs within the O157 transcriptome. Eight of these were encoded within the core genome and 55 within pathogenicity islands (Figure 1D, genomic positions of predicted sRNAs are indicated in red; Table S2). One sRNA expressed from the pathogenicity islands of EHEC has been described, Esr41 (Sudo et al., 2014). Pathogenicity islands are enriched for predicted sRNA genes relative to the core genome, with an average of 39 sRNA per Mb of accessory genome and 23 sRNAs (Keseler et al., 2013; Raghavan et al., 2011) per Mb of core genome.

Rho-independent termination is a common feature for many sRNAs and was shown to contribute to Hfq binding in some cases (Otaka et al., 2011; Sauer and Weichenrieder, 2011). The RNAmotif descriptor for *E*. *coli* Rho-independent terminators was used to identify terminator loops within 200 nt of the 3′ edge of the Hfq binding site (see Supplemental Experimental Procedures). Thirty-one of the sRNAs identified in this study were predicted to carry Rho-independent terminators (Table S2).

**Figure 4:**
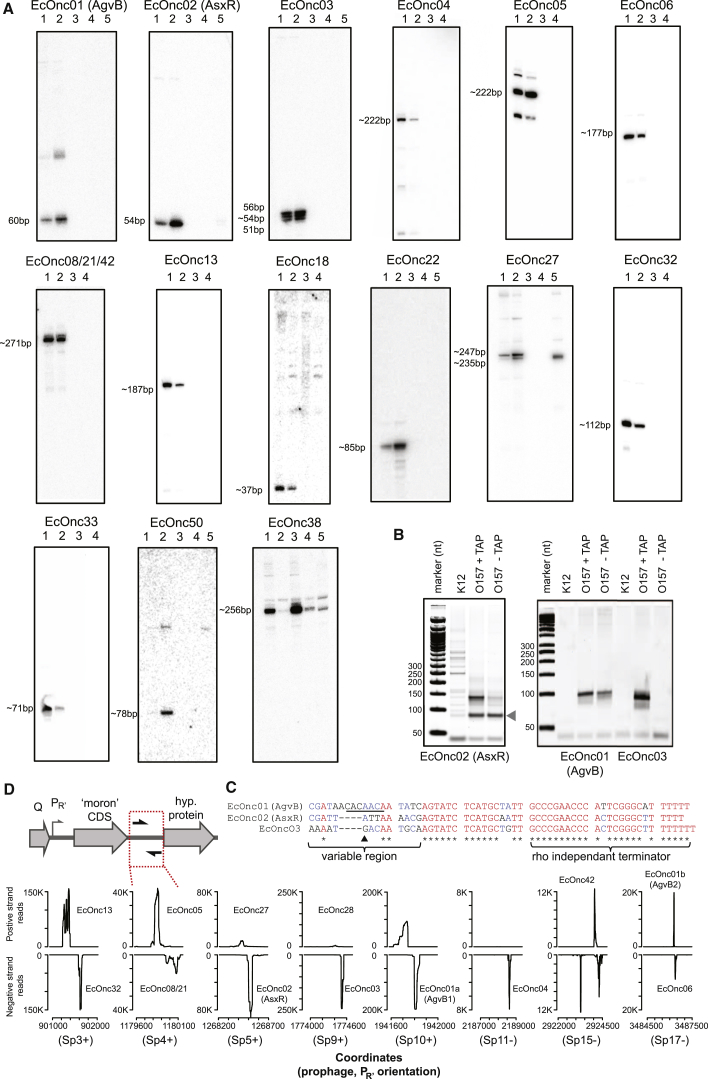
Identification of Prophage-Encoded sRNAs in *E*. *coli* O157 (A) Northern blot analysis of predicted sRNAs (also see Table S2) in *E*. *coli* O157:H7 str. Sakai (O157) and nonpathogenic *E*. *coli* K12 (K12) cultured under virulence inducing conditions (MEM-HEPES) and in LB broth. Lane 1: O157 grown in MEM-HEPES; lane 2: O157 grown in LB; lane 3: K12 grown in MEM-HEPES; lane 4: K12 grown in LB; lane 5 (where applicable): O157Δ*hfq* grown in LB. Approximate size of RNAs indicated left of blot. (B) 5′ RLM-RACE with and without tobacco acid pyrophophatase (TAP) treatment of EcOnc01–EcOnc03. Grey arrow indicates a primer dimer. (C) Prophage encode convergent sRNAs within the “moron” insertion site at P_R’_. (Top) Graphical representation of gene organization at the moron CDS insertion site showing the phage regulator, antiterminator Q CDS, and promoter P_R’_. Moron CDSs are inserted downstream of P_R’_, and convergent sRNAs are encoded between the moron CDS and a conserved hypothetical phage ORF. (Bottom) Hfq-bound reads are plotted for the intergenic region between moron CDS and downstream hypothetical ORF (indicated by red box above) for prophages encoding convergent sRNAs. Prophage designation and strand encoding P_R’_ are given in brackets. Peaks that have been assigned to predicted sRNA are indicated. (D) Alignment of EcOnc01–3. Underlined sequence in EcOnc01 corresponds to the GcvB targeting consensus. The black triangle indicates the shortest alternate 5′ triphosphate end detected by 5′RLM-RACE in EcOnc03.

Northern blot analysis confirmed the expression of 17 predicted sRNAs (from 18 tested), with sizes ranging from approximately 37–354 nt (Figure 4A). sRNAs are commonly destabilized in the absence of Hfq, and eight of these confirmed sRNAs (the four most abundant, three encoded on StxΦ, and a core encoded sRNA) were characterized in a Δ*hfq* background. Six sRNAs were destabilized by loss of Hfq, one sRNA was approximately 4 nt shorter, and one sRNA was stable (Figure 4A; Table S2). The abundance of one of the eight unannotated core genome sRNAs, EcOnc38, was low in LB medium but higher in MEM-HEPES, suggesting that it may have escaped previous detection due to poor expression.

### Prophages Encode a Class of Unusually Short sRNA

The unannotated sRNAs most frequently recovered by Hfq CRAC in *E*. *coli* O157 were EcOnc01, EcOnc02, and EcOnc03. The 5′ ends of these transcripts were mapped to identify primary transcription start sites (Figures 4B and 4C), confirming they encode unusually short sRNAs between 51 and 60 nt. For EcOnc03, heterogeneous triphosphorylated 5′ ends were detected (between the 5′ end and black arrow in Figure 4C), consistent with northern blot detection of three distinct RNA species. These, and several other sRNAs, were expressed from genes located at conserved locations within lambdoid prophages. The sequence downstream of the bacteriophage Q antiterminated P_R’_ promoter tolerates DNA insertions termed “morons” (more DNA or more “ome”) (Juhala et al., 2000) and carried convergent sRNA genes. This is exemplified by the bacteriophage Sp5 that encodes Shiga toxin 2 at a moron insertion site (Figure 4D; see plot for Sp5), where convergent sRNAs (EcOnc02 and EcOnc27) are encoded 3′ of the *stx2B* gene. A similar gene organization was seen for other lambdoid prophages Sp3, Sp4, Sp9, Sp10, Sp11, Sp15, and Sp17 (Figure 4D). Many of the sRNAs encoded at these positions fall into related groups but are not identical. The four most abundant sRNAs, EcOnc01a, EcOnc01b, EcOnc02, and EcOnc03 (encoded within Sp10, Sp17, Sp5, and Sp9, respectively) share highly conserved 3′ regions of ∼42 nt but have variable 5′ regions of 14–18 nt (Figure 4C).

### EcOnc02 Is Encoded within the Stx2Φ and Derepresses a Heme Oxygenase

The gene encoding EcOnc02 is located 282 bp 3′ and antisense to *stx2AB*, which encodes the major virulence factor Shiga toxin 2. Analyses of EcOnc02 and EcOnc01 (below) indicate that these represent a class of “anti-sRNAs,” and we have renamed EcOnc02 as AsxR. To identify functional targets, AsxR was transiently overexpressed (10 min pulse) and changes in mRNA abundance were monitored using oligonucleotide microarrays. To identify directly regulated targets, transcripts showing altered abundance were screened for the presence of Hfq binding sites within 200 nt of the CDS (Figure S3A). *chuS* and *chuW* were each found to be more abundant after a 10 min pulse of AsxR transcription and associated with Hfq by CRAC analysis.

**Figure 5:**
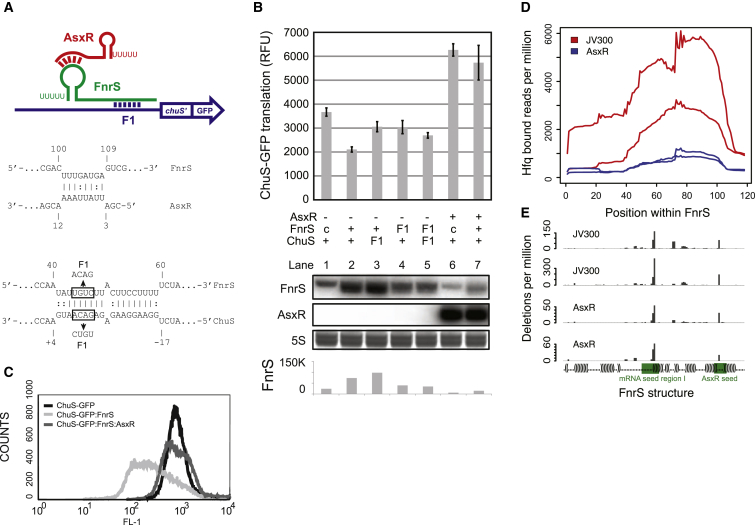
The Shiga Toxin 2 Locus Encodes an Anti-sRNA that Enhances Expression of the Heme Oxygenase ChuS (A) (Top) Graphical representation of interactions between AsxR, FnrS, and the *chuS* mRNA. F1 indicates the positions of the complementary mutation. (Bottom) Predicted base paring (IntalRNA software) between AsxR and FnrS, and FnrS and the *chuS* transcript. Boxes and arrows indicate sequence changes that were introduced into F1 mutants. (B) (Upper panel) Fluorescence of the 3′ chuA→5′ chuS *chuS*-GFP translational fusion was monitored in the presence of FnrS, AsxR, and appropriate point mutants (indicated below bar chart; basal levels of chromosomal FnrS are indicated by “c”). (Lower panel) Northern blot analysis of FnrS and AsxR (indicated). SYBR-green-stained 5S rRNA (5S) is included as a loading control. (Bottom) Quantification of FnrS northern blots by densitometry. Error bars indicate SEM. (C) Flow cytometry quantification of fluorescence from cells expressing *chuS*-GFP alone, with FnrS, or with both FnrS and AsxR. (D) AsxR reduces Hfq-bound FnrS. The *chuS*-GFP fusion and FnrS were constitutively expressed in *E*. *coli* MG1655 *hfq*-HTF with AsxR (blue) or the control plasmid pJV300 (red) and CRAC performed on these strains. Replicate data sets are plotted as reads per million across FnrS. (E) Hfq binds to both seed and 3′ loop regions of FnrS. Deletions per million Hfq-bound reads are plotted relative to secondary structure of FnrS. Major deletion sites are located within the mRNA seed region I (green) and the AsxR seed region (green) within the terminator loop. See also Figure S3.

The *chuS* gene encodes a heme oxygenase and lies downstream of *chuA,* which encodes a heme outer-membrane receptor. The predicted 5′ UTR and *chuAS* region was cloned into the GFP fusion vector pXG10-SF to monitor translation. Translation of *chuAS* was increased 2.5-fold in the presence of AsxR, consistent with our microarray analysis (Figures S3B and S3C; *chuAS* samples). In order to identify the minimal sequence requirements for increased translation of *chuS*, regions of the *chuAS* transcript were subcloned into the GFP fusion vector pXG30-SF that provides an upstream coding sequence (*lacZ*′) to allow translational coupling (Corcoran et al., 2012). A 155 nt transcript, extending from the *chuA* stop codon to +66 nt of *chuS*, had 2.3-fold more translation in the presence of AsxR (Figures S3B and S3C). This region lacks complementarity to AsxR, suggesting that AsxR might function indirectly via a regulator that binds directly to this 155 nt fragment. IntaRNA software was used to screen for putative interactions with known sRNA regulators and revealed extensive complementarity between the sRNAs RyhB and FnrS and the SD site of *chuS*. Constitutive expression of RyhB or FnrS repressed translation of the ChuS fusion reporter (data not shown). AsxR lacks clear complementarity to RyhB, but its 5′ end could potentially basepair to the single-stranded loop of the Rho-independent terminator of FnrS (Figure 5A). Furthermore, an interaction between AsxR and FnrS is consistent with our FnrS-Hfq CRAC data, which showed two prominent peaks of deletions within FnrS; one maps to the known seed site for mRNA binding (Durand and Storz, 2010) and another within the terminator stem loop.

A three-plasmid system was used to monitor the roles of FnrS and AsxR in controlling translation of a construct containing nts −112 to +66 relative to the ChuS start codon fused to GFP (Figure 5B). Translation of ChuS was repressed by expression of FnrS (Figure 5B, lanes 1 and 2), which was partially relieved by mutation (F1) of either FnrS or ChuS (Figure 5B, lanes 3 and 4). Coexpression of AsxR relieved the repression of ChuS translation by FnrS (Figure 5B, upper panel, lane 7), and this was confirmed using flow cytometry (Figure 5C). Basal translation of the ChuS-GFP fusion was increased in the presence of AsxR alone, as seen for the vector expressing the entire *chuAS* region (Figure 5B, lane 6), indicating that ChuS translation is repressed by endogenous FnrS.

Northern analysis showed that the level of FnrS is reduced in the presence of AsxR, consistent with AsxR binding to the terminator stem, which is required for stability of the 3′ end of FnrS (Figure 5B, lower panel, compare lanes 2 and 7) (Blum et al., 1999; Cisneros et al., 1996; Figueroa-Bossi et al., 2009). Compensatory 3 nt mutations (S1) were introduced into the FnrS 3′ stem loop and the 5′ region of AsxR, but both S1 mutations were strongly destabilizing. We additionally performed Hfq-CRAC analysis on the three-plasmid system using the *E*. *coli* str. MG1655 *hfq*-HTF background (lacking both *chuS* and *asxR*). ChuS-GFP and FnrS were constitutively expressed in the presence of AsxR or the control plasmid pJV300. Consistent with our northern analysis, the association of FnrS with Hfq was strongly reduced in the presence of AsxR (Figure 5D). Deletions identify precise Hfq binding sites and mapped to both the mRNA seed region I and to the single-stranded loop of the Rho-indpendant terminator (Figure 5E). These interactions were detected in the presence or absence of AsxR, indicating that Hfq contacts the terminator loop under both conditions. We conclude AsxR acts to increase expression of the ChuS heme oxidase via destabilization of FnrS.

### EcOnc01 Functions as an Anti-sRNA that Antagonises GcvB

**Figure 6:**
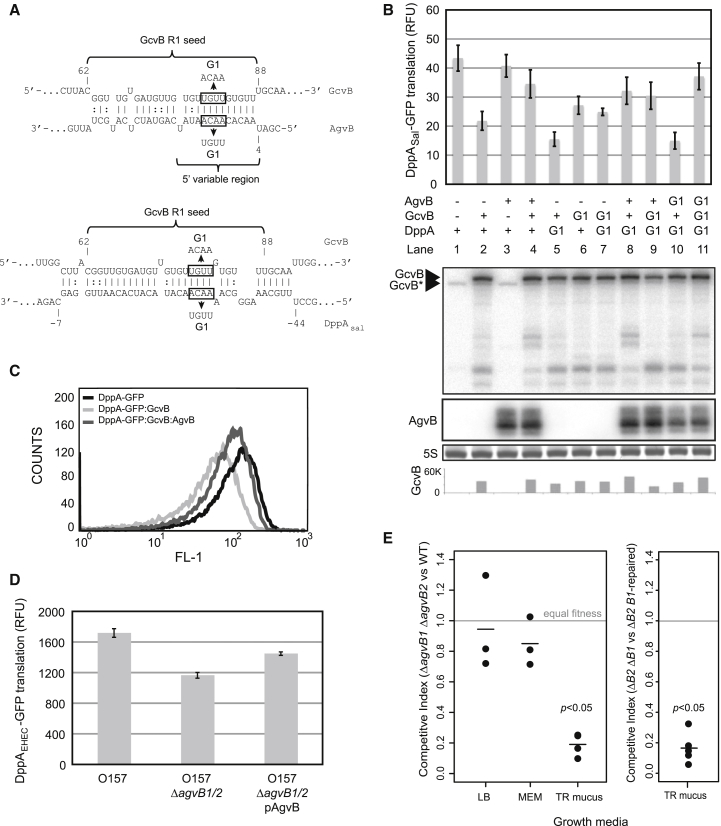
EcOnc01 (AgvB) Acts as an “Anti-sRNA” to Inhibit GcvB Repression (A) Interactions between GcvB and AgvB (top) and GcvB and DppA_Sal_ (bottom) were predicted using IntaRNA software. The R1 seed sequence of GcvB is indicated in braces, and sequences that were introduced into G1 mutants are indicated within boxes. (B) Fluorescence of DppA_sal_-GFP was used to monitor GcvB activity in the presence of AgvB, GcvB, and G1 mutants. Genotypes for each reading are indicated below. (Below: GcvB and AgvB) Northern analysis of GcvB and AgvB, respectively. GcvB^∗^ indicates the endogenous copy of GcvB, which carries an 8 nt deletion in the R1 seed region. SYBR-green-stained 5S rRNA (5S) is shown as a loading control for GcvB and AgvB northern blots. The bottom panel shows quantification of the exogenous copy of GcvB by densitometry. Error bars indicate SEM. (C) Flow cytometry quantification of fluorescence from individual cells expressing DppA_sal_-GFP alone, with GcvB, or with both GcvB and AgvB. (D) Fluorescence of DppA_EHEC_-GFP was used to monitor translation efficiency of DppA in *E*. *coli* O157:H7, Δ*agvB1* Δ*agvB2*, and the complemented strain Δ*agvB1* Δ*agvB2* pZE12::EcOnc01 (pAgvB). (E) The left-hand panel shows the competitive indices of *E*. *coli* O157 Δ*agvB1* Δ*agvB2* against the parent stain (Sakai) grown in LB media (n = 3), MEM-HEPES media (MEM, n = 3), and terminal rectal mucus (TR mucus, n = 4). The right-hand panel shows the competitive indices for the double mutant *E*. *coli* O157 Δ*agvB1* Δ*agvB2* against the same strain complemented on the chromosome with *agvB1* (TR mucus, n = 5). A competitve index of 1 indicates no fitness difference; <1 indicates a fitness disadvantage.

We noted that the 5′ variable domains of the most abundant unannotated sRNAs recovered, EcOnc01a and EcOnc01b, contain the consensus target sequence for the R1 seed sequence of the core genome-encoded sRNA, GcvB (CACAACA; underlined Figure 4C) (Sharma et al., 2011). In silico predictions support the potential for EcOnc01 to bind the R1 seed sequence of GcvB (Figure 6A), and we have renamed EcOnc01 *a*nti-sRNA for *G*c*vB* (AgvB).

To test for interactions between AgvB and GcvB, we used a GFP translational fusion to the dipeptide transporter DppA mRNA from *Salmonella* Typhimurium, as this is known to be repressed by GcvB (Sharma et al., 2007; Urbanowski et al., 2000). Our three-plasmid system was used to express AgvB, GcvB, and DppA in *E*. *coli* Top10F′, which we found to have an 8 nt deletion in the R1 seed sequence of the endogenous copy of GcvB, inactivating the chromosomal copy of the GcvB R1 seed. Overexpression of GcvB was found to be toxic and induction from P_LtetO-1_ was reduced until growth was restored. Expression of GcvB inhibited translation of DppA mRNA (Figure 6B, lanes 1 and 2), whereas coexpression of AgvB with GcvB restored DppA translation (Figure 6B, upper panel, lane 4). These results were confirmed by flow cytometry (Figure 6C). AgvB had no significant effect on DppA expression in the absence of GcvB (Figure 6B, lanes 1 and 3). To determine whether AgvB interacts directly with GcvB, base changes (designated G1) were introduced into AgvB, GcvB, and DppA mRNA (Figure 6A). The G1 mutation in DppA was insufficient to destabilize the GcvB-DppA interaction, as DppA-G1 was repressed by GcvB. However, direct interaction between GcvB and DppA at the R1 seed has been rigorously demonstrated using a GcvB ΔR1 mutant and footprinting, indicating that the 4 nt G1 mutation is insufficient to destabilize the long R1 pairing (Sharma et al., 2007). Similarly, a G1 mutation in GcvB was insufficient to relieve DppA repression, although repression by GcvB-G1 was slightly reduced likely due to mutation of an ACA-motif required for optimal translation (Figure 6B, lanes 5–7) (Yang et al., 2014). However, the G1 mutation within the 5′ variable region of AgvB was sufficient to prevent the derepression of DppA, evident when comparing lanes 5, 8, and 10 (Figure 6B). Depression by the modified anti-sRNA (AgvB-G1) was restored when AgvB-G1 was expressed in the context of GcvB-G1 and DppA-G1 (Figure 6B, comparing lanes 7, 9, and 11). Northern analysis did not indicate a significant reduction in the level of GcvB following coexpression of AgvB (Figure 6A, lower panel). We conclude that AgvB antagonizes GcvB function by hybridizing to the seed region and blocking its interactions with target mRNAs.

To verify the anti-GcvB function of AgvB in the pathogenic background, we deleted both copies (EcOnc01a and EcOnc01b) from *E*. *coli* O157:H7 str. Sakai. The translation efficiency of DppA mRNA was measured using a constitutively transcribed GFP fusion to the 5′ UTR and the first 10 codons of DppA from *E*. *coli* O157:H7. Deletion of both *agvB1* and *agvB2* resulted in a 32% reduction in translation of DppA, and complementation of the mutant using constitutively transcribed AgvB restored translation by 24% relative to the mutant (Figure 6D). These results demonstrate that AgvB modulates translation of DppA in pathogenic *E*. *coli* O157:H7.

*E*. *coli* O157:H7 colonizes the final few centimeters of the bovine gastrointestinal tract, with the majority of bacteria multiplying in the terminal rectal mucus (TRM) (Naylor et al., 2003; Tildesley et al., 2012). As such, TRM recovered from this site can be used as a relevant growth medium in place of in vivo experiments in cattle. To investigate the potential benefit of regulation by AgvB, competitive index experiments were carried out between the WT strain and the double deletion, Δ*agvB1* Δ*agvB2*, in standard laboratory media (LB and MEM-HEPES) and in TRM. The double deletion of AgvB did not significantly affect growth in the two laboratory media, whereas loss of the sRNA strongly reduced the competitiveness of the strain in TRM (Figure 6E). This result was confirmed by chromosomal complementation of *agvB1* into the double deletion strain followed by competition of the complement against the double deletion strain in TRM. The single complement successfully outcompeted the double mutant (Figure 6E), and we conclude that the pathogenicity island-associated sRNA AgvB aids growth within its animal host reservoir at the specialized site colonized by this pathogen.

### AgvB Interacts with the mRNA Binding Face of Hfq and Forms a Stable Duplex with GcvB

**Figure 7:**
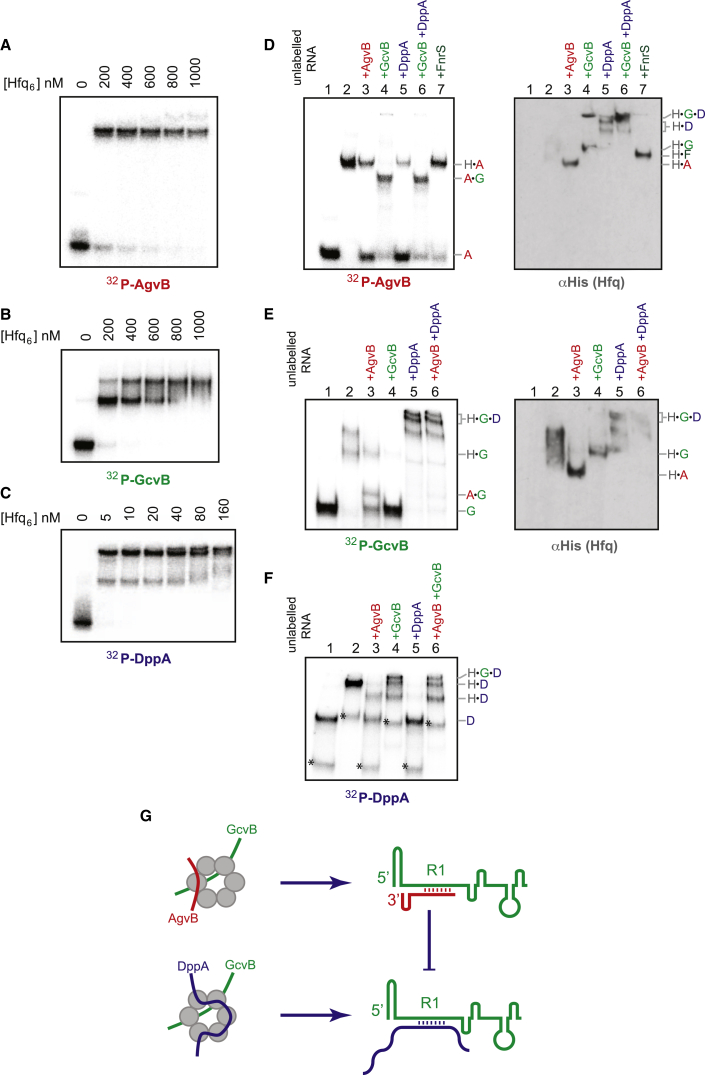
EMSA Analysis in Hfq-AgvB Interactions (A–C) Approximately 40 fmol of in-vitro-transcribed, radiolabeled AgvB (A), GcvB (B), or the 5′ 166 nt of DppA_Sal_ (C) were incubated with increasing amounts of Hfq_6_ (indicated above). (D–F) (Left panels) Competition assays with unlabelled RNAs. Radiolabelled AgvB (D), GcvB (E), or DppA (F), were incubated in the absence (lane 1) or presence of 500 nM Hfq_6_ (AgvB and GcvB) or 50 nM Hfq_6_ (DppA) (lanes 2–7). Hfq binding reactions were additionally incubated in the presence of a 50-fold excess of unlabelled competitor RNAs (indicated above gel, lanes 3–7). The composition of complexes is indicated on the right-hand side (H = Hfq, A = AgvB, G = GcvB, and D = DppA). For radiolabeled DppA (F), a shorter DppA RNA fragment copurified with the full-length product and is indicated by an asterisk. (D and E) (Right panels) αHis western blot analysis of EMSA gels to monitor the presence of His_6_-tagged Hfq in gel-shifted complexes. Lanes are as in the left panels. In lanes E2 and E3, Hfq migrates as a smear, probably because it copurifies with heterogenous RNA species (Sittka et al., 2008), which are displaced in the presence of higher added concentrations of RNAs. In Figure 7F, the low Hfq concentration (50 nM) was not detectable by western analysis in DppA EMSA gels. (G) Model for interaction of AgvB with Hfq, GcvB, and DppA. AgvB binds the distal face of Hfq (see also Figure S4) and forms a duplex with the R1 region of GcvB. Occlusion of the R1 region of GcvB prevents interactions between GcvB and the mRNA DppA. AgvB may also displace DppA from Hfq, although this interaction would be expected to be much more transient than inhibition through occlusion of GcvB R1. In the absence of AgvB, Hfq facilitates duplex formation between DppA and GcvB, repressing translation of DppA.

To understand the mechanism of AgvB-mediated translation derepression, we characterized the interactions between Hfq, AgvB, GcvB, and DppA mRNA. Gel mobility shift analyses of complexes formed in vitro demonstrated that AgvB and GcvB bound Hfq with a comparable affinity (Figures 7A and 7B). Strikingly, the 5′ 166 nt of DppA mRNA bound Hfq with around 10-fold higher affinity (Figure 7C). We then used pairwise competition experiments to characterize interactions with Hfq (Figures 7D and 7E). Addition of excess GcvB to Hfq-DppA binding reactions shifted the labeled DppA complex into higher molecular weight ternary complexes (Figure 7F, lane 4, labeled “H⋅G⋅D”), and this was also observed for labeled GcvB in the presence of excess DppA (Figure 7E, lane 5, H⋅G⋅D). Excess AgvB competed labeled DppA from Hfq, despite the higher apparent affinity of DppA binding (Figure 7F, lane 3), and DppA was able to compete labeled AgvB from Hfq (Figure 7D, lane 5). This strongly indicates that DppA and AgvB bind the same site on Hfq. We had observed that ARN4m1 or ARN5m2 motifs were present in a majority of bone fide Hfq distal face binding sites (Figure 2G) and also identified ARN4m1 and ARN5m2 motifs within the 5′ variable region of AgvB and AsxR (Figure S4). These results indicate that AgvB and DppA both interact with the distal RNA binding site of Hfq, potentially facilitating annealing with complementary RNAs bound to the proximal face. The addition of GcvB to labeled AgvB binding reactions did not compete AgvB into free RNA but shifted AgvB into a faster migrating complex (Figure 7D, lane 4, and 7E, lane 3, complex A⋅G) that was detected with both labeled GcvB and AgvB, but lacked detectable Hfq (western blots in Figures 7D and 7E, right panel). The most likely composition of the faster migrating band is a stable AgvB-GcvB duplex. A similar duplex was not formed with excess of an sRNA that does not have complementary to AgvB, FnrS (Figure 7D, lane 7).

We conclude that AgvB and DppA compete for binding to the distal face of Hfq, whereas a stable duplex is formed between the sRNA, GcvB, and its anti-sRNA, AgvB.

## Discussion

In mammalian cells, viruses use miRNAs and other RNAs to modulate the host miRNA population. The data presented here demonstrate that bacteriophages and bacteriophage-derived pathogenicity islands express sRNAs that modulate the activities of bacterial host sRNAs. We predicted that these anti-sRNAs alter cell metabolism to favor bacterial colonization of specific host or environmental niches and confirmed this for AgvB.

The majority of Hfq-associated mRNA reads were crosslinked outside of protein coding sequences with a sharp spike in binding at the SD site, consistent with occlusion of the SD by sRNAs. The SD site has a purine-rich motif with consensus AGGAGGT, matching the most overrepresented Hfq-binding trimer in vivo (AGR). Hfq bound read clusters were also enriched at U-rich motifs in sRNAs and at experimentally verified mRNA-sRNA seed interactions on both the mRNAs and sRNA (Figure 3A). The majority of seed-binding sites in mRNAs were also associated with multiple ARN motifs, the consensus motif for binding the Hfq distal face. Hfq binds single-stranded RNA, suggesting that binding of Hfq to the mRNA seed region is in competition with duplex formation between the sRNA and mRNA. Such competition would ensure a minimum free energy threshold for hybridization and provides a simple mechanism allowing Hfq to add stringency to the limited sequence requirements for base pairing between sRNAs and mRNAs. Since the Hfq distal-side binding motif in the mRNA seed is sequestered in sRNA-mRNA duplexes, target acquisition by sRNAs would lead to rapid dissociation of Hfq from the ternary complex, as previously observed (Fender et al., 2010; Hopkins et al., 2011; Lease and Woodson, 2004; Updegrove et al., 2008). This would also prevent duplexed mRNAs from reassociating with Hfq and competing with unpaired mRNAs.

### Hfq and Xenogenic sRNA

*Escherichia coli* O157:H7 str. Sakai shares a common “core” genome of 4.1 Mb with the commensal isolate *E*. *coli* K12 (Hayashi et al., 2001). The majority of pathogenicity determinants are encoded within an extra 1.4 Mb of horizontally acquired DNA elements, including active and cryptic prophages, and Hfq binding sites were identified throughout these domains. Overall the density of predicted sRNA genes in pathogenicity islands is ∼1.8-fold greater than in the core genome.

The four most abundant unannotated sRNAs identified in this study were homologous and encoded at conserved positions within convergent sRNA pairs, 3′ of P_R’_ in the so-called “moron” insertion site of lambdoid prophages. We have called this group of RNAs “anti-sRNA,” as two members tested antagonize the function of core genome encoded sRNAs. All four anti-sRNA were between 51 and 60 nt in length, with highly conserved 3′ regions (nucleotides ∼18–60) and variable 5′ ends. We initally examined AsxR (EcOnc02), as this is encoded 3′ and antisense to the Shiga toxin 2 transcript. Shiga toxins are responsible for the cellular pathology that leads to capillary damage and hemorrhage in EHEC-infected individuals that can lead to potentially fatal HUS.

Pulsed expression of AsxR stablised *chuS* mRNA, which encodes a heme oxygenase required for release of iron from heme. The core genome-encoded sRNA, FnrS, repressed *chuS* translation and was destabilized in the presence of AsxR. The 5′ region of AsxR is complementary to the single-stranded loop of the FnrS Rho-independent terminator. FnrS was destabilized by AsxR, consistent with AsxR hybridization unfolding the terminator stem that protects the 3′ end from exonucleolytic attack (Blum et al., 1999; Cisneros et al., 1996; Figueroa-Bossi et al., 2009). A similar mechanism of sRNA destabilization has been proposed for ChiX (MicM), an sRNA that is destabilized by an intercistronic region of the *chbBC* transcript with complementarity to the terminator stem of ChiX (Figueroa-Bossi et al., 2009).

FnrS is likely to be transcribed under the predominately anaerobic conditions of the gastrointestinal tract lumen, repressing ChuS translation. We suggest that expression of AsxR from the Shiga-toxin-2-encoding bacteriophage derepresses ChuS, potentially under the microaerophilic conditions associated with the epithelium to which the bacteria attach. The presence of AsxR within the *stx2AB* locus suggests that coordinating heme release and uptake by the lytic and lysogenic bacterial populations, respectively, are selected, coinherited traits. Such anti-sRNA regulation adds to the ways in which an integrated prophage can modify expression in the host bacterium and impact on colonization and disease (Xu et al., 2012).

The 5′ variable region of the most abundant anti-sRNA, AgvB, matches the consensus binding motif (CACAACA) for the core sRNA GcvB R1 seed region. GcvB is a key regulator of amino acid catabolism and transport (Sharma et al., 2011), repressing translation of numerous proteins, including the dipeptide transporter DppA. Expression of AgvB in *E*. *coli* K12 did not appreciably destabilize GcvB, but it relieved translational repression of DppA in reporter constructs. Loss of AvgB from *E*. *coli* O157:H7 reduced the translation efficiency of DppA_EHEC_, indicating that AgvB indeed modulates translation in the pathogen.

AgvB fits the model of a small RNA and might have been expected to interact with the proximal face of Hfq through its Rho-independent terminator and/or U-U motif 5′ of the terminator stem. However, gel mobility shift analysis indicated that AgvB and DppA mRNA were able to displace each other from Hfq. DppA is strongly predicted to associate with the distal face of Hfq, suggesting that this is also the case for AgvB. Sequence analysis of AgvB identified a distal face binding motif (ARN4m1 and ARN5m2) within its 5′ variable region, and Hfq binding at this site is supported by CRAC data and the observation that AgvB is partly destabilized by introduction of a G1 mutation into this motif (Figure 6B, AgvB northern, lanes 10 and 11). In contrast, ternary complex formation was seen between Hfq, GcvB, and DppA mRNA. This indicates that these RNAs bind distinct, proximal and distal, sites on Hfq, potentially favoring duplex formation, using the rim arginines to reduce electrostatic repulsion (Panja et al., 2013). Consistent with in vitro duplex formation between AgvB and GcvB was facilited by Hfq (Figures 7D and 7E).

Riboregulation is an important posttranscriptional process generally responding to environmental conditions and therefore critical for adaptation to specific niches, including those encountered during colonization of the mammalian host by pathogenic bacteria. Horizontal acquisition of genomic regions by phage transfer endows the recipient bacterium with new genomic material, including genes that control “core” genome function. Two copies of AgvB are maintained in *E*. *coli* O157:H7, and deletion of both copies of AgvB reduced the competitiveness of the strain in mucus from the bovine terminal rectum, but not in rich (LB broth) or minimal (M9) media. The terminal rectum is the main colonization site for the bacterium in the reservoir host, supporting a function for the anti-sRNA in colonization of this specific niche. The characterized AgvB target, GcvB, is a global regulator that controls translation of up to 1% of transcripts. The majority are associated with amino acid and peptide uptake systems (Sharma et al., 2011), but the GcvB target(s) that contribute to enhanced growth at this site remain to be established.

The identification of “anti-sRNAs” has defined another layer of gene expression control in bacteria and a regulatory process that is important for niche adaptation in pathogenic *E*. *coli*.

## Experimental Procedures

### Strain and Plasmid Construction

Strains used in this study are listed in Table S3A. *E*. *coli* O157:H7 str. Sakai stx− is a Shiga toxin negative derivative of the sequenced isolate O157:H7 str. Sakai (NCBI genome accession number NC_002695.1). For genetic manipulations, strains were grown in LB broth or plates supplemented with ampicillin (50 μg/ml), kanamycin (50 μg/ml), tetracycline (15 μg/ml), or chloramphenicol (25 μg/ml) where appropriate. The HTF tag contains His_6_, a TEV protease cleavage site, and 3×FLAG affinity tag. Chromosomal replacement of *hfq* with *hfq*-HTF in both *E*. *coli* strains was carried out by allelic exchange, as was deletion of both copies of *agvB* from *E*. *coli* O157 str. Sakai. To monitor sRNA and anti-sRNA activity on translation of specific genes, a three-plasmid system was used with GFP translational fusion to the open reading frame of interest. Point mutations were introduced into the sRNA, anti-sRNA, or mRNA sequence by PCR amplification using mutagenic primers. Full descriptions are provided in the Supplemental Experimental Procedures section in Supplementary Information.

### UV CRAC

Hfq CRAC was performed essentially as described by Granneman et al. (2011), except cell lysates were initially purified over anti-FLAG M2 affinity gel (Sigma, A2220). In summary, *E*. *coli* expressing the chromosomal Hfq-HTF was cultured under the required conditions and then subjected to UV irradiation in a stainless steel cylinder for 90 s. Cells were harvested and disrupted and Hfq-RNA complexes were purified on an anti-FLAG resin. The complexes were cleaned, treated with TEV protease, and trimmed with RNase before a second round of purification under guanidine hydrochloride denaturing conditions using Ni-NTA resin. Linker and 5′ ^32^P labeling were carried out followed by gel electrophoresis, complex purification, and Protease K digestion. Released RNA was revese transcribed, the cDNA amplified by PCR, and the products separated by gel electrophoresis. Products over primer-dimer size were extracted and sequenced. Full details of this CRAC procedure are provided in the Supplemental Information. The Pearson correlations ranged from 0.49 to 0.95 between experiments. For K12, 93% of read clusters overlapped between the experiments, although the Pearson correlation was less significant (0.31), probably due to a lower number of sequences in one replicate. 5′ RLM-RACE was used to map the 5′ end of transcripts and to distinguish primary triphosphate from monophosphorylated 5′ ends. Full details of the in silico analysis of Hfq crosslinked sequences, including motif analyses, experimentally verified mRNA and sRNA seed sequence analyses, identification of unannotated sRNA sequences, and in silico prediction of sRNA and anti-sRNA targets, are provided in the Supplemental Experimental Procedures within the Supplementary Information.

### Microarray Analysis of AsxR

For pulsed expression studies, *E*. *coli* O157:H7 str. TUV93-0 (deleted for both Stx phage) harboring pBAD+1 or pBAD+1::AsxR was grown to OD_600_ 0.8 in MEM-HEPES media and induced with 0.2% L-arabinose for 10 min. Microarray analysis was performed essentially as previously described (Tree et al., 2011).

### Northern Blots

Total RNA was extracted by GTC-Phenol extraction. Five micrograms of total RNA was separated on an 8% polyacrylamide TBE-Urea gel and transferred to a nylon membrane and UV crosslinked. Membranes were prehybridized in 5 ml of UltraHyb Oligo Hyb (Ambion) and probed with 10 pmol of ^32^P end-labeled 35-mer DNA oligo (Table S3C).

### Fluorescent Reporters of Translation

The three plasmid system for expression of GFP and superfolder GFP translational fusions in anti-sRNA and sRNA expressing backgrounds were performed in *E*. *coli* DH5α, for *chu* operon and fragment fusions, and *E*. *coli* Top10F′ for DppA_Sal._ Cultures were grown overnight in LB before inoculation into M9 or MEM-HEPES at a 1:100 dilution. Fluorescence was measured either using an Infinite M200 microplate reader (Tecan) or a FLUOstar Optima fluorescence plate reader (BMG Labtech, Germany) with fluorescence measurements normalized to OD_600_.

### Electrophoretic Mobility Shift Assays

For analysis of Hfq binding to single RNAs, ∼40 pmol of labeled RNA was incubated with increasing Hfq in 1× Binding Buffer (10 mM Tris-HCl [pH 7.4], 0.1 mM EDTA, 10 mM NH_4_Cl, 10 mM NaCl, and 10 mM KCl), 1 mg/ml tRNA, and 4% glycerol + bromophenol blue. Reactions were incubated at room temperature for 5 min and separated on a native 5% polyacrylamide 0.5× TBE gel. For analysis of complexes formed in the presence of multiple RNAs, binding buffer was replaced with Duplex Buffer (40 mM Tris-Acetate, 0.5 mM Magnesium Acetate, and 100 mM NaCl). 0.5× TBE was also replaced with 1× Duplex Buffer in both native 5% polyacrylamide gels and running buffer. Approximately 40 pmol of ^32^P-labeled RNA was incubated either 500 nM (AgvB and GcvB) or 50 nM Hfq (DppA) in the presence of a 50-fold excess of unlabelled RNA. Reactions were incubated at room temperature for 15 min and separated on polyacrylamide gels.

### Competitive Index Experiments

Ten microlitres of each strain was added to 5 ml of LB, 5 ml of MEM-HEPES (supplemented with supplemented with 250 nM Fe(NO_3_)_3_ and 0.1% glucose), or 1 ml of 10% bovine TRM diluted in sterile water. Six batches of mucus were prepared, with a single batch made up of mucus collected from five different animals. Cultures were grown overnight with shaking at 37°C and 10 μl transferred into fresh media of the same for overnight growth. Cultures were serially diluted and plated on LB plates containing kanamycin (both strains) or kanamycin + tetracycline (test strain) and cell numbers enumerated from serial dilutions.

## Accession Numbers

Sequencing and array data is available at GEO under the accession numbers GSE46118 (sequencing) and GSE46113 (array) within the superseries GSE46120.

## Supplemental Information

Document S1. Figures S1–S4, Tables S1–S3, and Supplemental Experimental Procedures

Document S2. Article plus Supplemental Information
