# Supplementary material for: Identification of Bacteriophage-Encoded Anti-sRNAs in Pathogenic Escherichia coli
Source: Mol Cell. 2014 Jul 17;55(2):199–213. doi: 10.1016/j.molcel.2014.05.006 (PMC4104026; doi:10.1016/j.molcel.2014.05.006)
Supplement: Document S2. Article plus Supplemental Information [file mmc2.pdf]

Molecular Cell
Article

CellPress

Identification of Bacteriophage-Encoded Anti-sRNAs in Pathogenic *Escherichia coli*

Jai J. Tree,[1,2,4] Sander Granneman,[1,3] Sean P. McAteer,[2] David Tollervey,[1,*] and David L. Gally[2,*]
[1]Wellcome Trust Centre for Cell Biology, The University of Edinburgh, Edinburgh EH9 3JR, UK
[2]The Roslin Institute and Royal (Dick) School of Veterinary Studies, University of Edinburgh, Edinburgh EH25 9RG, UK
[3]Centre for Synthetic and Systems Biology (SynthSys), University of Edinburgh, Edinburgh EH9 3JD, UK
[4]Present address: Peter Doherty Institute, University of Melbourne, Victoria 3010, Australia
*Correspondence: d.tollervey@ed.ac.uk (D.T.), dgally@ed.ac.uk (D.L.G.)
http://dx.doi.org/10.1016/j.molcel.2014.05.006
This is an open access article under the CC BY license (http://creativecommons.org/licenses/by/3.0/).

SUMMARY

In bacteria, Hfq is a core RNA chaperone that catalyzes the interaction of mRNAs with regulatory small RNAs (sRNAs). To determine in vivo RNA sequence requirements for Hfq interactions, and to study riboregulation in a bacterial pathogen, Hfq was UV crosslinked to RNAs in enterohemorrhagic *Escherichia coli* (EHEC). Hfq bound repeated trinucleotide motifs of A-R-N (A-A/G-any nucleotide) often associated with the Shine-Dalgarno translation initiation sequence in mRNAs. These motifs overlapped or were adjacent to the mRNA sequences bound by sRNAs. In consequence, sRNA-mRNA duplex formation will displace Hfq, promoting recycling. Fifty-five sRNAs were identified within bacteriophage-derived regions of the EHEC genome, including some of the most abundant Hfq-interacting sRNAs. One of these (AgvB) antagonized the function of the core genome regulatory sRNA, GcvB, by mimicking its mRNA substrate sequence. This bacteriophage-encoded "anti-sRNA" provided EHEC with a growth advantage specifically in bovine rectal mucus recovered from its primary colonization site in cattle.

INTRODUCTION

RNA-based regulation (riboregulation) plays a pivotal role in modulating transcript stability and translation efficiency in all domains of life. In bacteria, small regulatory RNAs (sRNAs) have emerged as a major class of regulators of mRNA translation and stability. The canonical pathway for repression of mRNA translation involves an sRNA annealing at or close to the Shine-Dalgarno (SD) ribosome binding site to prevent recognition of the transcript by the 30S ribosomal subunit (Bouvier et al., 2008). sRNA-mRNA duplex formation may be coupled to recruitment of RNase E and lead to accelerated turnover of the transcript (Lalaouna et al., 2013; Pfeiffer et al., 2009). However, a broad range of additional sRNA regulatory mechanisms are being uncovered (Bossi et al., 2012).

sRNA regulation in bacteria is best understood in *Escherichia coli* and *Salmonella* Typhimurium, in which select sRNA-mRNA interactions have been intensely studied. The majority of sRNA-mRNA interactions in these bacteria are mediated by Hfq, a pleiotropic regulator required for posttranscriptional control of bacterial stress responses and for virulence in a range of pathogens (Chao and Vogel, 2010; Papenfort and Vogel, 2010).

Knowledge of how Hfq recognizes RNA targets has largely been derived from in vitro studies using purified Hfq and RNA. Homo-hexamers of Hfq form doughnut-shaped ring structures, with faces defined as "distal" and "proximal." Cocrystallization of Hfq and poly(A) or poly(U) substrates indicated that the distal face can accommodate a repeated trinucleotide motif composed of A-R(A/G)-N(any nucleotide) (Link et al., 2009), and the proximal face binds hexauridine substrates with a preference for interactions with the 3′OH of poly(U) motifs, such as those found in Rho-independent terminators (Otaka et al., 2011; Sauer and Weichenrieder, 2011). A third RNA-binding site, located on the rim of the Hfq hexamer ("lateral" face) is thought to accommodate the body of the sRNA (Ishikawa et al., 2012; Sauer et al., 2012; Zhang et al., 2013). Conserved arginines at the rim are essential for the chaperone activity of Hfq and have been proposed to nucleate helix formation between complementary mRNA-sRNA pairs (Panja et al., 2013). Global analysis of Hfq binding has been carried out in *Salmonella*, greatly expanding our knowledge of target transcripts and sRNAs in this pathogen (Chao et al., 2012; Sittka et al., 2008).

The enteric pathogen enterohemorrhagic *E. coli* (EHEC) has a mosaic genome structure generated by horizontal gene transfer (HGT) into a core genome that is largely conserved in the related but nonpathogenic *E. coli* K12 str. MG1655 (Hayashi et al., 2001). Pathogen-specific virulence factors can be encoded within this acquired DNA, which has led to the concept of "pathogenicity islands." These can be transferred between bacteria following infection with bacteriophages. In addition, lysogenic bacteriophages integrate their prophage genome into that of the recipient bacterium. Over time, these can become cryptic (i.e., unable to produce viable new bacteriophages) due to sequence mutation and loss. EHEC encodes two major virulence factors, both expressed from horizontally acquired regions: Shiga toxins that are responsible for potentially fatal capillary damage within the kidneys and brain (hemolytic uremic syndrome [HUS]) (Tarr et al., 2005) and a type 3 secretion system (T3SS) that is required for colonization of the reservoir host, cattle (Naylor et al., 2005).

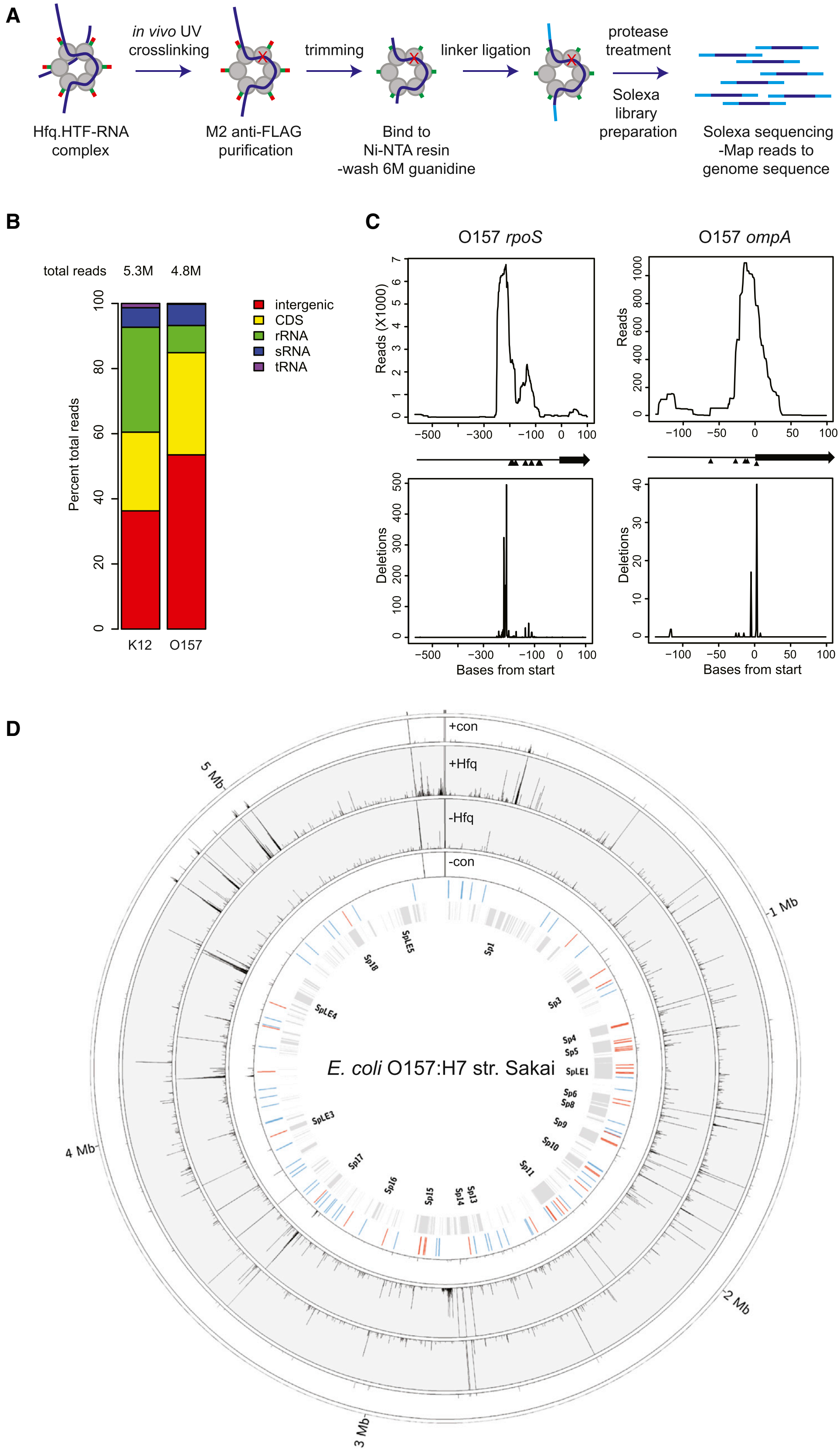

(legend on next page)

Many effector proteins injected into host cells by the T3SS are expressed from cryptic bacteriophage genomes, providing one reason for retention of these regions as part of the EHEC genome.

Here the technique of UV-induced RNA-protein *cr*osslinking and *a*nalysis of *c*DNA by high throughput sequencing (CRAC) was applied to identify transcriptome-wide targets of Hfq binding in EHEC O157:H7.

RESULTS

UV-Crosslinking of Hfq to Target RNAs In Vivo

The chromosomal copy of Hfq was modified by the addition of dual affinity tags in two *E. coli* strains (K12 and EHEC O157) (see Supplementary Information available online). To confirm the functionality of the tagged-Hfq (Hfq-HTF), translational repression of OmpF was measured, since this is known to be Hfq-dependent via targeting of the sRNA MicF (Corcoran et al., 2012). While Hfq-HTF demonstrated mildly reduced activity compared to wild-type Hfq, MicF still repressed OmpF translation by 75%, demonstrating that Hfq-HTF is functional and mediates riboregulation (Figure S1).

The HTF tag allowed highly stringent purification of Hfq from both strains (Figure 1A; Supplemental Information). To assess the crosslinking efficiency, RNA bound to purified, denatured Hfq was 5′ end labeled with ^{32}P (Figures S1A and S1C). Following protease digestion, the recovered RNA was identified by RT-PCR amplification (Figure S1D) and sequencing. Crosslinking was performed independently five times in O157 and twice in K12. Proportions of functional classes of RNA recovered in K12 and O157 are compared in Figure 1B. The most highly enriched protein coding regions (CDS), intergenic regions, and sRNAs are listed in Tables S1A, S1B, and S1C, respectively.

The CRAC data were consistent with interactions established in previous studies on individual RNAs. For example, Hfq crosslinked reads in the *rpoS* mRNA peaked at −215 adjacent to the AAYAA element (−196 to −185, and at −133 adjacent to the U_4 element (−120 to −123) (Figure 1C), in agreement with in vitro binding sites (Moll et al., 2003; Soper and Woodson, 2008). A similar binding pattern was observed for the *rpoS* leader from CRAC analyses in *E. coli* K12 (data not shown). Previous in vitro footprinting of Hfq on *ompA* mRNA demonstrated protection of the SD sequence, the binding site for 30S ribosomal subunits, and the start codon (Moll et al., 2003). In the CRAC data, maximal reads were recovered from the SD at positions −12 to −14 (Figure 1C). The genome-wide Hfq binding profile from a representative data set for O157 is presented in Figure 1D.

Hfq Targeting: Hfq Preferentially Associates with AGR Trimers and Ribosome Binding Sites in mRNAs

The distal surface of Hfq is proposed to bind repeats of ARN, with one trimer bound in a pocket on each monomer (Link et al., 2009). pyMotif from the pyCRAC software package (Webb et al., 2014) was used to identify trimers enriched within Hfq-bound read clusters (Figure 2A). Analysis of the CRAC data sets identified an overrepresented, purine-rich trimer in each data set that would match repeats of AGA or AGG (Figure 2B). These results are consistent with recognition of an ARN trimer by the distal face of Hfq.

The canonical mechanism of negative regulation by Hfq involves promoting seed sequence binding of an sRNA to an mRNA 5′ UTR to preclude 30S ribosomal subunit association with the SD sequence (Bouvier et al., 2008). In line with this mechanism, a sharp spike in reads was observed 13 (±2.1) nt 5′ to the start codon, corresponding to the consensus SD site (Figure 2C). In addition, binding of sRNAs within the first five codons of the coding sequences (CDS) impedes SD recognition, while interactions further 3′ may affect translation by recruiting the RNA degradosome to the transcript (Bouvier et al., 2008; Pfeiffer et al., 2009). To analyze the distrubition of read clusters across all CDSs, we divided each coding sequence into 100 bins and plotted read cluster density (Figure 2D). Around 39% of recovered reads mapped within CDSs and, of these, 82% (32% of total reads) were outside the 5-codon window for SD inhibition. This indicates that targeting the transcript for cleavage may be the mechanism of repression for approximately one third of Hfq-bound mRNAs.

Transcripts that are targeted for degradation in *E.coli* can be oligo(A) tailed by poly(A) polymerase I, providing a single-stranded tail that promotes degradation by 3′→5′ exonucleases (reviewed in Bandyra and Luisi, 2013). Loss of Hfq increases the frequency and length of oligo(A) tails, consistent with functional interactions (Le Derout et al., 2003). Analysis of nonencoded 3′ A tails revealed 5% of sequences crosslinked to Hfq were adenylated, 81% of which carried short oligo(A) tails of 2–6 nt (Figure 2E). This, however, is likely to be an underestimate of the frequency of oligo(A) tails in Hfq-associated RNAs, since these will be detected only if (1) Hfq is bound sufficiently close to the 3′ end of the RNA for their inclusion in short sequence reads, and (2) the nonencoded A sequence is sufficiently short for the remaining sequence to be mapped to the transcriptome.

Hfq Targeting: Hfq Binds Specific Motifs Within or Overlapping mRNA Seed Sequences

The position of Hfq-bound read clusters was examined around established mRNA-sRNA seed sequences. Read clusters were

Figure 1. UV Crosslinking of Hfq-RNA Correlates with In Vitro Footprinting of Hfq to Abundant mRNAs

(A) Workflow for CRAC analysis of Hfq. A detailed protocol is presented in Supplemental Experimental Procedures and Figure S1.

(B) Distribution of Hfq-bound reads between transcript classes in *E. coli* K12 str. MG1655 and *E. coli* O157 str. Sakai. Total reads are indicated above bars.

(C) Sequencing reads recovered from Hfq CRAC that map to *rpoS* or *ompA* mRNAs (top) and deletions recovered within sequencing reads (below). Black arrows between plots indicate the position of coding sequence (arrow) and 5′ UTR (line). Black triangles indicate position of nucleotides protected by Hfq in footprinting experiments in vitro (Moll et al., 2003; Soper and Woodson, 2008).

(D) Transcriptome-wide profiling of Hfq binding sites. Numbers of Hfq-associated reads mapped to the positive strand (+Hfq) and negative strand (−Hfq) are plotted in the gray line plots (y axis maximum 20,000 reads). Control experiments with untagged protein are plotted in the white outer and inner line plots (con±; y axis maximum 10,000 reads). From the inner-most track: text indicates designations for pathogenicity islands, with the position of all pathogenicity islands indicated by the gray boxes in the next track. The positions of sRNAs identified in this study are indicated in red, with previously described sRNAs in blue.

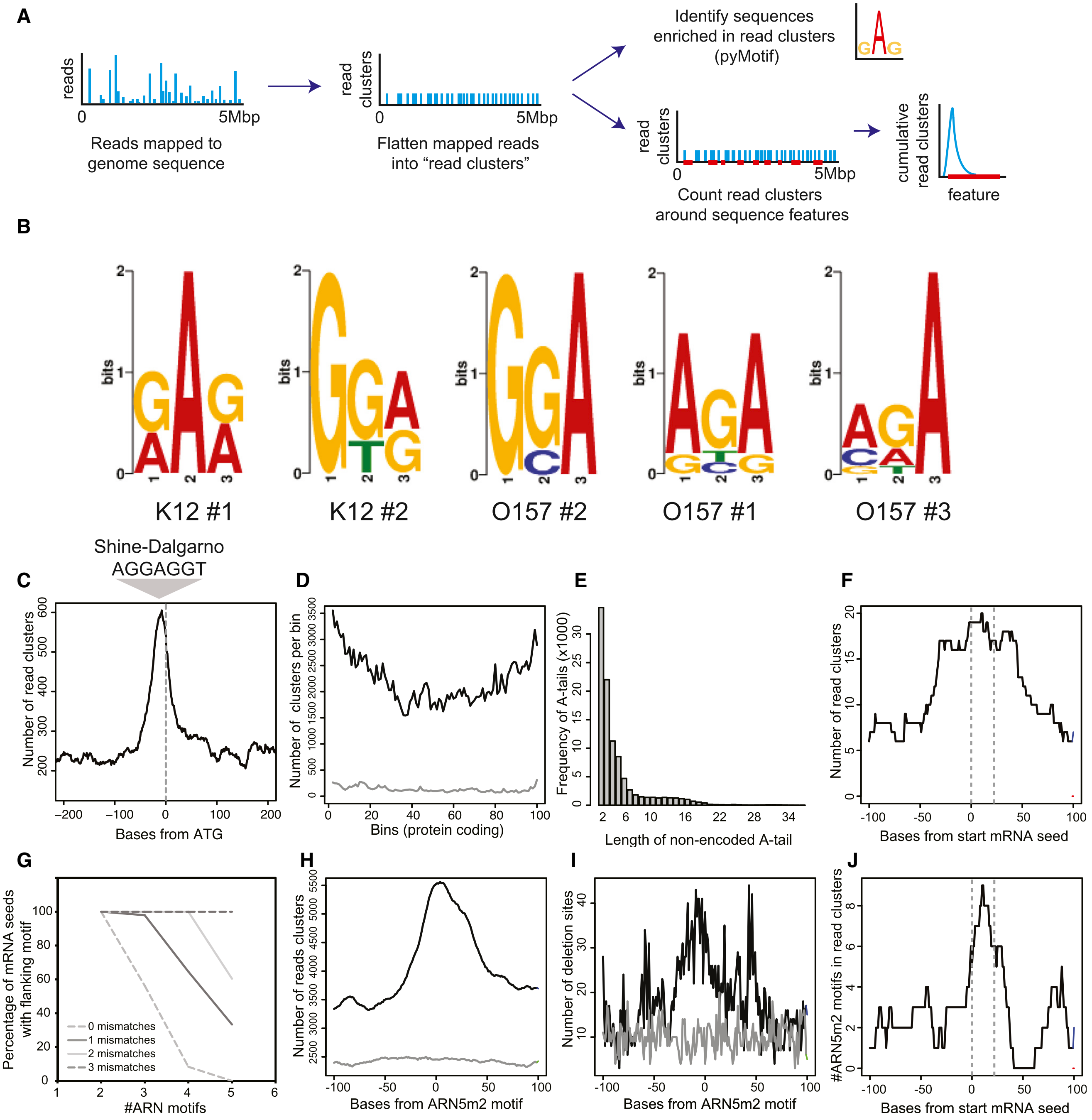

Figure 2. Hfq Binds an ARN Motif Adjacent or Overlapping the mRNA Seed Sequence

(A) Workflow for analysis of Hfq crosslinked reads. Mapped reads were flattened into read clusters to prevent bias toward highly enriched sites. Read clusters are analyzed for enriched motifs (as in [B]) or their culmulative distrubution around sequence features such as CDS and mRNA seed regions (as in [C]–[J]).

(B) pyMotif from the pyCRAC software package was used to identify trimers that were enriched within RNAs crosslinked to Hfq in five independent experiments. Hfq was crosslinked in either nonpathogenic *E. coli* K12 str. MG1655 (K12) or enterohemorhaggic *E. coli* O157:H7 str. Sakai (O157). All five logos fit either a repeated AGG or AGA sequence (indicated below).

(C) Cumulative Hfq-bound read clusters are plotted relative to the start codon (indicated by gray dashed line). The sequence and approximate position of the Shine-Dalgarno sequence is indicated above.

(D) Cumulative Hfq binding within coding sequences. CDS were divided into 100 bins and scored for overlapping read clusters. The cumulative score (genome wide) for each bin is indicated in black and the cumulative score for shuffled CDS coordinates in gray (CDS were assigned random positions within the genome).

(E) Frequency of non-genomically encoded oligo(A)-tail length recovered from Hfq-bound reads.

(F) Cumulative Hfq-bound read clusters within 100 nt of experimentally verified mRNA seed sequences. Grey dashed lines indicate the position and width for the average mRNA seed.

(legend continued on next page)

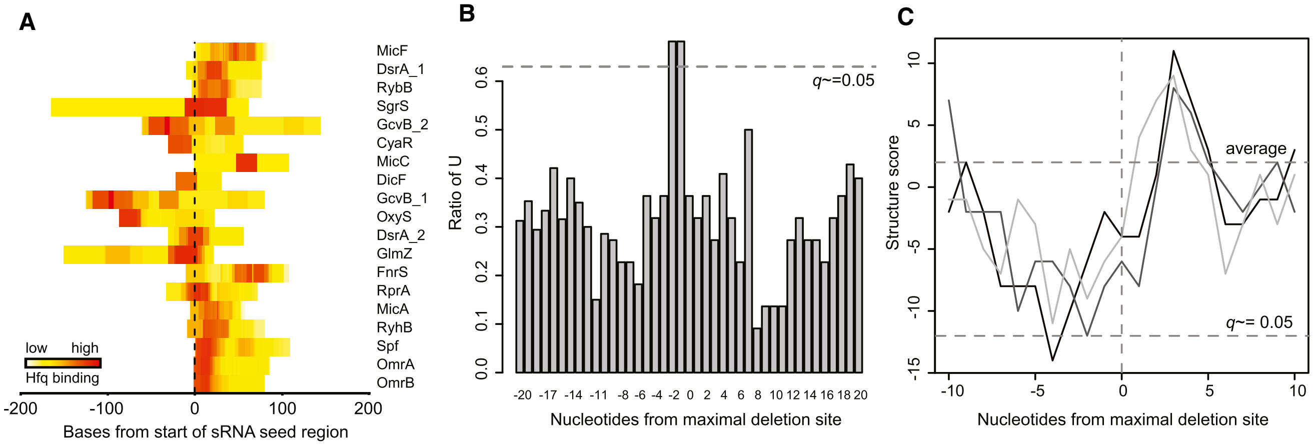

Figure 3. Hfq Binds Single-Stranded, U-Rich Sequences in sRNAs

(A) Hfq binding relative to sRNA seed sequences. Small RNAs (indicated right) are aligned to the start of their respective seed regions (dashed line). Each heatmap indicates Hfq binding along the sRNA.

(B) A 2U sequence is enriched 5′ of the site of maximal deletions (indicating direct Hfq contact). Positions relative to the site of maximal deletions within 20 Hfq-dependant sRNAs were scored for frequency of a uridine nucleotide. The probability of randomly enriching U at a given position (FDR) is given by the gray dashed line ($q \approx 0.05$).

(C) Hfq is crosslinked to single-stranded nucleotides within sRNAs. The secondary structure of 20 Hfq-dependent sRNAs was predicted using the UNAfold software package and nucleotides surrounding the site of maximal deletions were scored as base paired (+1) or unpaired (−1). The cumulative score for nucleotides from 20 Hfq-dependent sRNAs are plotted against their position relative to the maximal crosslinking site for three independent experiments. False discovery rate is given by the gray dashed line ($q \approx 0.05$).

found to be enriched directly over mRNA seed sequences for 46 experimentally verified interactions (Figure 2F, average mRNA seed size indicated by dashed gray lines) (Beisel et al., 2012; Cao et al., 2010; Corcoran et al., 2012; Sharma et al., 2011) with no clear bias for association 5′ or 3′ to the seed sequence. These results indicated that Hfq binds mRNA targets directly at, or immediately adjacent to, the mRNA seed. We next determined whether mRNA seeds were also associated with an ARN motif. As most Hfq binding sites were identified within 100 nt of the mRNA seed (Figure 2F), we assessed whether repeated ARN motifs were present within this region. A strict ARN4 or ARN3 repeat was present within 100 nt of 3/46 or 23/46 mRNA seeds, respectively (Figure 2G). Allowing a single mismatch in the ARN4 motif (ARN4m1) allowed matches to be found flanking 30/46 mRNA seeds, whereas allowing two mismatches within an ARN5 motif (ARN5m2) matched 31/46 seed sites. Mapping Hfq read density around ARN5m2 motifs transcriptome-wide confirmed strong enrichment relative to random genomic positions (Figures 2H and 2I). Plotting Hfq-bound ARN5m2 motifs relative to mRNA seed sequences showed a clear peak within the seed sequence, confirming that the motif for distal-side binding often overlaps with the mRNA seed (Figure 2J with sequences presented in Figure S2).

We conclude that most sites of Hfq-associated sRNA-mRNA basepairing overlap or are closely associated with a repeated ARN motif in the mRNA, which binds the distal face of Hfq.

Hfq Targeting: Hfq Binds U-Rich ssRNA Sequences in sRNAs

The proximal face of Hfq is reported to bind single-stranded A/U rich sequences, which are present in many sRNAs (Ishikawa et al., 2012; Otaka et al., 2011; Sauer and Weichenrieder, 2011; Schumacher et al., 2002). The locations of Hfq-bound read clusters were assessed relative to 21 experimentally verified sRNA seed regions (from 46 seed sequences, overlapping seeds were condensed into a single seed "region"). The Hfq binding peak overlapped the known sRNA seed sequence in a majority of sRNAs (Figure 3A). To examine the sequence and structural requirements for Hfq binding within sRNAs, we examined verified Hfq-dependant sRNAs (22 sRNAs extracted from sRNATarbase) for common features associated with the location of maximum point deletions from the CRAC analysis, as these signify sites of direct Hfq contact. Analysis of nucleotide frequencies revealed strong enrichment for a U-U dinucleotide immediately 5′ to the crosslinking site (Figure 3B). Secondary structure prediction showed that the region 5′ to the crosslinking

(G) Percent of mRNA seeds with ARN motifs within 100 nt allowing mismatched postions. The x axis represents the number of ARN repeats within a motif, and the y axis represents the percentage of mRNA seeds with that motif within 100 nt. The percentage of mRNA seeds with a flanking ARN motif is plotted for zero to three mismatched postions.

(H) Transcriptome-wide cumulative count of Hfq bound read clusters at ARN5m2 motifs (black) and control shuffled ARN5m2 coordinates (gray).

(I) Transcriptome-wide cumulative count of deletions in Hfq-bound read clusters at ARN5m2 motifs (indicating direct Hfq contact; black) and control shuffled ARN5m2 coordinates (gray).

(J) Position of ARN5m2 motifs within Hfq bound reads at experimentally verified mRNA seed sequences (see also Figure S2 for sequences). Grey dashed lines indicate the position and average width of mRNA seed sequences.

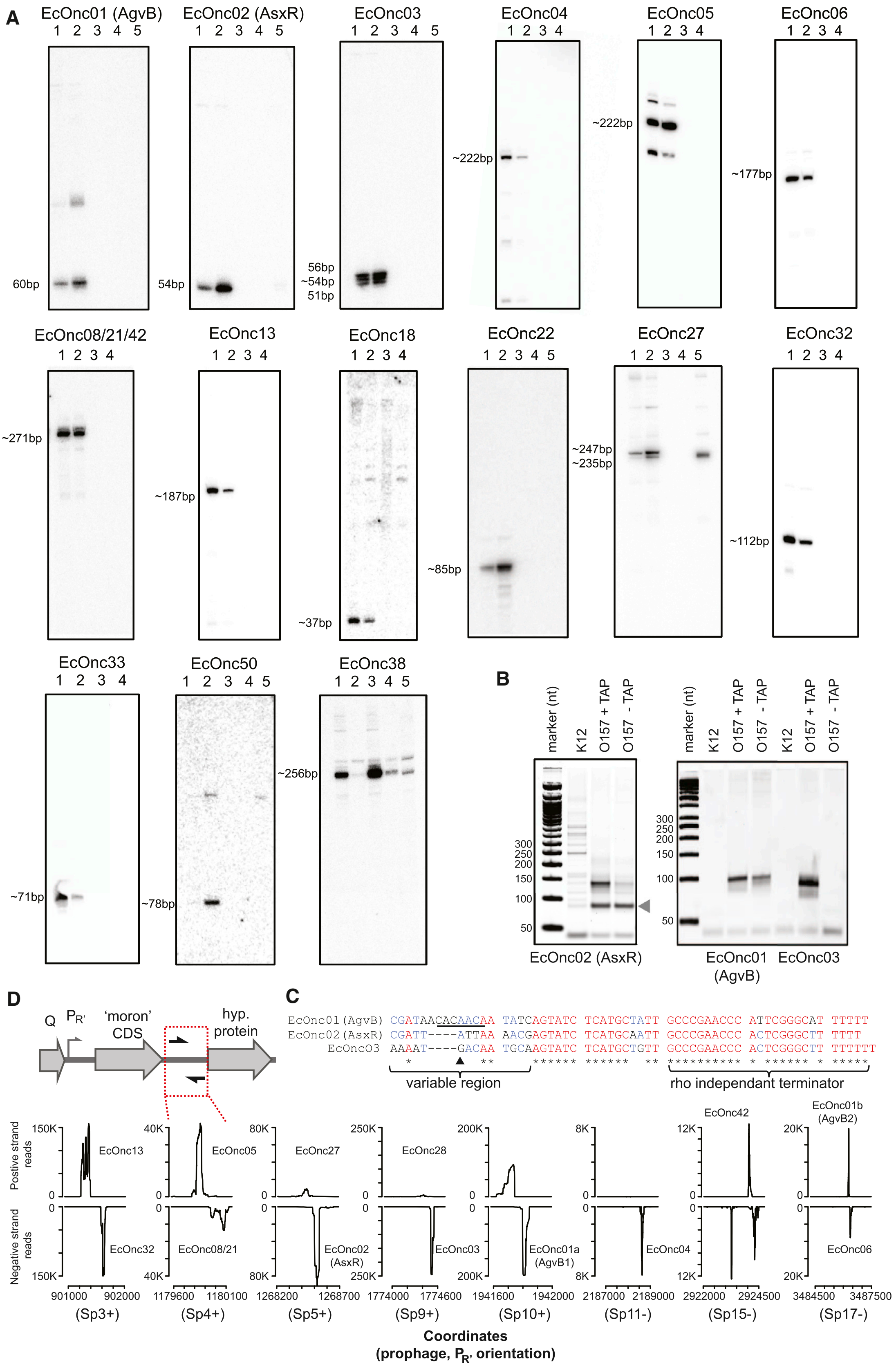

(legend on next page)

site was also significantly ($q < 0.05$) enriched for unpaired nucleotides (low values in Figure 3C). In contrast, the region 3′ to the Hfq binding site showed enrichment for basepaired nucleotides. Peaks of Hfq binding were not recovered at Rho-independent terminators. However, the 3′ OH of the U_6 sequence is in direct contact with Hfq, and UV crosslinking here may inhibit 3′ linker ligation, potentially biasing our results against recovery of poly(U) tails.

We propose that the consensus Hfq binding site on many sRNAs includes a U-U dinucleotide associated with an unpaired region.

sRNAs Are Encoded within Pathogenicity Islands of EHEC O157

Around 25% of the O157 chromosome is comprised of bacteriophage-derived pathogenicity islands, and 27% of total Hfq-bound reads were mapped to these regions.

To locate noncoding RNAs, we filtered our data for reproducible Hfq targets located antisense to, or >100 bp away from, coding regions (see Supplemental Experimental Procedures). This analysis identified 63 unannotated, potential noncoding sRNAs within the O157 transcriptome. Eight of these were encoded within the core genome and 55 within pathogenicity islands (Figure 1D, genomic positions of predicted sRNAs are indicated in red; Table S2). One sRNA expressed from the pathogenicity islands of EHEC has been described, Esr41 (Sudo et al., 2014). Pathogenicity islands are enriched for predicted sRNA genes relative to the core genome, with an average of 39 sRNA per Mb of accessory genome and 23 sRNAs (Keseler et al., 2013; Raghavan et al., 2011) per Mb of core genome.

Rho-independent termination is a common feature for many sRNAs and was shown to contribute to Hfq binding in some cases (Otaka et al., 2011; Sauer and Weichenrieder, 2011). The RNAmotif descriptor for *E. coli* Rho-independent terminators was used to identify terminator loops within 200 nt of the 3′ edge of the Hfq binding site (see Supplemental Experimental Procedures). Thirty-one of the sRNAs identified in this study were predicted to carry Rho-independent terminators (Table S2).

Northern blot analysis confirmed the expression of 17 predicted sRNAs (from 18 tested), with sizes ranging from approximately 37–354 nt (Figure 4A). sRNAs are commonly destabilized in the absence of Hfq, and eight of these confirmed sRNAs (the four most abundant, three encoded on StxΦ, and a core encoded sRNA) were characterized in a Δ*hfq* background. Six sRNAs were destabilized by loss of Hfq, one sRNA was approximately 4 nt shorter, and one sRNA was stable (Figure 4A; Table S2). The abundance of one of the eight unannotated core genome sRNAs, EcOnc38, was low in LB medium but higher in MEM-HEPES, suggesting that it may have escaped previous detection due to poor expression.

Prophages Encode a Class of Unusually Short sRNA

The unannotated sRNAs most frequently recovered by Hfq CRAC in *E. coli* O157 were EcOnc01, EcOnc02, and EcOnc03. The 5′ ends of these transcripts were mapped to identify primary transcription start sites (Figures 4B and 4C), confirming they encode unusually short sRNAs between 51 and 60 nt. For EcOnc03, heterogeneous triphosphorylated 5′ ends were detected (between the 5′ end and black arrow in Figure 4C), consistent with northern blot detection of three distinct RNA species. These, and several other sRNAs, were expressed from genes located at conserved locations within lambdoid prophages. The sequence downstream of the bacteriophage Q antiterminated $P_{R'}$ promoter tolerates DNA insertions termed "morons" (more DNA or more "ome") (Juhala et al., 2000) and carried convergent sRNA genes. This is exemplified by the bacteriophage Sp5 that encodes Shiga toxin 2 at a moron insertion site (Figure 4D; see plot for Sp5), where convergent sRNAs (EcOnc02 and EcOnc27) are encoded 3′ of the *stx2B* gene. A similar gene organization was seen for other lambdoid prophages Sp3, Sp4, Sp9, Sp10, Sp11, Sp15, and Sp17 (Figure 4D). Many of the sRNAs encoded at these positions fall into related groups but are not identical. The four most abundant sRNAs, EcOnc01a, EcOnc01b, EcOnc02, and EcOnc03 (encoded within Sp10, Sp17, Sp5, and Sp9, respectively) share highly conserved 3′ regions of ~42 nt but have variable 5′ regions of 14–18 nt (Figure 4C).

EcOnc02 Is Encoded within the Stx2Φ and Derepresses a Heme Oxygenase

The gene encoding EcOnc02 is located 282 bp 3′ and antisense to *stx2AB*, which encodes the major virulence factor Shiga toxin 2. Analyses of EcOnc02 and EcOnc01 (below) indicate that these represent a class of "anti-sRNAs," and we have renamed EcOnc02 as AsxR. To identify functional targets, AsxR was transiently overexpressed (10 min pulse) and changes in mRNA abundance were monitored using oligonucleotide microarrays. To identify directly regulated targets, transcripts showing altered abundance were screened for the presence of Hfq binding sites within 200 nt of the CDS (Figure S3A). *chuS* and *chuW* were each found to be more abundant after a 10 min pulse of AsxR transcription and associated with Hfq by CRAC analysis.

Figure 4. Identification of Prophage-Encoded sRNAs in *E. coli* O157

(A) Northern blot analysis of predicted sRNAs (also see Table S2) in *E. coli* O157:H7 str. Sakai (O157) and nonpathogenic *E. coli* K12 (K12) cultured under virulence inducing conditions (MEM-HEPES) and in LB broth. Lane 1: O157 grown in MEM-HEPES; lane 2: O157 grown in LB; lane 3: K12 grown in MEM-HEPES; lane 4: K12 grown in LB; lane 5 (where applicable): O157Δ*hfq* grown in LB. Approximate size of RNAs indicated left of blot

(B) 5′ RLM-RACE with and without tobacco acid pyrophophatase (TAP) treatment of EcOnc01–EcOnc03. Grey arrow indicates a primer dimer.

(C) Prophage encode convergent sRNAs within the "moron" insertion site at $P_{R'}$. (Top) Graphical representation of gene organization at the moron CDS insertion site showing the phage regulator, antiterminator Q CDS, and promoter $P_{R'}$. Moron CDSs are inserted downstream of $P_{R'}$, and convergent sRNAs are encoded between the moron CDS and a conserved hypothetical phage ORF. (Bottom) Hfq-bound reads are plotted for the intergenic region between moron CDS and downstream hypothetical ORF (indicated by red box above) for prophages encoding convergent sRNAs. Prophage designation and strand encoding $P_{R'}$ are given in brackets. Peaks that have been assigned to predicted sRNA are indicated.

(D) Alignment of EcOnc01–3. Underlined sequence in EcOnc01 corresponds to the GcvB targeting consensus. The black triangle indicates the shortest alternate 5′ triphosphate end detected by 5′RLM-RACE in EcOnc03.

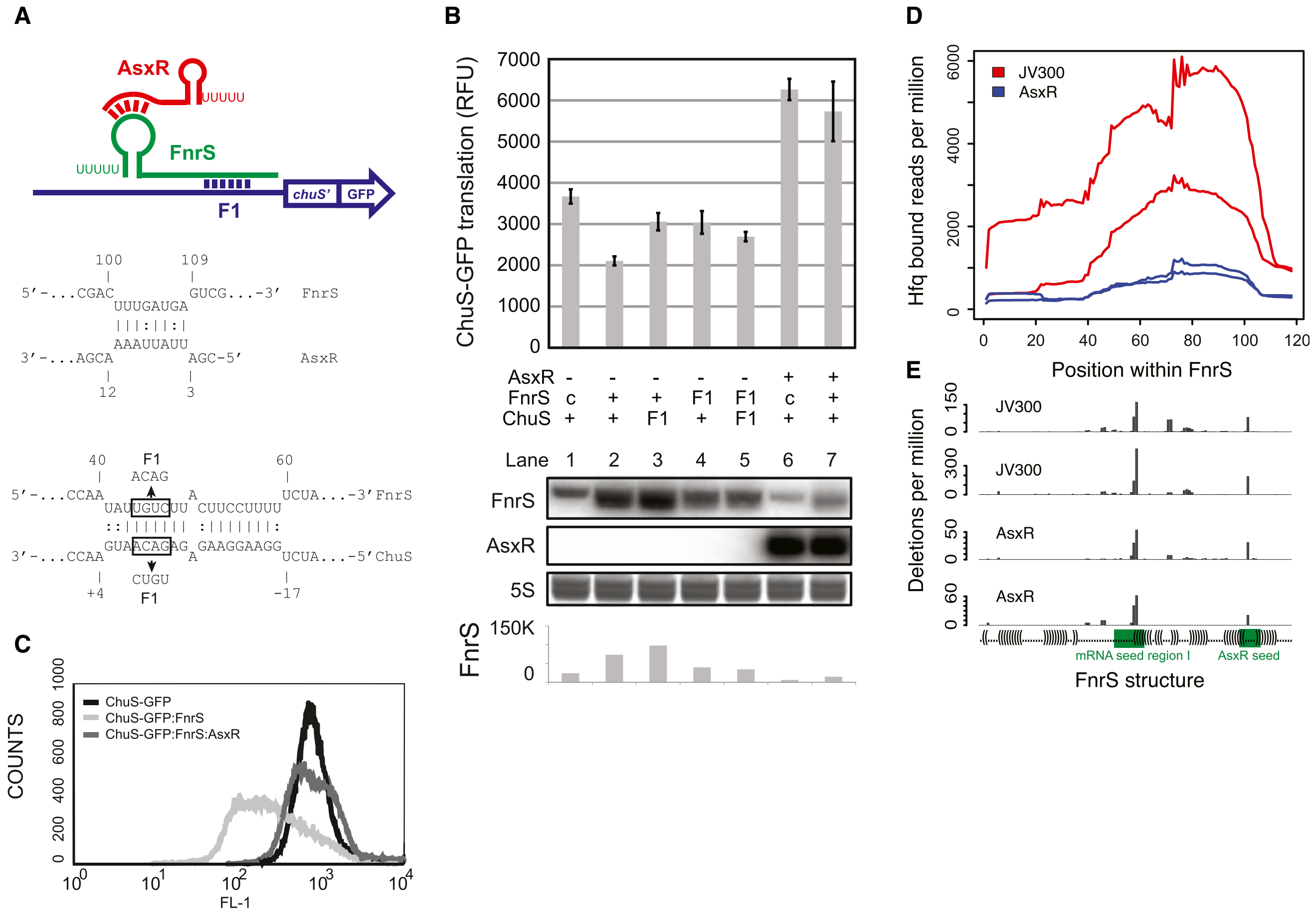

Figure 5. The Shiga Toxin 2 Locus Encodes an Anti-sRNA that Enhances Expression of the Heme Oxygenase ChuS

(A) (Top) Graphical representation of interactions between AsxR, FnrS, and the *chuS* mRNA. F1 indicates the positions of the complementary mutation. (Bottom) Predicted base paring (IntalRNA software) between AsxR and FnrS, and FnrS and the *chuS* transcript. Boxes and arrows indicate sequence changes that were introduced into F1 mutants.

(B) (Upper panel) Fluorescence of the 3′ chuA→5′ chuS *chuS*-GFP translational fusion was monitored in the presence of FnrS, AsxR, and appropriate point mutants (indicated below bar chart; basal levels of chromosomal FnrS are indicated by "c"). (Lower panel) Northern blot analysis of FnrS and AsxR (indicated). SYBR-green-stained 5S rRNA (5S) is included as a loading control. (Bottom) Quantification of FnrS northern blots by densitometry. Error bars indicate SEM.

(C) Flow cytometry quantification of fluorescence from cells expressing *chuS*-GFP alone, with FnrS, or with both FnrS and AsxR.

(D) AsxR reduces Hfq-bound FnrS. The *chuS*-GFP fusion and FnrS were constitutively expressed in *E. coli* MG1655 *hfq*-HTF with AsxR (blue) or the control plasmid pJV300 (red) and CRAC performed on these strains. Replicate data sets are plotted as reads per million across FnrS.

(E) Hfq binds to both seed and 3′ loop regions of FnrS. Deletions per million Hfq-bound reads are plotted relative to secondary structure of FnrS. Major deletion sites are located within the mRNA seed region I (green) and the AsxR seed region (green) within the terminator loop. See also Figure S3.

The *chuS* gene encodes a heme oxygenase and lies downstream of *chuA,* which encodes a heme outer-membrane receptor. The predicted 5′ UTR and *chuAS* region was cloned into the GFP fusion vector pXG10-SF to monitor translation. Translation of *chuAS* was increased 2.5-fold in the presence of AsxR, consistent with our microarray analysis (Figures S3B and S3C; *chuAS* samples). In order to identify the minimal sequence requirements for increased translation of *chuS*, regions of the *chuAS* transcript were subcloned into the GFP fusion vector pXG30-SF that provides an upstream coding sequence (*lacZ′*) to allow translational coupling (Corcoran et al., 2012). A 155 nt transcript, extending from the *chuA* stop codon to +66 nt of *chuS*, had 2.3-fold more translation in the presence of AsxR (Figures S3B and S3C). This region lacks complementarity to AsxR, suggesting that AsxR might function indirectly via a regulator that binds directly to this 155 nt fragment. IntaRNA software was used to screen for putative interactions with known sRNA regulators and revealed extensive complementarity between the sRNAs RyhB and FnrS and the SD site of *chuS*. Constitutive expression of RyhB or FnrS repressed translation of the ChuS fusion reporter (data not shown). AsxR lacks clear complementarity to RyhB, but its 5′ end could potentially basepair to the single-stranded loop of the Rho-independent terminator of FnrS (Figure 5A). Furthermore, an interaction between AsxR and FnrS is consistent with our FnrS-Hfq CRAC data, which showed two prominent peaks of deletions within FnrS; one

maps to the known seed site for mRNA binding (Durand and Storz, 2010) and another within the terminator stem loop.

A three-plasmid system was used to monitor the roles of FnrS and AsxR in controlling translation of a construct containing nts −112 to +66 relative to the ChuS start codon fused to GFP (Figure 5B). Translation of ChuS was repressed by expression of FnrS (Figure 5B, lanes 1 and 2), which was partially relieved by mutation (F1) of either FnrS or ChuS (Figure 5B, lanes 3 and 4). Coexpression of AsxR relieved the repression of ChuS translation by FnrS (Figure 5B, upper panel, lane 7), and this was confirmed using flow cytometry (Figure 5C). Basal translation of the ChuS-GFP fusion was increased in the presence of AsxR alone, as seen for the vector expressing the entire *chuAS* region (Figure 5B, lane 6), indicating that ChuS translation is repressed by endogenous FnrS.

Northern analysis showed that the level of FnrS is reduced in the presence of AsxR, consistent with AsxR binding to the terminator stem, which is required for stability of the 3′ end of FnrS (Figure 5B, lower panel, compare lanes 2 and 7) (Blum et al., 1999; Cisneros et al., 1996; Figueroa-Bossi et al., 2009). Compensatory 3 nt mutations (S1) were introduced into the FnrS 3′ stem loop and the 5′ region of AsxR, but both S1 mutations were strongly destabilizing. We additionally performed Hfq-CRAC analysis on the three-plasmid system using the *E. coli* str. MG1655 *hfq*-HTF background (lacking both *chuS* and *asxR*). ChuS-GFP and FnrS were constitutively expressed in the presence of AsxR or the control plasmid pJV300. Consistent with our northern analysis, the association of FnrS with Hfq was strongly reduced in the presence of AsxR (Figure 5D). Deletions identify precise Hfq binding sites and mapped to both the mRNA seed region I and to the single-stranded loop of the Rho-indpendant terminator (Figure 5E). These interactions were detected in the presence or absence of AsxR, indicating that Hfq contacts the terminator loop under both conditions. We conclude AsxR acts to increase expression of the ChuS heme oxidase via destabilization of FnrS.

EcOnc01 Functions as an Anti-sRNA that Antagonises GcvB

We noted that the 5′ variable domains of the most abundant unannotated sRNAs recovered, EcOnc01a and EcOnc01b, contain the consensus target sequence for the R1 seed sequence of the core genome-encoded sRNA, GcvB (CACAACA; underlined Figure 4C) (Sharma et al., 2011). In silico predictions support the potential for EcOnc01 to bind the R1 seed sequence of GcvB (Figure 6A), and we have renamed EcOnc01 *a*nti-sRNA for *GcvB* (AgvB).

To test for interactions between AgvB and GcvB, we used a GFP translational fusion to the dipeptide transporter DppA mRNA from *Salmonella* Typhimurium, as this is known to be repressed by GcvB (Sharma et al., 2007; Urbanowski et al., 2000). Our three-plasmid system was used to express AgvB, GcvB, and DppA in *E. coli* Top10F′, which we found to have an 8 nt deletion in the R1 seed sequence of the endogenous copy of GcvB, inactivating the chromosomal copy of the GcvB R1 seed. Overexpression of GcvB was found to be toxic and induction from $P_{LtetO-1}$ was reduced until growth was restored. Expression of GcvB inhibited translation of DppA mRNA (Figure 6B, lanes 1 and 2), whereas coexpression of AgvB with GcvB restored DppA translation (Figure 6B, upper panel, lane 4). These results were confirmed by flow cytometry (Figure 6C). AgvB had no significant effect on DppA expression in the absence of GcvB (Figure 6B, lanes 1 and 3). To determine whether AgvB interacts directly with GcvB, base changes (designated G1) were introduced into AgvB, GcvB, and DppA mRNA (Figure 6A). The G1 mutation in DppA was insufficient to destabilize the GcvB-DppA interaction, as DppA-G1 was repressed by GcvB. However, direct interaction between GcvB and DppA at the R1 seed has been rigorously demonstrated using a GcvB ΔR1 mutant and footprinting, indicating that the 4 nt G1 mutation is insufficient to destabilize the long R1 pairing (Sharma et al., 2007). Similarly, a G1 mutation in GcvB was insufficient to relieve DppA repression, although repression by GcvB-G1 was slightly reduced likely due to mutation of an ACA-motif required for optimal translation (Figure 6B, lanes 5–7) (Yang et al., 2014). However, the G1 mutation within the 5′ variable region of AgvB was sufficient to prevent the derepression of DppA, evident when comparing lanes 5, 8, and 10 (Figure 6B). Depression by the modified anti-sRNA (AgvB-G1) was restored when AgvB-G1 was expressed in the context of GcvB-G1 and DppA-G1 (Figure 6B, comparing lanes 7, 9, and 11). Northern analysis did not indicate a significant reduction in the level of GcvB following coexpression of AgvB (Figure 6A, lower panel). We conclude that AgvB antagonizes GcvB function by hybridizing to the seed region and blocking its interactions with target mRNAs.

To verify the anti-GcvB function of AgvB in the pathogenic background, we deleted both copies (EcOnc01a and EcOnc01b) from *E. coli* O157:H7 str. Sakai. The translation efficiency of DppA mRNA was measured using a constitutively transcribed GFP fusion to the 5′ UTR and the first 10 codons of DppA from *E. coli* O157:H7. Deletion of both *agvB1* and *agvB2* resulted in a 32% reduction in translation of DppA, and complementation of the mutant using constitutively transcribed AgvB restored translation by 24% relative to the mutant (Figure 6D). These results demonstrate that AgvB modulates translation of DppA in pathogenic *E. coli* O157:H7.

E. coli O157:H7 colonizes the final few centimeters of the bovine gastrointestinal tract, with the majority of bacteria multiplying in the terminal rectal mucus (TRM) (Naylor et al., 2003; Tildesley et al., 2012). As such, TRM recovered from this site can be used as a relevant growth medium in place of in vivo experiments in cattle. To investigate the potential benefit of regulation by AgvB, competitive index experiments were carried out between the WT strain and the double deletion, Δ*agvB1* Δ*agvB2*, in standard laboratory media (LB and MEM-HEPES) and in TRM. The double deletion of AgvB did not significantly affect growth in the two laboratory media, whereas loss of the sRNA strongly reduced the competitiveness of the strain in TRM (Figure 6E). This result was confirmed by chromosomal complementation of *agvB1* into the double deletion strain followed by competition of the complement against the double deletion strain in TRM. The single complement successfully outcompeted the double mutant (Figure 6E), and we conclude that the pathogenicity island-associated sRNA AgvB aids growth within its animal host reservoir at the specialized site colonized by this pathogen.

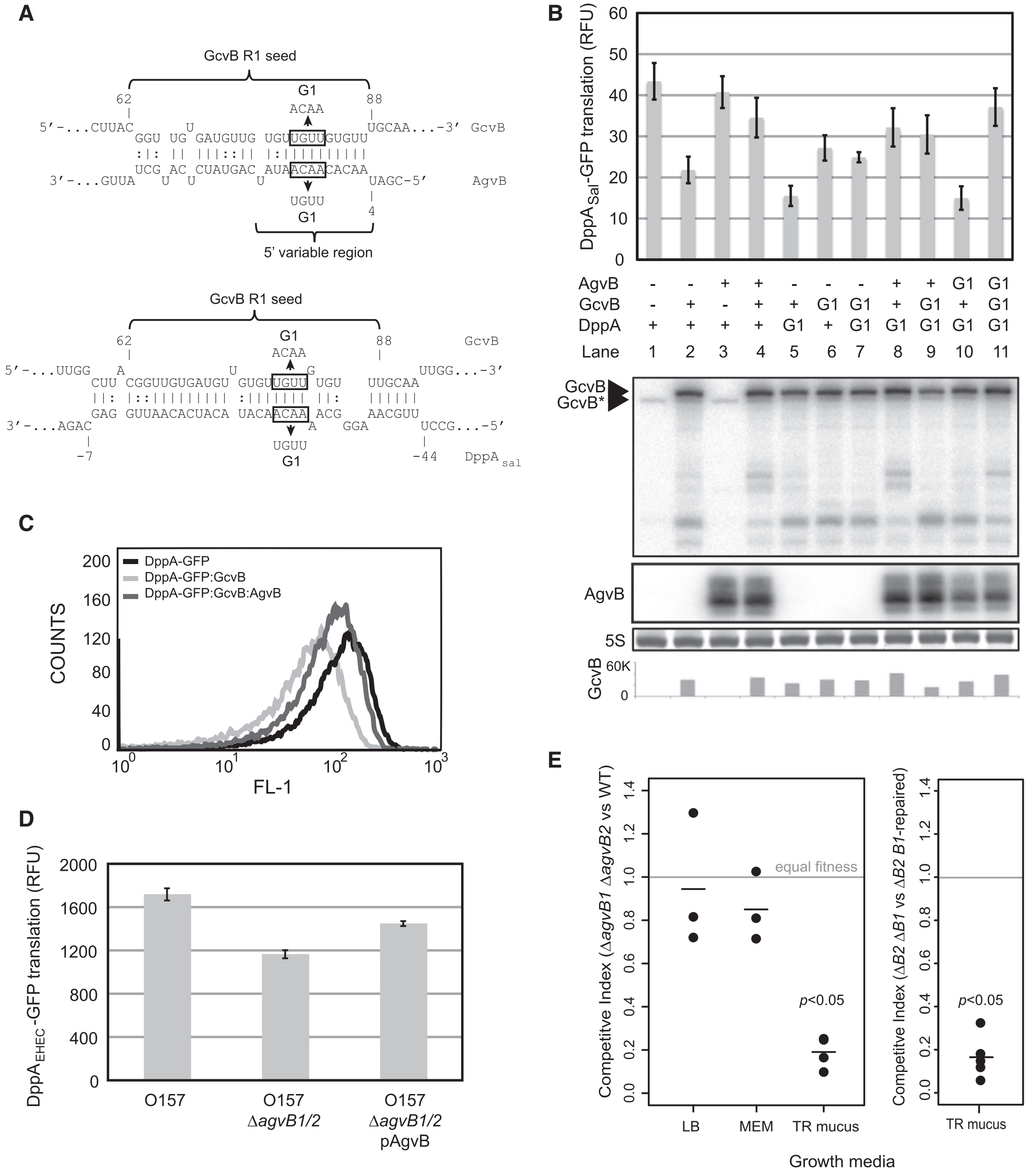

Figure 6. EcOnc01 (AgvB) Acts as an "Anti-sRNA" to Inhibit GcvB Repression

(A) Interactions between GcvB and AgvB (top) and GcvB and DppA$_{Sal}$ (bottom) were predicted using IntaRNA software. The R1 seed sequence of GcvB is indicated in braces, and sequences that were introduced into G1 mutants are indicated within boxes.

(B) Fluorescence of DppA$_{sal}$-GFP was used to monitor GcvB activity in the presence of AgvB, GcvB, and G1 mutants. Genotypes for each reading are indicated below. (Below: GcvB and AgvB) Northern analysis of GcvB and AgvB, respectively. GcvB* indicates the endogenous copy of GcvB, which carries an 8 nt deletion in the R1 seed region. SYBR-green-stained 5S rRNA (5S) is shown as a loading control for GcvB and AgvB northern blots. The bottom panel shows quantification of the exogenous copy of GcvB by densitometry. Error bars indicate SEM.

(C) Flow cytometry quantification of fluorescence from individual cells expressing DppA$_{sal}$-GFP alone, with GcvB, or with both GcvB and AgvB.

(D) Fluorescence of DppA$_{EHEC}$-GFP was used to monitor translation efficiency of DppA in *E. coli* O157:H7, Δ*agvB1* Δ*agvB2*, and the complemented strain Δ*agvB1* Δ*agvB2* pZE12::EcOnc01 (pAgvB).

(E) The left-hand panel shows the competitive indices of *E. coli* O157 Δ*agvB1* Δ*agvB2* against the parent stain (Sakai) grown in LB media (n = 3), MEM-HEPES media (MEM, n = 3), and terminal rectal mucus (TR mucus, n = 4). The right-hand panel shows the competitive indices for the double mutant *E. coli* O157 Δ*agvB1* Δ*agvB2* against the same strain complemented on the chromosome with *agvB1* (TR mucus, n = 5). A competitve index of 1 indicates no fitness difference; <1 indicates a fitness disadvantage.

AgvB Interacts with the mRNA Binding Face of Hfq and Forms a Stable Duplex with GcvB

To understand the mechanism of AgvB-mediated translation derepression, we characterized the interactions between Hfq, AgvB, GcvB, and DppA mRNA. Gel mobility shift analyses of complexes formed in vitro demonstrated that AgvB and GcvB bound Hfq with a comparable affinity (Figures 7A and 7B). Strikingly, the 5′ 166 nt of DppA mRNA bound Hfq with around 10-fold higher affinity (Figure 7C). We then used pairwise competition experiments to characterize interactions with Hfq (Figures 7D and 7E). Addition of excess GcvB to Hfq-DppA binding reactions shifted the labeled DppA complex into higher molecular weight ternary complexes (Figure 7F, lane 4, labeled "H•G•D"), and this was also observed for labeled GcvB in the presence of excess DppA (Figure 7E, lane 5, H•G•D). Excess AgvB competed labeled DppA from Hfq, despite the higher apparent affinity of DppA binding (Figure 7F, lane 3), and DppA was able to compete labeled AgvB from Hfq (Figure 7D, lane 5). This strongly indicates that DppA and AgvB bind the same site on Hfq. We had observed that ARN4m1 or ARN5m2 motifs were present in a majority of bone fide Hfq distal face binding sites (Figure 2G) and also identified ARN4m1 and ARN5m2 motifs within the 5′ variable region of AgvB and AsxR (Figure S4). These results indicate that AgvB and DppA both interact with the distal RNA binding site of Hfq, potentially facilitating annealing with complementary RNAs bound to the proximal face. The addition of GcvB to labeled AgvB binding reactions did not compete AgvB into free RNA but shifted AgvB into a faster migrating complex (Figure 7D, lane 4, and 7E, lane 3, complex A•G) that was detected with both labeled GcvB and AgvB, but lacked detectable Hfq (western blots in Figures 7D and 7E, right panel). The most likely composition of the faster migrating band is a stable AgvB-GcvB duplex. A similar duplex was not formed with excess of an sRNA that does not have complementary to AgvB, FnrS (Figure 7D, lane 7).

We conclude that AgvB and DppA compete for binding to the distal face of Hfq, whereas a stable duplex is formed between the sRNA, GcvB, and its anti-sRNA, AgvB.

DISCUSSION

In mammalian cells, viruses use miRNAs and other RNAs to modulate the host miRNA population. The data presented here demonstrate that bacteriophages and bacteriophage-derived pathogenicity islands express sRNAs that modulate the activities of bacterial host sRNAs. We predicted that these anti-sRNAs alter cell metabolism to favor bacterial colonization of specific host or environmental niches and confirmed this for AgvB.

The majority of Hfq-associated mRNA reads were crosslinked outside of protein coding sequences with a sharp spike in binding at the SD site, consistent with occlusion of the SD by sRNAs. The SD site has a purine-rich motif with consensus AGGAGGT, matching the most overrepresented Hfq-binding trimer in vivo (AGR). Hfq bound read clusters were also enriched at U-rich motifs in sRNAs and at experimentally verified mRNA-sRNA seed interactions on both the mRNAs and sRNA (Figure 3A). The majority of seed-binding sites in mRNAs were also associated with multiple ARN motifs, the consensus motif for binding the Hfq distal face. Hfq binds single-stranded RNA, suggesting that binding of Hfq to the mRNA seed region is in competition with duplex formation between the sRNA and mRNA. Such competition would ensure a minimum free energy threshold for hybridization and provides a simple mechanism allowing Hfq to add stringency to the limited sequence requirements for base pairing between sRNAs and mRNAs. Since the Hfq distal-side binding motif in the mRNA seed is sequestered in sRNA-mRNA duplexes, target acquisition by sRNAs would lead to rapid dissociation of Hfq from the ternary complex, as previously observed (Fender et al., 2010; Hopkins et al., 2011; Lease and Woodson, 2004; Updegrove et al., 2008). This would also prevent duplexed mRNAs from reassociating with Hfq and competing with unpaired mRNAs.

Hfq and Xenogenic sRNA

Escherichia coli O157:H7 str. Sakai shares a common "core" genome of 4.1 Mb with the commensal isolate *E. coli* K12 (Hayashi et al., 2001). The majority of pathogenicity determinants are encoded within an extra 1.4 Mb of horizontally acquired DNA elements, including active and cryptic prophages, and Hfq binding sites were identified throughout these domains. Overall the density of predicted sRNA genes in pathogenicity islands is ~1.8-fold greater than in the core genome.

The four most abundant unannotated sRNAs identified in this study were homologous and encoded at conserved positions within convergent sRNA pairs, 3′ of $P_{R'}$ in the so-called "moron" insertion site of lambdoid prophages. We have called this group of RNAs "anti-sRNA," as two members tested antagonize the function of core genome encoded sRNAs. All four anti-sRNA were between 51 and 60 nt in length, with highly conserved 3′ regions (nucleotides ~18–60) and variable 5′ ends. We initally examined AsxR (EcOnc02), as this is encoded 3′ and antisense to the Shiga toxin 2 transcript. Shiga toxins are responsible for the cellular pathology that leads to capillary damage and hemorrhage in EHEC-infected individuals that can lead to potentially fatal HUS.

Pulsed expression of AsxR stablised *chuS* mRNA, which encodes a heme oxygenase required for release of iron from heme. The core genome-encoded sRNA, FnrS, repressed *chuS* translation and was destabilized in the presence of AsxR. The 5′ region of AsxR is complementary to the single-stranded loop of the FnrS Rho-independent terminator. FnrS was destabilized by AsxR, consistent with AsxR hybridization unfolding the terminator stem that protects the 3′ end from exonucleolytic attack (Blum et al., 1999; Cisneros et al., 1996; Figueroa-Bossi et al., 2009). A similar mechanism of sRNA destabilization has been proposed for ChiX (MicM), an sRNA that is destabilized by an intercistronic region of the *chbBC* transcript with complementarity to the terminator stem of ChiX (Figueroa-Bossi et al., 2009).

FnrS is likely to be transcribed under the predominately anaerobic conditions of the gastrointestinal tract lumen, repressing ChuS translation. We suggest that expression of AsxR from the Shiga-toxin-2-encoding bacteriophage derepresses ChuS, potentially under the microaerophilic conditions associated with the epithelium to which the bacteria attach. The presence of AsxR within the *stx2AB* locus suggests that coordinating

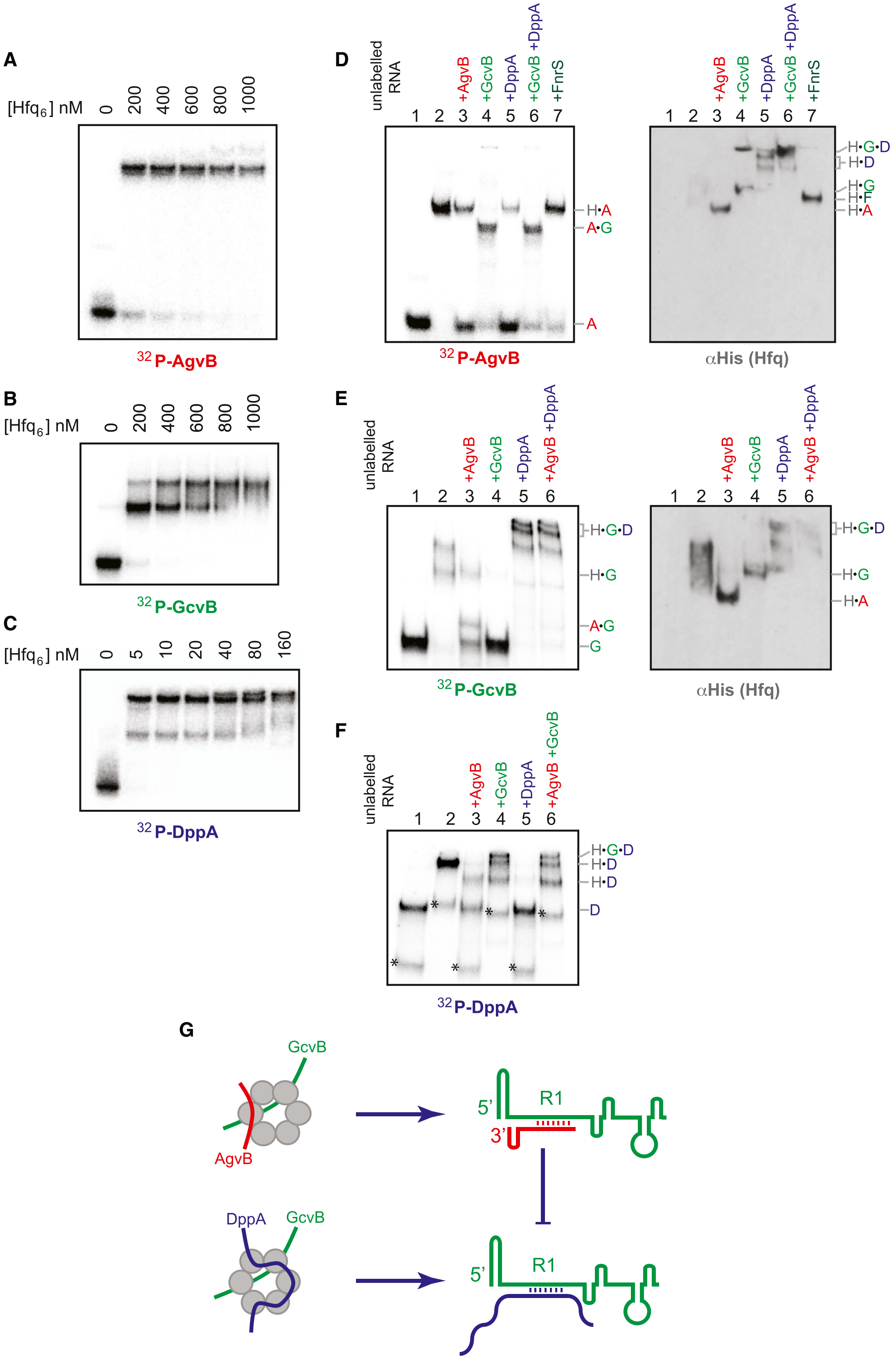

(legend on next page)

heme release and uptake by the lytic and lysogenic bacterial populations, respectively, are selected, coinherited traits. Such anti-sRNA regulation adds to the ways in which an integrated prophage can modify expression in the host bacterium and impact on colonization and disease (Xu et al., 2012).

The 5′ variable region of the most abundant anti-sRNA, AgvB, matches the consensus binding motif (CACAACA) for the core sRNA GcvB R1 seed region. GcvB is a key regulator of amino acid catabolism and transport (Sharma et al., 2011), repressing translation of numerous proteins, including the dipeptide transporter DppA. Expression of AgvB in *E. coli* K12 did not appreciably destabilize GcvB, but it relieved translational repression of DppA in reporter constructs. Loss of AvgB from *E. coli* O157:H7 reduced the translation efficiency of $DppA_{EHEC}$, indicating that AgvB indeed modulates translation in the pathogen.

AgvB fits the model of a small RNA and might have been expected to interact with the proximal face of Hfq through its Rho-independent terminator and/or U-U motif 5′ of the terminator stem. However, gel mobility shift analysis indicated that AgvB and DppA mRNA were able to displace each other from Hfq. DppA is strongly predicted to associate with the distal face of Hfq, suggesting that this is also the case for AgvB. Sequence analysis of AgvB identified a distal face binding motif (ARN4m1 and ARN5m2) within its 5′ variable region, and Hfq binding at this site is supported by CRAC data and the observation that AgvB is partly destabilized by introduction of a G1 mutation into this motif (Figure 6B, AgvB northern, lanes 10 and 11). In contrast, ternary complex formation was seen between Hfq, GcvB, and DppA mRNA. This indicates that these RNAs bind distinct, proximal and distal, sites on Hfq, potentially favoring duplex formation, using the rim arginines to reduce electrostatic repulsion (Panja et al., 2013). Consistent with in vitro duplex formation between AgvB and GcvB was facilited by Hfq (Figures 7D and 7E).

Riboregulation is an important posttranscriptional process generally responding to environmental conditions and therefore critical for adaptation to specific niches, including those encountered during colonization of the mammalian host by pathogenic bacteria. Horizontal acquisition of genomic regions by phage transfer endows the recipient bacterium with new genomic material, including genes that control ‘‘core’’ genome function. Two copies of AgvB are maintained in *E. coli* O157:H7, and deletion of both copies of AgvB reduced the competitiveness of the strain in mucus from the bovine terminal rectum, but not in rich (LB broth) or minimal (M9) media. The terminal rectum is the main colonization site for the bacterium in the reservoir host, supporting a function for the anti-sRNA in colonization of this specific niche. The characterized AgvB target, GcvB, is a global regulator that controls translation of up to 1% of transcripts. The majority are associated with amino acid and peptide uptake systems (Sharma et al., 2011), but the GcvB target(s) that contribute to enhanced growth at this site remain to be established.

The identification of ‘‘anti-sRNAs’’ has defined another layer of gene expression control in bacteria and a regulatory process that is important for niche adaptation in pathogenic *E. coli*.

EXPERIMENTAL PROCEDURES

Strain and Plasmid Construction

Strains used in this study are listed in Table S3A. *E. coli* O157:H7 str. Sakai stx− is a Shiga toxin negative derivative of the sequenced isolate O157:H7 str. Sakai (NCBI genome accession number NC_002695.1). For genetic manipulations, strains were grown in LB broth or plates supplemented with ampicillin (50 μg/ml), kanamycin (50 μg/ml), tetracycline (15 μg/ml), or chloramphenicol (25 μg/ml) where appropriate. The HTF tag contains His_6, a TEV protease cleavage site, and 3×FLAG affinity tag. Chromosomal replacement of *hfq* with *hfq*-HTF in both *E. coli* strains was carried out by allelic exchange, as was deletion of both copies of *agvB* from *E. coli* O157 str. Sakai. To monitor sRNA and anti-sRNA activity on translation of specific genes, a three-plasmid system was used with GFP translational fusion to the open reading frame of interest. Point mutations were introduced into the sRNA, anti-sRNA, or mRNA sequence by PCR amplification using mutagenic primers. Full descriptions are provided in the Supplemental Experimental Procedures section in Supplementary Information.

UV CRAC

Hfq CRAC was performed essentially as described by Granneman et al. (2011), except cell lysates were initially purified over anti-FLAG M2 affinity gel (Sigma, A2220). In summary, *E. coli* expressing the chromosomal Hfq-HTF was cultured under the required conditions and then subjected to UV irradiation in a stainless steel cylinder for 90 s. Cells were harvested and disrupted and Hfq-RNA complexes were purified on an anti-FLAG resin. The complexes were cleaned, treated with TEV protease, and trimmed with RNase before a second round of purification under guanidine hydrochloride denaturing conditions using Ni-NTA resin. Linker and 5′ ^{32}P labeling were carried out followed by gel electrophoresis, complex purification, and Protease K digestion. Released RNA was revese transcribed, the cDNA amplified by PCR, and the products separated by gel electrophoresis. Products over primer-dimer size were extracted and sequenced. Full details of this CRAC procedure are provided in the Supplemental Information. The Pearson correlations ranged from 0.49 to 0.95 between experiments. For K12, 93% of read clusters

Figure 7. EMSA Analysis in Hfq-AgvB Interactions

(A–C) Approximately 40 fmol of in-vitro-transcribed, radiolabeled AgvB (A), GcvB (B), or the 5′ 166 nt of $DppA_{Sal}$ (C) were incubated with increasing amounts of Hfq_6 (indicated above).

(D–F) (Left panels) Competition assays with unlabelled RNAs. Radiolabelled AgvB (D), GcvB (E), or DppA (F), were incubated in the absence (lane 1) or presence of 500 nM Hfq_6 (AgvB and GcvB) or 50 nM Hfq_6 (DppA) (lanes 2–7). Hfq binding reactions were additionally incubated in the presence of a 50-fold excess of unlabelled competitor RNAs (indicated above gel, lanes 3–7). The composition of complexes is indicated on the right-hand side (H = Hfq, A = AgvB, G = GcvB, and D = DppA). For radiolabeled DppA (F), a shorter DppA RNA fragment copurified with the full-length product and is indicated by an asterisk.

(D and E) (Right panels) αHis western blot analysis of EMSA gels to monitor the presence of His_6-tagged Hfq in gel-shifted complexes. Lanes are as in the left panels. In lanes E2 and E3, Hfq migrates as a smear, probably because it copurifies with heterogenous RNA species (Sittka et al., 2008), which are displaced in the presence of higher added concentrations of RNAs. In Figure 7F, the low Hfq concentration (50 nM) was not detectable by western analysis in DppA EMSA gels.

(G) Model for interaction of AgvB with Hfq, GcvB, and DppA. AgvB binds the distal face of Hfq (see also Figure S4) and forms a duplex with the R1 region of GcvB. Occlusion of the R1 region of GcvB prevents interactions between GcvB and the mRNA DppA. AgvB may also displace DppA from Hfq, although this interaction would be expected to be much more transient than inhibition through occlusion of GcvB R1. In the absence of AgvB, Hfq facilitates duplex formation between DppA and GcvB, repressing translation of DppA.

overlapped between the experiments, although the Pearson correlation was less significant (0.31), probably due to a lower number of sequences in one replicate. 5′ RLM-RACE was used to map the 5′ end of transcripts and to distinguish primary triphosphate from monophosphorylated 5′ ends. Full details of the in silico analysis of Hfq crosslinked sequences, including motif analyses, experimentally verified mRNA and sRNA seed sequence analyses, identification of unannotated sRNA sequences, and in silico prediction of sRNA and anti-sRNA targets, are provided in the Supplemental Experimental Procedures within the Supplementary Information.

Microarray Analysis of AsxR

For pulsed expression studies, *E. coli* O157:H7 str. TUV93-0 (deleted for both Stx phage) harboring pBAD+1 or pBAD+1::AsxR was grown to OD_{600} 0.8 in MEM-HEPES media and induced with 0.2% L-arabinose for 10 min. Microarray analysis was performed essentially as previously described (Tree et al., 2011).

Northern Blots

Total RNA was extracted by GTC-Phenol extraction. Five micrograms of total RNA was separated on an 8% polyacrylamide TBE-Urea gel and transferred to a nylon membrane and UV crosslinked. Membranes were prehybridized in 5 ml of UltraHyb Oligo Hyb (Ambion) and probed with 10 pmol of ^{32}P end-labeled 35-mer DNA oligo (Table S3C).

Fluorescent Reporters of Translation

The three plasmid system for expression of GFP and superfolder GFP translational fusions in anti-sRNA and sRNA expressing backgrounds were performed in *E. coli* DH5α, for *chu* operon and fragment fusions, and *E. coli* Top10F′ for $DppA_{Sal.}$ Cultures were grown overnight in LB before inoculation into M9 or MEM-HEPES at a 1:100 dilution. Fluorescence was measured either using an Infinite M200 microplate reader (Tecan) or a FLUOstar Optima fluorescence plate reader (BMG Labtech, Germany) with fluorescence measurements normalized to OD_{600}.

Electrophoretic Mobility Shift Assays

For analysis of Hfq binding to single RNAs, ∼40 pmol of labeled RNA was incubated with increasing Hfq in 1× Binding Buffer (10 mM Tris-HCl [pH 7.4], 0.1 mM EDTA, 10 mM NH_4Cl, 10 mM NaCl, and 10 mM KCl), 1 mg/ml tRNA, and 4% glycerol + bromophenol blue. Reactions were incubated at room temperature for 5 min and separated on a native 5% polyacrylamide 0.5× TBE gel. For analysis of complexes formed in the presence of multiple RNAs, binding buffer was replaced with Duplex Buffer (40 mM Tris-Acetate, 0.5 mM Magnesium Acetate, and 100 mM NaCl). 0.5× TBE was also replaced with 1× Duplex Buffer in both native 5% polyacrylamide gels and running buffer. Approximately 40 pmol of ^{32}P-labeled RNA was incubated either 500 nM (AgvB and GcvB) or 50 nM Hfq (DppA) in the presence of a 50-fold excess of unlabelled RNA. Reactions were incubated at room temperature for 15 min and separated on polyacrylamide gels.

Competitive Index Experiments

Ten microlitres of each strain was added to 5 ml of LB, 5 ml of MEM-HEPES (supplemented with supplemented with 250 nM $Fe(NO_3)_3$ and 0.1% glucose), or 1 ml of 10% bovine TRM diluted in sterile water. Six batches of mucus were prepared, with a single batch made up of mucus collected from five different animals. Cultures were grown overnight with shaking at 37°C and 10 μl transferred into fresh media of the same for overnight growth. Cultures were serially diluted and plated on LB plates containing kanamycin (both strains) or kanamycin + tetracycline (test strain) and cell numbers enumerated from serial dilutions.

ACCESSION NUMBERS

Sequencing and array data is available at GEO under the accession numbers GSE46118 (sequencing) and GSE46113 (array) within the superseries GSE46120.

SUPPLEMENTAL INFORMATION

Supplemental Information includes four figures, three tables, and Supplemental Experimental Procedures and can be found with this article online at http://dx.doi.org/10.1016/j.molcel.2014.05.006.

ACKNOWLEDGMENTS

The descriptor for a Rho-independent terminator using RNAMotif was kindly provided by David J. Ecker. The pXG superfolder GFP series of plasmids were kindly supplied by Jörg Vogel. The authors thank Simon Lebaron for purifying Hfq-His, Gregorz Kudla for assistance with analysis, and Clark Russell for help with strain construction. We would like to thank Nur Indah Ahmad, Alex Corbishley, and Tom McNeilly for helping collect bovine mucus samples. This work was made possible by a Wellcome Trust project grant to D.G. and D.T. (WT090231MA). Research at the Roslin Institute is supported by BBSRC Institute grant funding. Work in the Wellcome Trust Centre for Cell Biology is supported by Wellcome Trust core funding (092076).

Received: February 11, 2014
Revised: March 21, 2014
Accepted: May 1, 2014
Published: June 5, 2014

REFERENCES

Bandyra, K.J., and Luisi, B.F. (2013). Licensing and due process in the turnover of bacterial RNA. RNA Biol. *10*, 627–635.

Beisel, C.L., Updegrove, T.B., Janson, B.J., and Storz, G. (2012). Multiple factors dictate target selection by Hfq-binding small RNAs. EMBO J. *31*, 1961–1974.

Blum, E., Carpousis, A.J., and Higgins, C.F. (1999). Polyadenylation promotes degradation of 3′-structured RNA by the Escherichia coli mRNA degradosome in vitro. J. Biol. Chem. *274*, 4009–4016.

Bossi, L., Schwartz, A., Guillemardet, B., Boudvillain, M., and Figueroa-Bossi, N. (2012). A role for Rho-dependent polarity in gene regulation by a noncoding small RNA. Genes Dev. *26*, 1864–1873.

Bouvier, M., Sharma, C.M., Mika, F., Nierhaus, K.H., and Vogel, J. (2008). Small RNA binding to 5′ mRNA coding region inhibits translational initiation. Mol. Cell *32*, 827–837.

Cao, Y., Wu, J., Liu, Q., Zhao, Y., Ying, X., Cha, L., Wang, L., and Li, W. (2010). sRNATarBase: a comprehensive database of bacterial sRNA targets verified by experiments. RNA *16*, 2051–2057.

Chao, Y., and Vogel, J. (2010). The role of Hfq in bacterial pathogens. Curr. Opin. Microbiol. *13*, 24–33.

Chao, Y., Papenfort, K., Reinhardt, R., Sharma, C.M., and Vogel, J. (2012). An atlas of Hfq-bound transcripts reveals 3′ UTRs as a genomic reservoir of regulatory small RNAs. EMBO J. *31*, 4005–4019.

Cisneros, B., Court, D., Sanchez, A., and Montañez, C. (1996). Point mutations in a transcription terminator, lambda tI, that affect both transcription termination and RNA stability. Gene *181*, 127–133.

Corcoran, C.P., Podkaminski, D., Papenfort, K., Urban, J.H., Hinton, J.C., and Vogel, J. (2012). Superfolder GFP reporters validate diverse new mRNA targets of the classic porin regulator, MicF RNA. Mol. Microbiol. *84*, 428–445.

Durand, S., and Storz, G. (2010). Reprogramming of anaerobic metabolism by the FnrS small RNA. Mol. Microbiol. *75*, 1215–1231.

Fender, A., Elf, J., Hampel, K., Zimmermann, B., and Wagner, E.G. (2010). RNAs actively cycle on the Sm-like protein Hfq. Genes Dev. *24*, 2621–2626.

Figueroa-Bossi, N., Valentini, M., Malleret, L., Fiorini, F., and Bossi, L. (2009). Caught at its own game: regulatory small RNA inactivated by an inducible transcript mimicking its target. Genes Dev. *23*, 2004–2015.

Granneman, S., Petfalski, E., and Tollervey, D. (2011). A cluster of ribosome synthesis factors regulate pre-rRNA folding and 5.8S rRNA maturation by the Rat1 exonuclease. EMBO J. *30*, 4006–4019.

Hayashi, T., Makino, K., Ohnishi, M., Kurokawa, K., Ishii, K., Yokoyama, K., Han, C.G., Ohtsubo, E., Nakayama, K., Murata, T., et al. (2001). Complete genome sequence of enterohemorrhagic Escherichia coli O157:H7 and genomic comparison with a laboratory strain K-12. DNA Res. *8*, 11–22.

Hopkins, J.F., Panja, S., and Woodson, S.A. (2011). Rapid binding and release of Hfq from ternary complexes during RNA annealing. Nucleic Acids Res. *39*, 5193–5202.

Ishikawa, H., Otaka, H., Maki, K., Morita, T., and Aiba, H. (2012). The functional Hfq-binding module of bacterial sRNAs consists of a double or single hairpin preceded by a U-rich sequence and followed by a 3′ poly(U) tail. RNA *18*, 1062–1074.

Juhala, R.J., Ford, M.E., Duda, R.L., Youlton, A., Hatfull, G.F., and Hendrix, R.W. (2000). Genomic sequences of bacteriophages HK97 and HK022: pervasive genetic mosaicism in the lambdoid bacteriophages. J. Mol. Biol. *299*, 27–51.

Keseler, I.M., Mackie, A., Peralta-Gil, M., Santos-Zavaleta, A., Gama-Castro, S., Bonavides-Martínez, C., Fulcher, C., Huerta, A.M., Kothari, A., Krummenacker, M., et al. (2013). EcoCyc: fusing model organism databases with systems biology. Nucleic Acids Res. *41* (Database issue), D605–D612.

Lalaouna, D., Simoneau-Roy, M., Lafontaine, D., and Massé, E. (2013). Regulatory RNAs and target mRNA decay in prokaryotes. Biochim. Biophys. Acta *1829*, 742–747.

Le Derout, J., Folichon, M., Briani, F., Dehò, G., Régnier, P., and Hajnsdorf, E. (2003). Hfq affects the length and the frequency of short oligo(A) tails at the 3′ end of Escherichia coli rpsO mRNAs. Nucleic Acids Res. *31*, 4017–4023.

Lease, R.A., and Woodson, S.A. (2004). Cycling of the Sm-like protein Hfq on the DsrA small regulatory RNA. J. Mol. Biol. *344*, 1211–1223.

Link, T.M., Valentin-Hansen, P., and Brennan, R.G. (2009). Structure of Escherichia coli Hfq bound to polyriboadenylate RNA. Proc. Natl. Acad. Sci. USA *106*, 19292–19297.

Moll, I., Leitsch, D., Steinhauser, T., and Bläsi, U. (2003). RNA chaperone activity of the Sm-like Hfq protein. EMBO Rep. *4*, 284–289.

Naylor, S.W., Low, J.C., Besser, T.E., Mahajan, A., Gunn, G.J., Pearce, M.C., McKendrick, I.J., Smith, D.G., and Gally, D.L. (2003). Lymphoid follicle-dense mucosa at the terminal rectum is the principal site of colonization of enterohemorrhagic Escherichia coli O157:H7 in the bovine host. Infect. Immun. *71*, 1505–1512.

Naylor, S.W., Roe, A.J., Nart, P., Spears, K., Smith, D.G., Low, J.C., and Gally, D.L. (2005). Escherichia coli O157 : H7 forms attaching and effacing lesions at the terminal rectum of cattle and colonization requires the LEE4 operon. Microbiology *151*, 2773–2781.

Otaka, H., Ishikawa, H., Morita, T., and Aiba, H. (2011). PolyU tail of rho-independent terminator of bacterial small RNAs is essential for Hfq action. Proc. Natl. Acad. Sci. USA *108*, 13059–13064.

Panja, S., Schu, D.J., and Woodson, S.A. (2013). Conserved arginines on the rim of Hfq catalyze base pair formation and exchange. Nucleic Acids Res. *41*, 7536–7546.

Papenfort, K., and Vogel, J. (2010). Regulatory RNA in bacterial pathogens. Cell Host Microbe *8*, 116–127.

Pfeiffer, V., Papenfort, K., Lucchini, S., Hinton, J.C., and Vogel, J. (2009). Coding sequence targeting by MicC RNA reveals bacterial mRNA silencing downstream of translational initiation. Nat. Struct. Mol. Biol. *16*, 840–846.

Raghavan, R., Groisman, E.A., and Ochman, H. (2011). Genome-wide detection of novel regulatory RNAs in E. coli. Genome Res. *21*, 1487–1497.

Sauer, E., and Weichenrieder, O. (2011). Structural basis for RNA 3′-end recognition by Hfq. Proc. Natl. Acad. Sci. USA *108*, 13065–13070.

Sauer, E., Schmidt, S., and Weichenrieder, O. (2012). Small RNA binding to the lateral surface of Hfq hexamers and structural rearrangements upon mRNA target recognition. Proc. Natl. Acad. Sci. USA *109*, 9396–9401.

Schumacher, M.A., Pearson, R.F., Møller, T., Valentin-Hansen, P., and Brennan, R.G. (2002). Structures of the pleiotropic translational regulator Hfq and an Hfq-RNA complex: a bacterial Sm-like protein. EMBO J. *21*, 3546–3556.

Sharma, C.M., Darfeuille, F., Plantinga, T.H., and Vogel, J. (2007). A small RNA regulates multiple ABC transporter mRNAs by targeting C/A-rich elements inside and upstream of ribosome-binding sites. Genes Dev. *21*, 2804–2817.

Sharma, C.M., Papenfort, K., Pernitzsch, S.R., Mollenkopf, H.J., Hinton, J.C., and Vogel, J. (2011). Pervasive post-transcriptional control of genes involved in amino acid metabolism by the Hfq-dependent GcvB small RNA. Mol. Microbiol. *81*, 1144–1165.

Sittka, A., Lucchini, S., Papenfort, K., Sharma, C.M., Rolle, K., Binnewies, T.T., Hinton, J.C., and Vogel, J. (2008). Deep sequencing analysis of small noncoding RNA and mRNA targets of the global post-transcriptional regulator, Hfq. PLoS Genet. *4*, e1000163.

Soper, T.J., and Woodson, S.A. (2008). The rpoS mRNA leader recruits Hfq to facilitate annealing with DsrA sRNA. RNA *14*, 1907–1917.

Sudo, N., Soma, A., Muto, A., Iyoda, S., Suh, M., Kurihara, N., Abe, H., Tobe, T., Ogura, Y., Hayashi, T., et al. (2014). A novel small regulatory RNA enhances cell motility in enterohemorrhagic Escherichia coli. J. Gen. Appl. Microbiol. *60*, 44–50.

Tarr, P.I., Gordon, C.A., and Chandler, W.L. (2005). Shiga-toxin-producing Escherichia coli and haemolytic uraemic syndrome. Lancet *365*, 1073–1086.

Tildesley, M.J., Gally, D.L., McNeilly, T.N., Low, J.C., Mahajan, A., and Savill, N.J. (2012). Insights into mucosal innate responses to Escherichia coli O157 : H7 colonization of cattle by mathematical modelling of excretion dynamics. J. R. Soc. Interface *9*, 518–527.

Tree, J.J., Roe, A.J., Flockhart, A., McAteer, S.P., Xu, X., Shaw, D., Mahajan, A., Beatson, S.A., Best, A., Lotz, S., et al. (2011). Transcriptional regulators of the GAD acid stress island are carried by effector protein-encoding prophages and indirectly control type III secretion in enterohemorrhagic Escherichia coli O157:H7. Mol. Microbiol. *80*, 1349–1365.

Updegrove, T., Wilf, N., Sun, X., and Wartell, R.M. (2008). Effect of Hfq on RprA-rpoS mRNA pairing: Hfq-RNA binding and the influence of the 5′ rpoS mRNA leader region. Biochemistry *47*, 11184–11195.

Urbanowski, M.L., Stauffer, L.T., and Stauffer, G.V. (2000). The gcvB gene encodes a small untranslated RNA involved in expression of the dipeptide and oligopeptide transport systems in Escherichia coli. Mol. Microbiol. *37*, 856–868.

Webb, S., Hector, R.D., Kudla, G., and Granneman, S. (2014). PAR-CLIP data indicate that Nrd1-Nab3-dependent transcription termination regulates expression of hundreds of protein coding genes in yeast. Genome Biol. *15*, R8.

Xu, X., McAteer, S.P., Tree, J.J., Shaw, D.J., Wolfson, E.B., Beatson, S.A., Roe, A.J., Allison, L.J., Chase-Topping, M.E., Mahajan, A., et al. (2012). Lysogeny with Shiga toxin 2-encoding bacteriophages represses type III secretion in enterohemorrhagic Escherichia coli. PLoS Pathog. *8*, e1002672.

Yang, Q., Figueroa-Bossi, N., and Bossi, L. (2014). Translation enhancing ACA motifs and their silencing by a bacterial small regulatory RNA. PLoS Genet. *10*, e1004026.

Zhang, A., Schu, D.J., Tjaden, B.C., Storz, G., and Gottesman, S. (2013). Mutations in interaction surfaces differentially impact E. coli Hfq association with small RNAs and their mRNA targets. J. Mol. Biol. *425*, 3678–3697.

Molecular Cell, Volume *55*

Supplemental Information

Identification of Bacteriophage-Encoded Anti-sRNAs in Pathogenic *Escherchia coli*

Jai J. Tree, Sander Granneman, Sean P. McAteer, David Tollervey, and David L. Gally

SUPPLEMENTARY INFORMATION

Figure S1

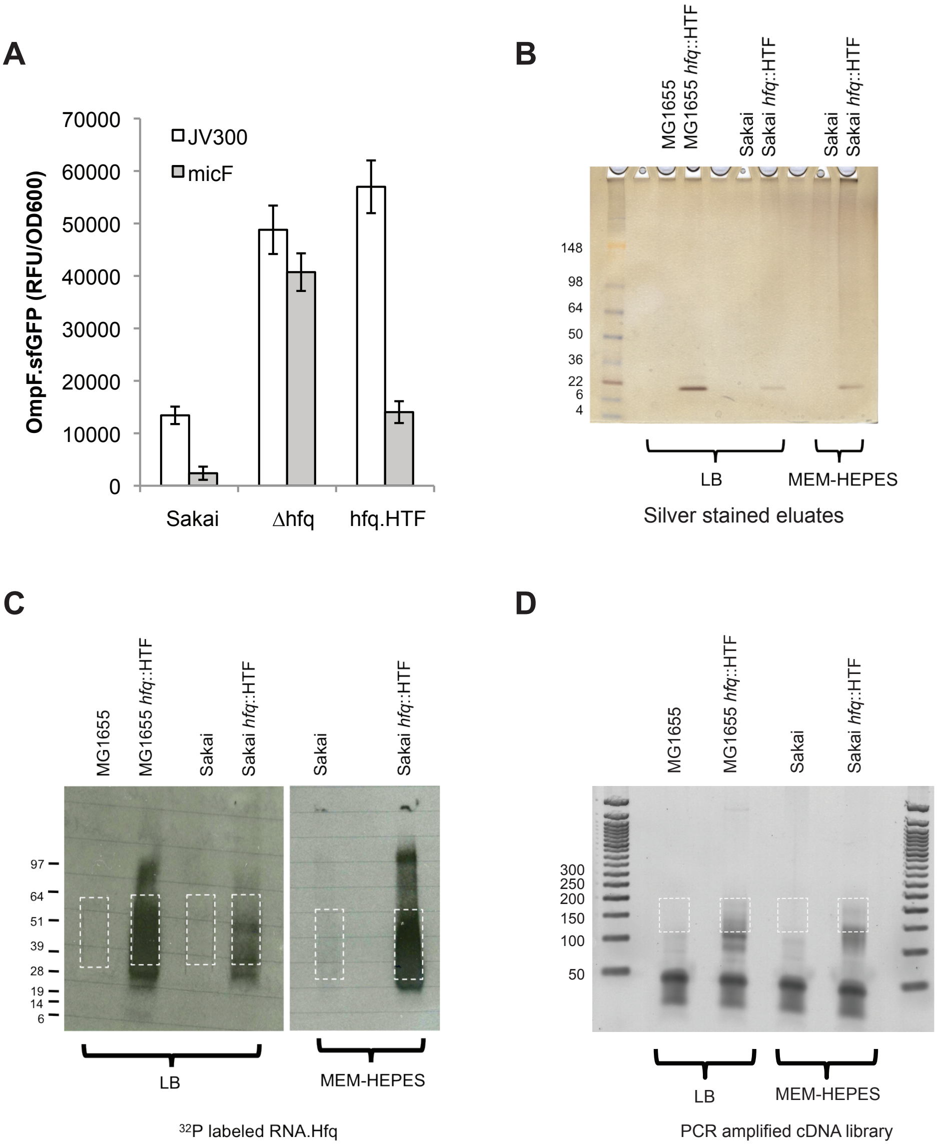

Figure S1, related to Figure 1 and Experimental Procedures. His-FLAG tagged Hfq is functional and allows stringent purification of UV-crosslinked RNAs.

A. The previously reported interactions between MicF and OmpF (Urban and Vogel, 2007) were used to assess functionality of the dual affinity tagged Hfq construct (See also *Functional assay for Hfq-HTF activity* in Extended Experimental Procedures). Fluorescence of the OmpF fusion was measured in *E. coli* O157:H7 str. Sakai (Sakai), an isogenic *hfq* mutant (Δ*hfq*), or the His-FLAG tagged *hfq* mutant (hfq.HTF), in the presence the control plasmid pJV300 or pMI expressing MicF. Error bars show SEM. B-D. Purification of Hfq.RNA complexes (see also Figure 1). B. Silver stained eluates from control and HTF tagged *hfq* mutants in non-pathogenic *E. coli* K12 str. MG1655 (MG1655), and *E. coli* O157:H7 str. Sakai (Sakai) grown in LB broth,

and Sakai grown in MEM-HEPES media. C. Autoradiogram of purified, ^{32}P-labelled RNA crosslinked to Hfq separated by SDS-PAGE and transferred to a nitrocellulose membrane (cultures as listed for B). Regions indicated by white dashed boxes were cut from the membrane and RNA isolated for cDNA library construction. D. Agarose gel electrophoresis of PCR products amplified from cDNA libraries. Regions indicated in the white dashed boxes were gel extracted and submitted for Illumina high throughput sequencing.

Figure S2

ARN3_m0

```
>cycA_GcvB      ACAGATTCGTCGTATCATAGACTGACTAAAGGCCGTAGAGCCTGAACAACACAGACAGGTACAGGAAGAAAAAAACATGGTAGATCAGGTAAAAGTCGTTGCCGATGATCAGGCTCCGGCTGAACAGTCGCTAC
>fhlA_OxyS      TAAATCTCCTATAGTTAGTCAATGACCTTTTGCACCGCTTTGCGGTGCTTTCCTGGAAGAACAAAATGTCATATACACCGATGAGTGATCTCGGACAGCAAGGGTTG
>fur_RyhB       TGCCACGTTTTTATTAACAATATTTGCCAGGGACTTGTGGTTTTCATTTAGGCGTGGCAATTCTATAATGATACGCATTATCTCAAGAGCAAATTCTGTCACTTCTTCTAATGAAGTGAACCGCTTAGTAACAGGACAGATTCCGCATGA
>fur_RyhB       TGCCACGTTTTTATTAACAATATTTGCCAGGGACTTGTGGTTTTCATTTAGGCGTGGCAATTCTATAATGATACGCATTATCTCAAGAGCAAATTCTGTCACTTCTTCTAATGAAGTGAACCGCTTAGTAACAGGACAGATTCCGCATGA
>gltA_spf       TTCGCCATTTATTCGTCATCAATGGATCCTTTACCTGCAAGCGCCCAGAGCTCTGTACCCAGGTTTTCCCCTCTTTCACAGAGCGGCGAGCCAAATAACAAACGGGTAAA
>lrp_micF       GATGTTTCAGGGTCAGACAGGAGTAGGGAAGGAATACAGAGAGACAATAATAATGGTAGATAGCAAGAAGCGCCCTGGCAAAGATCTCGACCGTATCGATCGTAACATTCTTAA
>lrp_micF       GATGTTTCAGGGTCAGACAGGAGTAGGGAAGGAATACAGAGAGACAATAATAATGGTAGATAGCAAGAAGCGCCCTGGCAAAGATCTCGACCGTATCGATCGTAACATTCTTAA
>lrp_micF       GATGTTTCAGGGTCAGACAGGAGTAGGGAAGGAATACAGAGAGACAATAATAATGGTAGATAGCAAGAAGCGCCCTGGCAAAGATCTCGACCGTATCGATCGTAACATTCTTAA
>luxS_CyaR      TCAGACTCGCCTGGGAAGAAAGAGTTCAGAAAATTTTTAAAAAAATTACCGGAGGTGGCTAAATGCCGTTGTTAGATAGCTTCACAGTCGATCATACCCGGATGGAAGCGCCTGCA
>metE_FnrS      TGAATAAACTTGCCGCCTTCCCTAAATTCAAAATCCATAGGATTTACATATAATTAGAGGAAGAAAAATGACAATATTGAATCACACCCTCGGTTTCCCTCGCGTTGGCCTGCGTCGCGAGCTGAAAAAAG
>nadE_CyaR      CGTTTCAGTAGCAACGGGTTAGCTTTAAGGAAGTTTTGTCTTTTCTGTCTGGAGGGGTTCAATGACATTGCAACAACAAATAATAAAGGCGCTGGGCGCAAAACCGCAGATTAAT
>nadE_CyaR      CGTTTCAGTAGCAACGGGTTAGCTTTAAGGAAGTTTTGTCTTTTCTGTCTGGAGGGGTTCAATGACATTGCAACAACAAATAATAAAGGCGCTGGGCGCAAAACCGCAGATTAAT
>ndk_gcvB       CAACGGCTCTGTTGCTCATTATAATCCGCGCCATCTCGTACGCTGGTACAGACAACAACAGAACAATTTACAGAGGTAAAAATGGCTATTGAACGTACTTTTTCCATTATCAAACC
>ompC_MicC      GAGAATGGACTTGCCGACTGATTAATGAGGGTTAATCAGTATGCAGTGGCATAAAAAAGCAAATAAAGGCATATAACAGAGGGTTAATAACATGAAAGTTAAAGTACTGTCCCTCCTGGTCCCAGCT
>ompC_RseX      TTGCCGACTGATTAATGAGGGTTAATCAGTATGCAGTGGCATAAAAAAGCAAATAAAGGCATATAACAGAGGGTTAATAACATGAAAGTTAAAGTACTGTCCCTCCTGGTCCCAGCTCTGCTGGTAGCAGG
>ompC_RybB      TGCATTTTTGGGGAGAATGGACTTGCCGACTGATTAATGAGGGTTAATCAGTATGCAGTGGCATAAAAAAGCAAATAAAGGCATATAACAGAGGGTTAATAACATGAAAGTTAAAGTACTGTCCCTCCTGGTCCCAGCTCTGCTGGTAGC
>ompF_MicF      TTTATTGACAGAAATTATTGACGGCAGTGGCAGGTGTCATAAAAAAAACCATGAGGGTAATAAATAATGATGAAGCGCAATATTCTGGCAGTGATCGTCCCTGCTCTGTTAGTAGCAGGTACTGCAA
>ompF_MicF      TTTATTGACAGAAATTATTGACGGCAGTGGCAGGTGTCATAAAAAAAACCATGAGGGTAATAAATAATGATGAAGCGCAATATTCTGGCAGTGATCGTCCCTGCTCTGTTAGTAGCAGGTACTGCAA
>ompW_RybB      TCATTTGTTACTCCAATGTAGGTATATTCGTCACGTTTTTATAACCATAACGACGGAGCGGATATGAAAAAGTTAACAGTGGCGGCTTTGGCAGTAACAACTCTTCTCTCTGGCAGTGCCTTTGCGCATGAAGC
>phoP_MicA      GTTTACCCCCATAACCACATAATCGCGTTACACTATTTTAATAATTAAGACAGGGAGAAATAAAAATGCGCGTACTGGTTGTTGAAGACAATGCGTTGTTACGTCACCACCTTAAAGTTCAGAT
>ptsG_SgrS      TGTACACGGCGAGGCTCTCCCCCCTTGCCACGCGTGAGAACGTAAAAAAAGCACCCATACTCAGGAGCACTCTCAATTATGTTTAAGAATGCATTTGCTAACCTGCAAAAGGTCGGTAAATCGCTGATGCTGC
>rpoS_DsrA      AAATAGCAACCATGGGTAGCACCGGAACCAGTTCAACACGCTTGCATTTTGAAATTCGTTACAAGGGGAAATCCGTAAACCCGCTGCGTTATTTGCCGCAGCGATAAATCGGCGGAACCAGGC
>sodB_FnrS      CACCTTTTAATTTGCTACCCTATCCATACGCACAATAAGGCTATTGTACGTATGCAAATTAATAATAAAGGAGAGTAGCAATGTCATTCGAATTACCTGCACTACCATATGCTAAAGATGCTCTGGCACCGCACA
>sodB_FnrS      CACCTTTTAATTTGCTACCCTATCCATACGCACAATAAGGCTATTGTACGTATGCAAATTAATAATAAAGGAGAGTAGCAATGTCATTCGAATTACCTGCACTACCATATGCTAAAGATGCTCTGGCACCGCACA
>sodB_RyhB      GCTACCCTATCCATACGCACAATAAGGCTATTGTACGTATGCAAATTAATAATAAAGGAGAGTAGCAATGTCATTCGAATTACCTGCACTACCATATGCTAAAGATGCTCTGGCACCGCACATTTCT
>sodB_RyhB      GCTACCCTATCCATACGCACAATAAGGCTATTGTACGTATGCAAATTAATAATAAAGGAGAGTAGCAATGTCATTCGAATTACCTGCACTACCATATGCTAAAGATGCTCTGGCACCGCACATTTCT
>srlA_spf       CTTTCAATCTGATTAGATTAGGTTGCCGTTTGGTAATAAAACAATAAATCCTGAAGGAGAGAACAATGATAGAAACCATTACTCATGGTGCAGAGTGGTTTATCGGGCTGTTCCA
>tppB_gcvB      AAAGCACCCCCGTTAATATGGGATGTAAAAAAAGAGGTAAAAGTGTCCACTGCAAACCAAAAACCAACTGAAAGCGTCAGTTTGAACGCTTTCAAACAACCGAAGGCGTTCTATCTCATC
>yaeC_gcvB      TCAGTTCGCAGGCGACCGCATCGTCCGGGCTGTCACTCGCAAGTAACGTTCAACACAACATAAATAATTGAAGAAGGAATAAGGTATGGCGTTCAAATTCAAAACCTTTGCGGCAGTGGG
>ybdH_gcvB      TGCTAGTATTTGGCAATCAAGACGTTTAGATGTCTAAATAAAACAATAAGGACAACACAACATGCCTCACAATCCTATCCGCGTGGTCGTCGGCCCGGCTAACTACTTTTCAC
```

ARN4_m0

```
>cycA_GcvB      ACAGATTCGTCGTATCATAGACTGACTAAAGGCCGTAGAGCCTGAACAACACAGACAGGTACAGGAAGAAAAAAACATGGTAGATCAGGTAAAAGTCGTTGCCGATGATCAGGCTCCGGCTGAACAGTCGCTAC
>phoP_MicA      GTTTACCCCCATAACCACATAATCGCGTTACACTATTTTAATAATTAAGACAGGGAGAAATAAAAATGCGCGTACTGGTTGTTGAAGACAATGCGTTGTTACGTCACCACCTTAAAGTTCAGAT
>yaeC_gcvB      TCAGTTCGCAGGCGACCGCATCGTCCGGGCTGTCACTCGCAAGTAACGTTCAACACAACATAAATAATTGAAGAAGGAATAAGGTATGGCGTTCAAATTCAAAACCTTTGCGGCAGTGGG
```

ARN4_m1

```
>cycA_GcvB      ACAGATTCGTCGTATCATAGACTGACTAAAGGCCGTAGAGCCTGAACAACACAGACAGGTACAGGAAGAAAAAAACATGGTAGATCAGGTAAAAGTCGTTGCCGATGATCAGGCTCCGGCTGAACAGTCGCTAC
>cycA_GcvB      ACAGATTCGTCGTATCATAGACTGACTAAAGGCCGTAGAGCCTGAACAACACAGACAGGTACAGGAAGAAAAAAACATGGTAGATCAGGTAAAAGTCGTTGCCGATGATCAGGCTCCGGCTGAACAGTCGCTAC
>cycA_GcvB      ACAGATTCGTCGTATCATAGACTGACTAAAGGCCGTAGAGCCTGAACAACACAGACAGGTACAGGAAGAAAAAAACATGGTAGATCAGGTAAAAGTCGTTGCCGATGATCAGGCTCCGGCTGAACAGTCGCTAC
>fhlA_OxyS      TAAATCTCCTATAGTTAGTCAATGACCTTTTGCACCGCTTTGCGGTGCTTTCCTGGAAGAACAAAATGTCATATACACCGATGAGTGATCTCGGACAGCAAGGGTTG
>fhlA_OxyS      TAAATCTCCTATAGTTAGTCAATGACCTTTTGCACCGCTTTGCGGTGCTTTCCTGGAAGAACAAAATGTCATATACACCGATGAGTGATCTCGGACAGCAAGGGTTG
>fucI_spf       TTTATCTTTGCCCGTTTCCGTTCTCAAACGGCAACTAACTGAACATATTTTCCGAATAAAGTGAGGAATCTGTAATGAACAAAATCAGCTTACCGAAAATTGGTATCCGCCCGGTTATTGACGGTCGTCGCATGGGTGTTCGTGAGTCGCTTGAAGAA
>fur_RyhB       TGCCACGTTTTTATTAACAATATTTGCCAGGGACTTGTGGTTTTCATTTAGGCGTGGCAATTCTATAATGATACGCATTATCTCAAGAGCAAATTCTGTCACTTCTTCTAATGAAGTGAACCGCTTAGTAACAGGACAGATTCCGCATGA
>gdhA_gcvB      TGCTTACCGTCACATTCTTGATGGTATAGTCGAAAACTGCAAAAGCACATGACATAAACAACATAAGCACAATCGTATTAATATATAAGGGTTTTATATCTATGGATCAGACATATTCTCTGGAGTCA
>gdhA_gcvB      TGCTTACCGTCACATTCTTGATGGTATAGTCGAAAACTGCAAAAGCACATGACATAAACAACATAAGCACAATCGTATTAATATATAAGGGTTTTATATCTATGGATCAGACATATTCTCTGGAGTCA
>gltA_spf       TTCGCCATTTATTCGTCATCAATGGATCCTTTACCTGCAAGCGCCCAGAGCTCTGTACCCAGGTTTTCCCCTCTTTCACAGAGCGGCGAGCCAAATAACAAACGGGTAAA
>lrp_micF       GATGTTTCAGGGTCAGACAGGAGTAGGGAAGGAATACAGAGAGACAATAATAATGGTAGATAGCAAGAAGCGCCCTGGCAAAGATCTCGACCGTATCGATCGTAACATTCTTAA
>lrp_micF       GATGTTTCAGGGTCAGACAGGAGTAGGGAAGGAATACAGAGAGACAATAATAATGGTAGATAGCAAGAAGCGCCCTGGCAAAGATCTCGACCGTATCGATCGTAACATTCTTAA
>lrp_micF       GATGTTTCAGGGTCAGACAGGAGTAGGGAAGGAATACAGAGAGACAATAATAATGGTAGATAGCAAGAAGCGCCCTGGCAAAGATCTCGACCGTATCGATCGTAACATTCTTAA
>lrp_micF       GATGTTTCAGGGTCAGACAGGAGTAGGGAAGGAATACAGAGAGACAATAATAATGGTAGATAGCAAGAAGCGCCCTGGCAAAGATCTCGACCGTATCGATCGTAACATTCTTAA
>lrp_micF       GATGTTTCAGGGTCAGACAGGAGTAGGGAAGGAATACAGAGAGACAATAATAATGGTAGATAGCAAGAAGCGCCCTGGCAAAGATCTCGACCGTATCGATCGTAACATTCTTAA
>lrp_micF       GATGTTTCAGGGTCAGACAGGAGTAGGGAAGGAATACAGAGAGACAATAATAATGGTAGATAGCAAGAAGCGCCCTGGCAAAGATCTCGACCGTATCGATCGTAACATTCTTAA
>lrp_micF       GATGTTTCAGGGTCAGACAGGAGTAGGGAAGGAATACAGAGAGACAATAATAATGGTAGATAGCAAGAAGCGCCCTGGCAAAGATCTCGACCGTATCGATCGTAACATTCTTAA
>lrp_micF       GATGTTTCAGGGTCAGACAGGAGTAGGGAAGGAATACAGAGAGACAATAATAATGGTAGATAGCAAGAAGCGCCCTGGCAAAGATCTCGACCGTATCGATCGTAACATTCTTAA
>luxS_CyaR      TCAGACTCGCCTGGGAAGAAAGAGTTCAGAAAATTTTTAAAAAAATTACCGGAGGTGGCTAAATGCCGTTGTTAGATAGCTTCACAGTCGATCATACCCGGATGGAAGCGCCTGCA
>metE_FnrS      TGAATAAACTTGCCGCCTTCCCTAAATTCAAAATCCATAGGATTTACATATAATTAGAGGAAGAAAAATGACAATATTGAATCACACCCTCGGTTTCCCTCGCGTTGGCCTGCGTCGCGAGCTGAAAAAAG
>metE_FnrS      TGAATAAACTTGCCGCCTTCCCTAAATTCAAAATCCATAGGATTTACATATAATTAGAGGAAGAAAAATGACAATATTGAATCACACCCTCGGTTTCCCTCGCGTTGGCCTGCGTCGCGAGCTGAAAAAAG
>metE_FnrS      TGAATAAACTTGCCGCCTTCCCTAAATTCAAAATCCATAGGATTTACATATAATTAGAGGAAGAAAAATGACAATATTGAATCACACCCTCGGTTTCCCTCGCGTTGGCCTGCGTCGCGAGCTGAAAAAAG
>metE_FnrS      TGAATAAACTTGCCGCCTTCCCTAAATTCAAAATCCATAGGATTTACATATAATTAGAGGAAGAAAAATGACAATATTGAATCACACCCTCGGTTTCCCTCGCGTTGGCCTGCGTCGCGAGCTGAAAAAAG
>nadE_CyaR      CGTTTCAGTAGCAACGGGTTAGCTTTAAGGAAGTTTTGTCTTTTCTGTCTGGAGGGGTTCAATGACATTGCAACAACAAATAATAAAGGCGCTGGGCGCAAAACCGCAGATTAAT
>nadE_CyaR      CGTTTCAGTAGCAACGGGTTAGCTTTAAGGAAGTTTTGTCTTTTCTGTCTGGAGGGGTTCAATGACATTGCAACAACAAATAATAAAGGCGCTGGGCGCAAAACCGCAGATTAAT
>nadE_CyaR      CGTTTCAGTAGCAACGGGTTAGCTTTAAGGAAGTTTTGTCTTTTCTGTCTGGAGGGGTTCAATGACATTGCAACAACAAATAATAAAGGCGCTGGGCGCAAAACCGCAGATTAAT
>nadE_CyaR      CGTTTCAGTAGCAACGGGTTAGCTTTAAGGAAGTTTTGTCTTTTCTGTCTGGAGGGGTTCAATGACATTGCAACAACAAATAATAAAGGCGCTGGGCGCAAAACCGCAGATTAAT
>ndk_gcvB       CAACGGCTCTGTTGCTCATTATAATCCGCGCCATCTCGTACGCTGGTACAGACAACAACAGAACAATTTACAGAGGTAAAAATGGCTATTGAACGTACTTTTTCCATTATCAAACC
>ndk_gcvB       CAACGGCTCTGTTGCTCATTATAATCCGCGCCATCTCGTACGCTGGTACAGACAACAACAGAACAATTTACAGAGGTAAAAATGGCTATTGAACGTACTTTTTCCATTATCAAACC
>ndk_gcvB       CAACGGCTCTGTTGCTCATTATAATCCGCGCCATCTCGTACGCTGGTACAGACAACAACAGAACAATTTACAGAGGTAAAAATGGCTATTGAACGTACTTTTTCCATTATCAAACC
```

```
>ompA_RseX      GTGAAGGATTTAACCGTGTTATCTCGTTGGAGATATTCATGGCGTATTTTGGATGATAACGAGGCGCAAAAAATGAAAAAGACAGCTATCGCGATTGCAGTGGCACTGGCTGGTT
>ompA_RseX      GTGAAGGATTTAACCGTGTTATCTCGTTGGAGATATTCATGGCGTATTTTGGATGATAACGAGGCGCAAAAAATGAAAAAGACAGCTATCGCGATTGCAGTGGCACTGGCTGGTT
>ompA_RseX      GTGAAGGATTTAACCGTGTTATCTCGTTGGAGATATTCATGGCGTATTTTGGATGATAACGAGGCGCAAAAAATGAAAAAGACAGCTATCGCGATTGCAGTGGCACTGGCTGGTT
>ompC_MicC      GAGAATGGACTTGCCGACTGATTAATGAGGGTTAATCAGTATGCAGTGGCATAAAAAAGCAAATAAAGGCATATAACAGAGGGTTAATAACATGAAAGTTAAAGTACTGTCCCTCCTGGTCCCAGCT
>ompC_MicC      GAGAATGGACTTGCCGACTGATTAATGAGGGTTAATCAGTATGCAGTGGCATAAAAAAGCAAATAAAGGCATATAACAGAGGGTTAATAACATGAAAGTTAAAGTACTGTCCCTCCTGGTCCCAGCT
>ompC_MicC      GAGAATGGACTTGCCGACTGATTAATGAGGGTTAATCAGTATGCAGTGGCATAAAAAAGCAAATAAAGGCATATAACAGAGGGTTAATAACATGAAAGTTAAAGTACTGTCCCTCCTGGTCCCAGCT
>ompC_MicC      GAGAATGGACTTGCCGACTGATTAATGAGGGTTAATCAGTATGCAGTGGCATAAAAAAGCAAATAAAGGCATATAACAGAGGGTTAATAACATGAAAGTTAAAGTACTGTCCCTCCTGGTCCCAGCT
>ompC_RseX      TTGCCGACTGATTAATGAGGGTTAATCAGTATGCAGTGGCATAAAAAAGCAAATAAAGGCATATAACAGAGGGTTAATAACATGAAAGTTAAAGTACTGTCCCTCCTGGTCCCAGCTCTGCTGGTAGCAGG
>ompC_RseX      TTGCCGACTGATTAATGAGGGTTAATCAGTATGCAGTGGCATAAAAAAGCAAATAAAGGCATATAACAGAGGGTTAATAACATGAAAGTTAAAGTACTGTCCCTCCTGGTCCCAGCTCTGCTGGTAGCAGG
>ompC_RseX      TTGCCGACTGATTAATGAGGGTTAATCAGTATGCAGTGGCATAAAAAAGCAAATAAAGGCATATAACAGAGGGTTAATAACATGAAAGTTAAAGTACTGTCCCTCCTGGTCCCAGCTCTGCTGGTAGCAGG
>ompC_RseX      TTGCCGACTGATTAATGAGGGTTAATCAGTATGCAGTGGCATAAAAAAGCAAATAAAGGCATATAACAGAGGGTTAATAACATGAAAGTTAAAGTACTGTCCCTCCTGGTCCCAGCTCTGCTGGTAGCAGG
>ompC_RybB      TGCATTTTTGGGGAGAATGGACTTGCCGACTGATTAATGAGGGTTAATCAGTATGCAGTGGCATAAAAAAGCAAATAAAGGCATATAACAGAGGGTTAATAACATGAAAGTTAAAGTACTGTCCCTCCTGGTCCCAGCTCTGCTGGTAGC
>ompC_RybB      TGCATTTTTGGGGAGAATGGACTTGCCGACTGATTAATGAGGGTTAATCAGTATGCAGTGGCATAAAAAAGCAAATAAAGGCATATAACAGAGGGTTAATAACATGAAAGTTAAAGTACTGTCCCTCCTGGTCCCAGCTCTGCTGGTAGC
>ompC_RybB      TGCATTTTTGGGGAGAATGGACTTGCCGACTGATTAATGAGGGTTAATCAGTATGCAGTGGCATAAAAAAGCAAATAAAGGCATATAACAGAGGGTTAATAACATGAAAGTTAAAGTACTGTCCCTCCTGGTCCCAGCTCTGCTGGTAGC
>ompC_RybB      TGCATTTTTGGGGAGAATGGACTTGCCGACTGATTAATGAGGGTTAATCAGTATGCAGTGGCATAAAAAAGCAAATAAAGGCATATAACAGAGGGTTAATAACATGAAAGTTAAAGTACTGTCCCTCCTGGTCCCAGCTCTGCTGGTAGC
>ompF_MicF      TTTATTGACAGAAATTATTGACGGCAGTGGCAGGTGTCATAAAAAAAACCATGAGGGTAATAAATAATGATGAAGCGCAATATTCTGGCAGTGATCGTCCCTGCTCTGTTAGTAGCAGGTACTGCAA
>ompF_MicF      TTTATTGACAGAAATTATTGACGGCAGTGGCAGGTGTCATAAAAAAAACCATGAGGGTAATAAATAATGATGAAGCGCAATATTCTGGCAGTGATCGTCCCTGCTCTGTTAGTAGCAGGTACTGCAA
>ompF_MicF      TTTATTGACAGAAATTATTGACGGCAGTGGCAGGTGTCATAAAAAAAACCATGAGGGTAATAAATAATGATGAAGCGCAATATTCTGGCAGTGATCGTCCCTGCTCTGTTAGTAGCAGGTACTGCAA
>ompW_RybB      TCATTTGTTACTCCAATGTAGGTATATTCGTCACGTTTTTATAACCATAACGACGGAGCGGATATGAAAAAGTTAACAGTGGCGGCTTTGGCAGTAACAACTCTTCTCTCTGGCAGTGCCTTTGCGCATGAAGC
>phoP_MicA      GTTTACCCCCATAACCACATAATCGCGTTACACTATTTTAATAATTAAGACAGGGAGAAATAAAAATGCGCGTACTGGTTGTTGAAGACAATGCGTTGTTACGTCACCACCTTAAAGTTCAGAT
>phoP_MicA      GTTTACCCCCATAACCACATAATCGCGTTACACTATTTTAATAATTAAGACAGGGAGAAATAAAAATGCGCGTACTGGTTGTTGAAGACAATGCGTTGTTACGTCACCACCTTAAAGTTCAGAT
>ptsG_SgrS      TGTACACGGCGAGGCTCTCCCCCCTTGCCACGCGTGAGAACGTAAAAAAAGCACCCATACTCAGGAGCACTCTCAATTATGTTTAAGAATGCATTTGCTAACCTGCAAAAGGTCGGTAAATCGCTGATGCTGC
>ptsG_SgrS      TGTACACGGCGAGGCTCTCCCCCCTTGCCACGCGTGAGAACGTAAAAAAAGCACCCATACTCAGGAGCACTCTCAATTATGTTTAAGAATGCATTTGCTAACCTGCAAAAGGTCGGTAAATCGCTGATGCTGC
>rpoS_DsrA      AAATAGCAACCATGGGTAGCACCGGAACCAGTTCAACACGCTTGCATTTTGAAATTCGTTACAAGGGGAAATCCGTAAACCCGCTGCGTTATTTGCCGCAGCGATAAATCGGCGGAACCAGGC
>serA_gcvB      CAAACGTTCATATTGCCGCAATATTATTTTTTGATATGTTGAAAGGCGGATGCAAATCCGCACACAACATTTCAAAGACAGGATTGGGTAAATGGCAAAGGTATCGCTGGAGAAAGACAAGATT
>shiA_RyhB      TCAGCCATAAAATCATTTGCAGATGCGCTCTGTCGAGGTAGTCTCATAAGGTTCGTTTATAGATCGACGCAATGTGAGTTACCTTTTCCATACTAATTATAAAAAGACAGTACAAACAGGATCATTATGGACTCCACGCTCATCTCCACT
>shiA_RyhB      TCAGCCATAAAATCATTTGCAGATGCGCTCTGTCGAGGTAGTCTCATAAGGTTCGTTTATAGATCGACGCAATGTGAGTTACCTTTTCCATACTAATTATAAAAAGACAGTACAAACAGGATCATTATGGACTCCACGCTCATCTCCACT
>sodB_FnrS      CACCTTTTAATTTGCTACCCTATCCATACGCACAATAAGGCTATTGTACGTATGCAAATTAATAATAAAGGAGAGTAGCAATGTCATTCGAATTACCTGCACTACCATATGCTAAAGATGCTCTGGCACCGCACA
>sodB_FnrS      CACCTTTTAATTTGCTACCCTATCCATACGCACAATAAGGCTATTGTACGTATGCAAATTAATAATAAAGGAGAGTAGCAATGTCATTCGAATTACCTGCACTACCATATGCTAAAGATGCTCTGGCACCGCACA
>sodB_FnrS      CACCTTTTAATTTGCTACCCTATCCATACGCACAATAAGGCTATTGTACGTATGCAAATTAATAATAAAGGAGAGTAGCAATGTCATTCGAATTACCTGCACTACCATATGCTAAAGATGCTCTGGCACCGCACA
>sodB_FnrS      CACCTTTTAATTTGCTACCCTATCCATACGCACAATAAGGCTATTGTACGTATGCAAATTAATAATAAAGGAGAGTAGCAATGTCATTCGAATTACCTGCACTACCATATGCTAAAGATGCTCTGGCACCGCACA
>sodB_RyhB      GCTACCCTATCCATACGCACAATAAGGCTATTGTACGTATGCAAATTAATAATAAAGGAGAGTAGCAATGTCATTCGAATTACCTGCACTACCATATGCTAAAGATGCTCTGGCACCGCACATTTCT
>sodB_RyhB      GCTACCCTATCCATACGCACAATAAGGCTATTGTACGTATGCAAATTAATAATAAAGGAGAGTAGCAATGTCATTCGAATTACCTGCACTACCATATGCTAAAGATGCTCTGGCACCGCACATTTCT
>sodB_RyhB      GCTACCCTATCCATACGCACAATAAGGCTATTGTACGTATGCAAATTAATAATAAAGGAGAGTAGCAATGTCATTCGAATTACCTGCACTACCATATGCTAAAGATGCTCTGGCACCGCACATTTCT
>sodB_RyhB      GCTACCCTATCCATACGCACAATAAGGCTATTGTACGTATGCAAATTAATAATAAAGGAGAGTAGCAATGTCATTCGAATTACCTGCACTACCATATGCTAAAGATGCTCTGGCACCGCACATTTCT
>srlA_spf       CTTTCAATCTGATTAGATTAGGTTGCCGTTTGGTAATAAAACAATAAATCCTGAAGGAGAGAACAATGATAGAAACCATTACTCATGGTGCAGAGTGGTTTATCGGGCTGTTCCA
>srlA_spf       CTTTCAATCTGATTAGATTAGGTTGCCGTTTGGTAATAAAACAATAAATCCTGAAGGAGAGAACAATGATAGAAACCATTACTCATGGTGCAGAGTGGTTTATCGGGCTGTTCCA
>srlA_spf       CTTTCAATCTGATTAGATTAGGTTGCCGTTTGGTAATAAAACAATAAATCCTGAAGGAGAGAACAATGATAGAAACCATTACTCATGGTGCAGAGTGGTTTATCGGGCTGTTCCA
>tppB_gcvB      AAAGCACCCCCGTTAATATGGGATGTAAAAAAAGAGGTAAAAGTGTCCACTGCAAACCAAAAACCAACTGAAAGCGTCAGTTTGAACGCTTTCAAACAACCGAAGGCGTTCTATCTCATC
>tppB_gcvB      AAAGCACCCCCGTTAATATGGGATGTAAAAAAAGAGGTAAAAGTGTCCACTGCAAACCAAAAACCAACTGAAAGCGTCAGTTTGAACGCTTTCAAACAACCGAAGGCGTTCTATCTCATC
>tppB_gcvB      AAAGCACCCCCGTTAATATGGGATGTAAAAAAAGAGGTAAAAGTGTCCACTGCAAACCAAAAACCAACTGAAAGCGTCAGTTTGAACGCTTTCAAACAACCGAAGGCGTTCTATCTCATC
>tppB_gcvB      AAAGCACCCCCGTTAATATGGGATGTAAAAAAAGAGGTAAAAGTGTCCACTGCAAACCAAAAACCAACTGAAAGCGTCAGTTTGAACGCTTTCAAACAACCGAAGGCGTTCTATCTCATC
>tppB_gcvB      AAAGCACCCCCGTTAATATGGGATGTAAAAAAAGAGGTAAAAGTGTCCACTGCAAACCAAAAACCAACTGAAAGCGTCAGTTTGAACGCTTTCAAACAACCGAAGGCGTTCTATCTCATC
>xylF_spf       TGATTGTTACTTATTAAAGCTGTCCTCTAACTACAGAAGGCCCTACACCATGAAAATAAAGAACATTCTACTCACCCTTTGCACCTCACTCCTGCTTACCAACGTTGCTGCACACGCCAAAGAAGTCAAAATAGGTATG
>xylF_spf       TGATTGTTACTTATTAAAGCTGTCCTCTAACTACAGAAGGCCCTACACCATGAAAATAAAGAACATTCTACTCACCCTTTGCACCTCACTCCTGCTTACCAACGTTGCTGCACACGCCAAAGAAGTCAAAATAGGTATG
>xylF_spf       TGATTGTTACTTATTAAAGCTGTCCTCTAACTACAGAAGGCCCTACACCATGAAAATAAAGAACATTCTACTCACCCTTTGCACCTCACTCCTGCTTACCAACGTTGCTGCACACGCCAAAGAAGTCAAAATAGGTATG
>xylF_spf       TGATTGTTACTTATTAAAGCTGTCCTCTAACTACAGAAGGCCCTACACCATGAAAATAAAGAACATTCTACTCACCCTTTGCACCTCACTCCTGCTTACCAACGTTGCTGCACACGCCAAAGAAGTCAAAATAGGTATG
>yaeC_gcvB      TCAGTTCGCAGGCGACCGCATCGTCCGGGCTGTCACTCGCAAGTAACGTTCAACACAACATAAATAATTGAAGAAGGAATAAGGTATGGCGTTCAAATTCAAAACCTTTGCGGCAGTGGG
>yaeC_gcvB      TCAGTTCGCAGGCGACCGCATCGTCCGGGCTGTCACTCGCAAGTAACGTTCAACACAACATAAATAATTGAAGAAGGAATAAGGTATGGCGTTCAAATTCAAAACCTTTGCGGCAGTGGG
>yaeC_gcvB      TCAGTTCGCAGGCGACCGCATCGTCCGGGCTGTCACTCGCAAGTAACGTTCAACACAACATAAATAATTGAAGAAGGAATAAGGTATGGCGTTCAAATTCAAAACCTTTGCGGCAGTGGG
>yaeC_gcvB      TCAGTTCGCAGGCGACCGCATCGTCCGGGCTGTCACTCGCAAGTAACGTTCAACACAACATAAATAATTGAAGAAGGAATAAGGTATGGCGTTCAAATTCAAAACCTTTGCGGCAGTGGG
>yaeC_gcvB      TCAGTTCGCAGGCGACCGCATCGTCCGGGCTGTCACTCGCAAGTAACGTTCAACACAACATAAATAATTGAAGAAGGAATAAGGTATGGCGTTCAAATTCAAAACCTTTGCGGCAGTGGG
>ybdH_gcvB      TGCTAGTATTTGGCAATCAAGACGTTTAGATGTCTAAATAAAACAATAAGGACAACACAACATGCCTCACAATCCTATCCGCGTGGTCGTCGGCCCGGCTAACTACTTTTCAC
>ybdH_gcvB      TGCTAGTATTTGGCAATCAAGACGTTTAGATGTCTAAATAAAACAATAAGGACAACACAACATGCCTCACAATCCTATCCGCGTGGTCGTCGGCCCGGCTAACTACTTTTCAC
>ybdH_gcvB      TGCTAGTATTTGGCAATCAAGACGTTTAGATGTCTAAATAAAACAATAAGGACAACACAACATGCCTCACAATCCTATCCGCGTGGTCGTCGGCCCGGCTAACTACTTTTCAC
>ybdH_gcvB      TGCTAGTATTTGGCAATCAAGACGTTTAGATGTCTAAATAAAACAATAAGGACAACACAACATGCCTCACAATCCTATCCGCGTGGTCGTCGGCCCGGCTAACTACTTTTCAC
>yqaE_CyaR      TAAGAGATAAAATCTCTTTTTAAACAATAAGTAATTTTCTTATAGGGAGTACATATGGGTTTCTGGAGAATCGTCATCACCATCATTCTGCCGCCGCTCGGCGTGCTGCTCGGTAAAGGGT
>yqaE_CyaR      TAAGAGATAAAATCTCTTTTTAAACAATAAGTAATTTTCTTATAGGGAGTACATATGGGTTTCTGGAGAATCGTCATCACCATCATTCTGCCGCCGCTCGGCGTGCTGCTCGGTAAAGGGT
```

ARN5_m1

```
>cycA_GcvB      ACAGATTCGTCGTATCATAGACTGACTAAAGGCCGTAGAGCCTGAACAACACAGACAGGTACAGGAAGAAAAAAACATGGTAGATCAGGTAAAAGTCGTTGCCGATGATCAGGCTCCGGCTGAACAGTCGCTAC
>cycA_GcvB      ACAGATTCGTCGTATCATAGACTGACTAAAGGCCGTAGAGCCTGAACAACACAGACAGGTACAGGAAGAAAAAAACATGGTAGATCAGGTAAAAGTCGTTGCCGATGATCAGGCTCCGGCTGAACAGTCGCTAC
>lrp_micF       GATGTTTCAGGGTCAGACAGGAGTAGGGAAGGAATACAGAGAGACAATAATAATGGTAGATAGCAAGAAGCGCCCTGGCAAAGATCTCGACCGTATCGATCGTAACATTCTTAA
>lrp_micF       GATGTTTCAGGGTCAGACAGGAGTAGGGAAGGAATACAGAGAGACAATAATAATGGTAGATAGCAAGAAGCGCCCTGGCAAAGATCTCGACCGTATCGATCGTAACATTCTTAA
>metE_FnrS      TGAATAAACTTGCCGCCTTCCCTAAATTCAAAATCCATAGGATTTACATATAATTAGAGGAAGAAAAATGACAATATTGAATCACACCCTCGGTTTCCCTCGCGTTGGCCTGCGTCGCGAGCTGAAAAAAG
>metE_FnrS      TGAATAAACTTGCCGCCTTCCCTAAATTCAAAATCCATAGGATTTACATATAATTAGAGGAAGAAAAATGACAATATTGAATCACACCCTCGGTTTCCCTCGCGTTGGCCTGCGTCGCGAGCTGAAAAAAG
>metE_FnrS      TGAATAAACTTGCCGCCTTCCCTAAATTCAAAATCCATAGGATTTACATATAATTAGAGGAAGAAAAATGACAATATTGAATCACACCCTCGGTTTCCCTCGCGTTGGCCTGCGTCGCGAGCTGAAAAAAG
>metE_FnrS      TGAATAAACTTGCCGCCTTCCCTAAATTCAAAATCCATAGGATTTACATATAATTAGAGGAAGAAAAATGACAATATTGAATCACACCCTCGGTTTCCCTCGCGTTGGCCTGCGTCGCGAGCTGAAAAAAG
>ompA_RseX      GTGAAGGATTTAACCGTGTTATCTCGTTGGAGATATTCATGGCGTATTTTGGATGATAACGAGGCGCAAAAAATGAAAAAGACAGCTATCGCGATTGCAGTGGCACTGGCTGGTT
```

Figure S2 cont.

```
>ompC_MicC     GAGAATGGACTTGCCGACTGATTAATGAGGGTTAATCAGTATGCAGTGGCATAAAAAAGCAAATAAAGGCATATAACAGAGGGTTAATAACATGAAAGTTAAAGTACTGTCCCTCCTGGTCCCAGCT
>ompC_RseX     TTGCCGACTGATTAATGAGGGTTAATCAGTATGCAGTGGCATAAAAAAGCAAATAAAGGCATATAACAGAGGGTTAATAACATGAAAGTTAAAGTACTGTCCCTCCTGGTCCCAGCTCTGCTGGTAGCAGG
>ompC_RybB     TGCATTTTTGGGGAGAATGGACTTGCCGACTGATTAATGAGGGTTAATCAGTATGCAGTGGCATAAAAAAGCAAATAAAGGCATATAACAGAGGGTTAATAACATGAAAGTTAAAGTACTGTCCCTCCTGGTCCCAGCTCTGCTGGTAGC
>phoP_MicA     GTTTACCCCCATAACCACATAATCGCGTTACACTATTTTAATAATTAAGACAGGGAGAAATAAAAATGCGCGTACTGGTTGTTGAAGACAATGCGTTGTTACGTCACCACCTTAAAGTTCAGAT
>sodB_FnrS     CACCTTTTAATTTGCTACCCTATCCATACGCACAATAAGGCTATTGTACGTATGCAAATTAATAATAAAGGAGAGTAGCAATGTCATTCGAATTACCTGCACTACCATATGCTAAAGATGCTCTGGCACCGCACA
>sodB_RyhB     GCTACCCTATCCATACGCACAATAAGGCTATTGTACGTATGCAAATTAATAATAAAGGAGAGTAGCAATGTCATTCGAATTACCTGCACTACCATATGCTAAAGATGCTCTGGCACCGCACATTTCT
>srlA_spf      CTTTCAATCTGATTAGATTAGGTTGCCGTTTGGTAATAAAACAATAAATCCTGAAGGAGAGAACAATGATAGAAACCATTACTCATGGTGCAGAGTGGTTTATCGGGCTGTTCCA
>tppB_gcvB     AAAGCACCCCCGTTAATATGGGATGTAAAAAAAGAGGTAAAAGTGTCCACTGCAAACCAAAAACCAACTGAAAGCGTCAGTTTGAACGCTTTCAAACAACCGAAGGCGTTCTATCTCATC
>tppB_gcvB     AAAGCACCCCCGTTAATATGGGATGTAAAAAAAGAGGTAAAAGTGTCCACTGCAAACCAAAAACCAACTGAAAGCGTCAGTTTGAACGCTTTCAAACAACCGAAGGCGTTCTATCTCATC
>xylF_spf      TGATTGTTACTTATTAAAGCTGTCCTCTAACTACAGAAGGCCCTACACCATGAAAATAAAGAACATTCTACTCACCCTTTGCACCTCACTCCTGCTTACCAACGTTGCTGCACACGCCAAAGAAGTCAAAATAGGTATG
>yaeC_gcvB     TCAGTTCGCAGGCGACCGCATCGTCCGGGCTGTCACTCGCAAGTAACGTTCAACACAACATAAATAATTGAAGAAGGAATAAGGTATGGCGTTCAAATTCAAAACCTTTGCGGCAGTGGG
>yaeC_gcvB     TCAGTTCGCAGGCGACCGCATCGTCCGGGCTGTCACTCGCAAGTAACGTTCAACACAACATAAATAATTGAAGAAGGAATAAGGTATGGCGTTCAAATTCAAAACCTTTGCGGCAGTGGG
>yaeC_gcvB     TCAGTTCGCAGGCGACCGCATCGTCCGGGCTGTCACTCGCAAGTAACGTTCAACACAACATAAATAATTGAAGAAGGAATAAGGTATGGCGTTCAAATTCAAAACCTTTGCGGCAGTGGG
>ybdH_gcvB     TGCTAGTATTTGGCAATCAAGACGTTTAGATGTCTAAATAAAACAATAAGGACAACACAACATGCCTCACAATCCTATCCGCGTGGTCGTCGGCCCGGCTAACTACTTTTCAC
>ybdH_gcvB     TGCTAGTATTTGGCAATCAAGACGTTTAGATGTCTAAATAAAACAATAAGGACAACACAACATGCCTCACAATCCTATCCGCGTGGTCGTCGGCCCGGCTAACTACTTTTCAC
```

ARN5_m2

```
>cycA_GcvB     ACAGATTCGTCGTATCATAGACTGACTAAAGGCCGTAGAGCCTGAACAACACAGACAGGTACAGGAAGAAAAAAACATGGTAGATCAGGTAAAAGTCGTTGCCGATGATCAGGCTCCGGCTGAACAGTCGCTAC
>cycA_GcvB     ACAGATTCGTCGTATCATAGACTGACTAAAGGCCGTAGAGCCTGAACAACACAGACAGGTACAGGAAGAAAAAAACATGGTAGATCAGGTAAAAGTCGTTGCCGATGATCAGGCTCCGGCTGAACAGTCGCTAC
>cycA_GcvB     ACAGATTCGTCGTATCATAGACTGACTAAAGGCCGTAGAGCCTGAACAACACAGACAGGTACAGGAAGAAAAAAACATGGTAGATCAGGTAAAAGTCGTTGCCGATGATCAGGCTCCGGCTGAACAGTCGCTAC
>cycA_GcvB     ACAGATTCGTCGTATCATAGACTGACTAAAGGCCGTAGAGCCTGAACAACACAGACAGGTACAGGAAGAAAAAAACATGGTAGATCAGGTAAAAGTCGTTGCCGATGATCAGGCTCCGGCTGAACAGTCGCTAC
>cycA_GcvB     ACAGATTCGTCGTATCATAGACTGACTAAAGGCCGTAGAGCCTGAACAACACAGACAGGTACAGGAAGAAAAAAACATGGTAGATCAGGTAAAAGTCGTTGCCGATGATCAGGCTCCGGCTGAACAGTCGCTAC
>cycA_GcvB     ACAGATTCGTCGTATCATAGACTGACTAAAGGCCGTAGAGCCTGAACAACACAGACAGGTACAGGAAGAAAAAAACATGGTAGATCAGGTAAAAGTCGTTGCCGATGATCAGGCTCCGGCTGAACAGTCGCTAC
>cycA_GcvB     ACAGATTCGTCGTATCATAGACTGACTAAAGGCCGTAGAGCCTGAACAACACAGACAGGTACAGGAAGAAAAAAACATGGTAGATCAGGTAAAAGTCGTTGCCGATGATCAGGCTCCGGCTGAACAGTCGCTAC
>cycA_GcvB     ACAGATTCGTCGTATCATAGACTGACTAAAGGCCGTAGAGCCTGAACAACACAGACAGGTACAGGAAGAAAAAAACATGGTAGATCAGGTAAAAGTCGTTGCCGATGATCAGGCTCCGGCTGAACAGTCGCTAC
>fhlA_OxyS     TAAATCTCCTATAGTTAGTCAATGACCTTTTGCACCGCTTTGCGGTGCTTTCCTGGAAGAACAAAATGTCATATACACCGATGAGTGATCTCGGACAGCAAGGGTTG
>fucI_spf      TTTATCTTTGCCCGTTTCCGTTCTCAAACGGCAACTAACTGAACATATTTTCCGAATAAAGTGAGGAATCTGTAATGAACAAAATCAGCTTACCGAAAATTGGTATCCGCCCGGTTATTGACGGTCGTCGCATGGGTGTTCGTGAGTCGCTTGAAGAA
>fucI_spf      TTTATCTTTGCCCGTTTCCGTTCTCAAACGGCAACTAACTGAACATATTTTCCGAATAAAGTGAGGAATCTGTAATGAACAAAATCAGCTTACCGAAAATTGGTATCCGCCCGGTTATTGACGGTCGTCGCATGGGTGTTCGTGAGTCGCTTGAAGAA
>fur_RyhB      TGCCACGTTTTTATTAACAATATTTGCCAGGGACTTGTGGTTTTCATTTAGGCGTGGCAATTCTATAATGATACGCATTATCTCAAGAGCAAATTCTGTCACTTCTTCTAATGAAGTGAACCGCTTAGTAACAGGACAGATTCCGCATGA
>gdhA_gcvB     TGCTTACCGTCACATTCTTGATGGTATAGTCGAAAACTGCAAAAGCACATGACATAAACAACATAAGCACAATCGTATTAATATATAAGGGTTTTATATCTATGGATCAGACATATTCTCTGGAGTCA
>gdhA_gcvB     TGCTTACCGTCACATTCTTGATGGTATAGTCGAAAACTGCAAAAGCACATGACATAAACAACATAAGCACAATCGTATTAATATATAAGGGTTTTATATCTATGGATCAGACATATTCTCTGGAGTCA
>gdhA_gcvB     TGCTTACCGTCACATTCTTGATGGTATAGTCGAAAACTGCAAAAGCACATGACATAAACAACATAAGCACAATCGTATTAATATATAAGGGTTTTATATCTATGGATCAGACATATTCTCTGGAGTCA
>gdhA_gcvB     TGCTTACCGTCACATTCTTGATGGTATAGTCGAAAACTGCAAAAGCACATGACATAAACAACATAAGCACAATCGTATTAATATATAAGGGTTTTATATCTATGGATCAGACATATTCTCTGGAGTCA
>glmS_GlmZ     AAAATAACCCCACTCTCTACAAGGCTCGGGGCGCCCGAAAAAACGGGCATACAGGTTGACCGACAACGATATAAATCGGAATCAAAAACTATGTGTGGAATTGTTGGCGCGATCGCGCA
>lrp_micF      GATGTTTCAGGGTCAGACAGGAGTAGGGAAGGAATACAGAGAGACAATAATAATGGTAGATAGCAAGAAGCGCCCTGGCAAAGATCTCGACCGTATCGATCGTAACATTCTTAA
>lrp_micF      GATGTTTCAGGGTCAGACAGGAGTAGGGAAGGAATACAGAGAGACAATAATAATGGTAGATAGCAAGAAGCGCCCTGGCAAAGATCTCGACCGTATCGATCGTAACATTCTTAA
>lrp_micF      GATGTTTCAGGGTCAGACAGGAGTAGGGAAGGAATACAGAGAGACAATAATAATGGTAGATAGCAAGAAGCGCCCTGGCAAAGATCTCGACCGTATCGATCGTAACATTCTTAA
>lrp_micF      GATGTTTCAGGGTCAGACAGGAGTAGGGAAGGAATACAGAGAGACAATAATAATGGTAGATAGCAAGAAGCGCCCTGGCAAAGATCTCGACCGTATCGATCGTAACATTCTTAA
>lrp_micF      GATGTTTCAGGGTCAGACAGGAGTAGGGAAGGAATACAGAGAGACAATAATAATGGTAGATAGCAAGAAGCGCCCTGGCAAAGATCTCGACCGTATCGATCGTAACATTCTTAA
>lrp_micF      GATGTTTCAGGGTCAGACAGGAGTAGGGAAGGAATACAGAGAGACAATAATAATGGTAGATAGCAAGAAGCGCCCTGGCAAAGATCTCGACCGTATCGATCGTAACATTCTTAA
>lrp_micF      GATGTTTCAGGGTCAGACAGGAGTAGGGAAGGAATACAGAGAGACAATAATAATGGTAGATAGCAAGAAGCGCCCTGGCAAAGATCTCGACCGTATCGATCGTAACATTCTTAA
>lrp_micF      GATGTTTCAGGGTCAGACAGGAGTAGGGAAGGAATACAGAGAGACAATAATAATGGTAGATAGCAAGAAGCGCCCTGGCAAAGATCTCGACCGTATCGATCGTAACATTCTTAA
>lrp_micF      GATGTTTCAGGGTCAGACAGGAGTAGGGAAGGAATACAGAGAGACAATAATAATGGTAGATAGCAAGAAGCGCCCTGGCAAAGATCTCGACCGTATCGATCGTAACATTCTTAA
>luxS_CyaR     TCAGACTCGCCTGGGAAGAAAGAGTTCAGAAAATTTTTAAAAAAATTACCGGAGGTGGCTAAATGCCGTTGTTAGATAGCTTCACAGTCGATCATACCCGGATGGAAGCGCCTGCA
>metE_FnrS     TGAATAAACTTGCCGCCTTCCCTAAATTCAAAATCCATAGGATTTACATATAATTAGAGGAAGAAAAATGACAATATTGAATCACACCCTCGGTTTCCCTCGCGTTGGCCTGCGTCGCGAGCTGAAAAAAG
>metE_FnrS     TGAATAAACTTGCCGCCTTCCCTAAATTCAAAATCCATAGGATTTACATATAATTAGAGGAAGAAAAATGACAATATTGAATCACACCCTCGGTTTCCCTCGCGTTGGCCTGCGTCGCGAGCTGAAAAAAG
>metE_FnrS     TGAATAAACTTGCCGCCTTCCCTAAATTCAAAATCCATAGGATTTACATATAATTAGAGGAAGAAAAATGACAATATTGAATCACACCCTCGGTTTCCCTCGCGTTGGCCTGCGTCGCGAGCTGAAAAAAG
>metE_FnrS     TGAATAAACTTGCCGCCTTCCCTAAATTCAAAATCCATAGGATTTACATATAATTAGAGGAAGAAAAATGACAATATTGAATCACACCCTCGGTTTCCCTCGCGTTGGCCTGCGTCGCGAGCTGAAAAAAG
>metE_FnrS     TGAATAAACTTGCCGCCTTCCCTAAATTCAAAATCCATAGGATTTACATATAATTAGAGGAAGAAAAATGACAATATTGAATCACACCCTCGGTTTCCCTCGCGTTGGCCTGCGTCGCGAGCTGAAAAAAG
>metE_FnrS     TGAATAAACTTGCCGCCTTCCCTAAATTCAAAATCCATAGGATTTACATATAATTAGAGGAAGAAAAATGACAATATTGAATCACACCCTCGGTTTCCCTCGCGTTGGCCTGCGTCGCGAGCTGAAAAAAG
>metE_FnrS     TGAATAAACTTGCCGCCTTCCCTAAATTCAAAATCCATAGGATTTACATATAATTAGAGGAAGAAAAATGACAATATTGAATCACACCCTCGGTTTCCCTCGCGTTGGCCTGCGTCGCGAGCTGAAAAAAG
>nadE_CyaR     CGTTTCAGTAGCAACGGGTTAGCTTTAAGGAAGTTTTGTCTTTTCTGTCTGGAGGGGTTCAATGACATTGCAACAACAAATAATAAAGGCGCTGGGCGCAAAACCGCAGATTAAT
>nadE_CyaR     CGTTTCAGTAGCAACGGGTTAGCTTTAAGGAAGTTTTGTCTTTTCTGTCTGGAGGGGTTCAATGACATTGCAACAACAAATAATAAAGGCGCTGGGCGCAAAACCGCAGATTAAT
>nadE_CyaR     CGTTTCAGTAGCAACGGGTTAGCTTTAAGGAAGTTTTGTCTTTTCTGTCTGGAGGGGTTCAATGACATTGCAACAACAAATAATAAAGGCGCTGGGCGCAAAACCGCAGATTAAT
>nadE_CyaR     CGTTTCAGTAGCAACGGGTTAGCTTTAAGGAAGTTTTGTCTTTTCTGTCTGGAGGGGTTCAATGACATTGCAACAACAAATAATAAAGGCGCTGGGCGCAAAACCGCAGATTAAT
>nadE_CyaR     CGTTTCAGTAGCAACGGGTTAGCTTTAAGGAAGTTTTGTCTTTTCTGTCTGGAGGGGTTCAATGACATTGCAACAACAAATAATAAAGGCGCTGGGCGCAAAACCGCAGATTAAT
>nadE_CyaR     CGTTTCAGTAGCAACGGGTTAGCTTTAAGGAAGTTTTGTCTTTTCTGTCTGGAGGGGTTCAATGACATTGCAACAACAAATAATAAAGGCGCTGGGCGCAAAACCGCAGATTAAT
>ndk_gcvB      CAACGGCTCTGTTGCTCATTATAATCCGCGCCATCTCGTACGCTGGTACAGACAACAACAGAACAATTTACAGAGGTAAAAATGGCTATTGAACGTACTTTTTCCATTATCAAACC
>ndk_gcvB      CAACGGCTCTGTTGCTCATTATAATCCGCGCCATCTCGTACGCTGGTACAGACAACAACAGAACAATTTACAGAGGTAAAAATGGCTATTGAACGTACTTTTTCCATTATCAAACC
>ndk_gcvB      CAACGGCTCTGTTGCTCATTATAATCCGCGCCATCTCGTACGCTGGTACAGACAACAACAGAACAATTTACAGAGGTAAAAATGGCTATTGAACGTACTTTTTCCATTATCAAACC
>ndk_gcvB      CAACGGCTCTGTTGCTCATTATAATCCGCGCCATCTCGTACGCTGGTACAGACAACAACAGAACAATTTACAGAGGTAAAAATGGCTATTGAACGTACTTTTTCCATTATCAAACC
>ndk_gcvB      CAACGGCTCTGTTGCTCATTATAATCCGCGCCATCTCGTACGCTGGTACAGACAACAACAGAACAATTTACAGAGGTAAAAATGGCTATTGAACGTACTTTTTCCATTATCAAACC
>ompA_RseX     GTGAAGGATTTAACCGTGTTATCTCGTTGGAGATATTCATGGCGTATTTTGGATGATAACGAGGCGCAAAAAATGAAAAAGACAGCTATCGCGATTGCAGTGGCACTGGCTGGTT
>ompA_RseX     GTGAAGGATTTAACCGTGTTATCTCGTTGGAGATATTCATGGCGTATTTTGGATGATAACGAGGCGCAAAAAATGAAAAAGACAGCTATCGCGATTGCAGTGGCACTGGCTGGTT
>ompA_RseX     GTGAAGGATTTAACCGTGTTATCTCGTTGGAGATATTCATGGCGTATTTTGGATGATAACGAGGCGCAAAAAATGAAAAAGACAGCTATCGCGATTGCAGTGGCACTGGCTGGTT
>ompA_RseX     GTGAAGGATTTAACCGTGTTATCTCGTTGGAGATATTCATGGCGTATTTTGGATGATAACGAGGCGCAAAAAATGAAAAAGACAGCTATCGCGATTGCAGTGGCACTGGCTGGTT
>ompA_RseX     GTGAAGGATTTAACCGTGTTATCTCGTTGGAGATATTCATGGCGTATTTTGGATGATAACGAGGCGCAAAAAATGAAAAAGACAGCTATCGCGATTGCAGTGGCACTGGCTGGTT
>ompA_RseX     GTGAAGGATTTAACCGTGTTATCTCGTTGGAGATATTCATGGCGTATTTTGGATGATAACGAGGCGCAAAAAATGAAAAAGACAGCTATCGCGATTGCAGTGGCACTGGCTGGTT
>ompA_RseX     GTGAAGGATTTAACCGTGTTATCTCGTTGGAGATATTCATGGCGTATTTTGGATGATAACGAGGCGCAAAAAATGAAAAAGACAGCTATCGCGATTGCAGTGGCACTGGCTGGTT
```

```
>ompC_MicC     GAGAATGGACTTGCCGACTGATTAATGAGGGTTAATCAGTATGCAGTGGCATAAAAAAGCAAATAAAGGCATATAACAGAGGGTTAATAACATGAAAGTTAAAGTACTGTCCCTCCTGGTCCCAGCT
>ompC_MicC     GAGAATGGACTTGCCGACTGATTAATGAGGGTTAATCAGTATGCAGTGGCATAAAAAAGCAAATAAAGGCATATAACAGAGGGTTAATAACATGAAAGTTAAAGTACTGTCCCTCCTGGTCCCAGCT
>ompC_MicC     GAGAATGGACTTGCCGACTGATTAATGAGGGTTAATCAGTATGCAGTGGCATAAAAAAGCAAATAAAGGCATATAACAGAGGGTTAATAACATGAAAGTTAAAGTACTGTCCCTCCTGGTCCCAGCT
>ompC_MicC     GAGAATGGACTTGCCGACTGATTAATGAGGGTTAATCAGTATGCAGTGGCATAAAAAAGCAAATAAAGGCATATAACAGAGGGTTAATAACATGAAAGTTAAAGTACTGTCCCTCCTGGTCCCAGCT
>ompC_MicC     GAGAATGGACTTGCCGACTGATTAATGAGGGTTAATCAGTATGCAGTGGCATAAAAAAGCAAATAAAGGCATATAACAGAGGGTTAATAACATGAAAGTTAAAGTACTGTCCCTCCTGGTCCCAGCT
>ompC_MicC     GAGAATGGACTTGCCGACTGATTAATGAGGGTTAATCAGTATGCAGTGGCATAAAAAAGCAAATAAAGGCATATAACAGAGGGTTAATAACATGAAAGTTAAAGTACTGTCCCTCCTGGTCCCAGCT
>ompC_RseX     TTGCCGACTGATTAATGAGGGTTAATCAGTATGCAGTGGCATAAAAAAGCAAATAAAGGCATATAACAGAGGGTTAATAACATGAAAGTTAAAGTACTGTCCCTCCTGGTCCCAGCTCTGCTGGTAGCAGG
>ompC_RseX     TTGCCGACTGATTAATGAGGGTTAATCAGTATGCAGTGGCATAAAAAAGCAAATAAAGGCATATAACAGAGGGTTAATAACATGAAAGTTAAAGTACTGTCCCTCCTGGTCCCAGCTCTGCTGGTAGCAGG
>ompC_RseX     TTGCCGACTGATTAATGAGGGTTAATCAGTATGCAGTGGCATAAAAAAGCAAATAAAGGCATATAACAGAGGGTTAATAACATGAAAGTTAAAGTACTGTCCCTCCTGGTCCCAGCTCTGCTGGTAGCAGG
>ompC_RseX     TTGCCGACTGATTAATGAGGGTTAATCAGTATGCAGTGGCATAAAAAAGCAAATAAAGGCATATAACAGAGGGTTAATAACATGAAAGTTAAAGTACTGTCCCTCCTGGTCCCAGCTCTGCTGGTAGCAGG
>ompC_RseX     TTGCCGACTGATTAATGAGGGTTAATCAGTATGCAGTGGCATAAAAAAGCAAATAAAGGCATATAACAGAGGGTTAATAACATGAAAGTTAAAGTACTGTCCCTCCTGGTCCCAGCTCTGCTGGTAGCAGG
>ompC_RseX     TTGCCGACTGATTAATGAGGGTTAATCAGTATGCAGTGGCATAAAAAAGCAAATAAAGGCATATAACAGAGGGTTAATAACATGAAAGTTAAAGTACTGTCCCTCCTGGTCCCAGCTCTGCTGGTAGCAGG
>ompC_RybB     TGCATTTTTGGGGAGAATGGACTTGCCGACTGATTAATGAGGGTTAATCAGTATGCAGTGGCATAAAAAAGCAAATAAAGGCATATAACAGAGGGTTAATAACATGAAAGTTAAAGTACTGTCCCTCCTGGTCCCAGCTCTGCTGGTAGC
>ompC_RybB     TGCATTTTTGGGGAGAATGGACTTGCCGACTGATTAATGAGGGTTAATCAGTATGCAGTGGCATAAAAAAGCAAATAAAGGCATATAACAGAGGGTTAATAACATGAAAGTTAAAGTACTGTCCCTCCTGGTCCCAGCTCTGCTGGTAGC
>ompC_RybB     TGCATTTTTGGGGAGAATGGACTTGCCGACTGATTAATGAGGGTTAATCAGTATGCAGTGGCATAAAAAAGCAAATAAAGGCATATAACAGAGGGTTAATAACATGAAAGTTAAAGTACTGTCCCTCCTGGTCCCAGCTCTGCTGGTAGC
>ompC_RybB     TGCATTTTTGGGGAGAATGGACTTGCCGACTGATTAATGAGGGTTAATCAGTATGCAGTGGCATAAAAAAGCAAATAAAGGCATATAACAGAGGGTTAATAACATGAAAGTTAAAGTACTGTCCCTCCTGGTCCCAGCTCTGCTGGTAGC
>ompC_RybB     TGCATTTTTGGGGAGAATGGACTTGCCGACTGATTAATGAGGGTTAATCAGTATGCAGTGGCATAAAAAAGCAAATAAAGGCATATAACAGAGGGTTAATAACATGAAAGTTAAAGTACTGTCCCTCCTGGTCCCAGCTCTGCTGGTAGC
>ompC_RybB     TGCATTTTTGGGGAGAATGGACTTGCCGACTGATTAATGAGGGTTAATCAGTATGCAGTGGCATAAAAAAGCAAATAAAGGCATATAACAGAGGGTTAATAACATGAAAGTTAAAGTACTGTCCCTCCTGGTCCCAGCTCTGCTGGTAGC
>ompF_MicF     TTTATTGACAGAAATTATTGACGGCAGTGGCAGGTGTCATAAAAAAAACCATGAGGGTAATAAATAATGATGAAGCGCAATATTCTGGCAGTGATCGTCCCTGCTCTGTTAGTAGCAGGTACTGCAA
>ompF_MicF     TTTATTGACAGAAATTATTGACGGCAGTGGCAGGTGTCATAAAAAAAACCATGAGGGTAATAAATAATGATGAAGCGCAATATTCTGGCAGTGATCGTCCCTGCTCTGTTAGTAGCAGGTACTGCAA
>ompF_MicF     TTTATTGACAGAAATTATTGACGGCAGTGGCAGGTGTCATAAAAAAAACCATGAGGGTAATAAATAATGATGAAGCGCAATATTCTGGCAGTGATCGTCCCTGCTCTGTTAGTAGCAGGTACTGCAA
>ompF_MicF     TTTATTGACAGAAATTATTGACGGCAGTGGCAGGTGTCATAAAAAAAACCATGAGGGTAATAAATAATGATGAAGCGCAATATTCTGGCAGTGATCGTCCCTGCTCTGTTAGTAGCAGGTACTGCAA
>ompF_MicF     TTTATTGACAGAAATTATTGACGGCAGTGGCAGGTGTCATAAAAAAAACCATGAGGGTAATAAATAATGATGAAGCGCAATATTCTGGCAGTGATCGTCCCTGCTCTGTTAGTAGCAGGTACTGCAA
>ompF_MicF     TTTATTGACAGAAATTATTGACGGCAGTGGCAGGTGTCATAAAAAAAACCATGAGGGTAATAAATAATGATGAAGCGCAATATTCTGGCAGTGATCGTCCCTGCTCTGTTAGTAGCAGGTACTGCAA
>phoP_MicA     GTTTACCCCCATAACCACATAATCGCGTTACACTATTTTAATAATTAAGACAGGGAGAAATAAAAATGCGCGTACTGGTTGTTGAAGACAATGCGTTGTTACGTCACCACCTTAAAGTTCAGAT
>phoP_MicA     GTTTACCCCCATAACCACATAATCGCGTTACACTATTTTAATAATTAAGACAGGGAGAAATAAAAATGCGCGTACTGGTTGTTGAAGACAATGCGTTGTTACGTCACCACCTTAAAGTTCAGAT
>phoP_MicA     GTTTACCCCCATAACCACATAATCGCGTTACACTATTTTAATAATTAAGACAGGGAGAAATAAAAATGCGCGTACTGGTTGTTGAAGACAATGCGTTGTTACGTCACCACCTTAAAGTTCAGAT
>phoP_MicA     GTTTACCCCCATAACCACATAATCGCGTTACACTATTTTAATAATTAAGACAGGGAGAAATAAAAATGCGCGTACTGGTTGTTGAAGACAATGCGTTGTTACGTCACCACCTTAAAGTTCAGAT
>phoP_MicA     GTTTACCCCCATAACCACATAATCGCGTTACACTATTTTAATAATTAAGACAGGGAGAAATAAAAATGCGCGTACTGGTTGTTGAAGACAATGCGTTGTTACGTCACCACCTTAAAGTTCAGAT
>phoP_MicA     GTTTACCCCCATAACCACATAATCGCGTTACACTATTTTAATAATTAAGACAGGGAGAAATAAAAATGCGCGTACTGGTTGTTGAAGACAATGCGTTGTTACGTCACCACCTTAAAGTTCAGAT
>ptsG_SgrS     TGTACACGGCGAGGCTCTCCCCCCTTGCCACGCGTGAGAACGTAAAAAAAGCACCCATACTCAGGAGCACTCTCAATTATGTTTAAGAATGCATTTGCTAACCTGCAAAAGGTCGGTAAATCGCTGATGCTGC
>ptsG_SgrS     TGTACACGGCGAGGCTCTCCCCCCTTGCCACGCGTGAGAACGTAAAAAAAGCACCCATACTCAGGAGCACTCTCAATTATGTTTAAGAATGCATTTGCTAACCTGCAAAAGGTCGGTAAATCGCTGATGCTGC
>ptsG_SgrS     TGTACACGGCGAGGCTCTCCCCCCTTGCCACGCGTGAGAACGTAAAAAAAGCACCCATACTCAGGAGCACTCTCAATTATGTTTAAGAATGCATTTGCTAACCTGCAAAAGGTCGGTAAATCGCTGATGCTGC
>ptsG_SgrS     TGTACACGGCGAGGCTCTCCCCCCTTGCCACGCGTGAGAACGTAAAAAAAGCACCCATACTCAGGAGCACTCTCAATTATGTTTAAGAATGCATTTGCTAACCTGCAAAAGGTCGGTAAATCGCTGATGCTGC
>rpoS_DsrA     AAATAGCAACCATGGGTAGCACCGGAACCAGTTCAACACGCTTGCATTTTGAAATTCGTTACAAGGGGAAATCCGTAAACCCGCTGCGTTATTTGCCGCAGCGATAAATCGGCGGAACCAGGC
>sdhD_RyhB     TATCTGGAAGAAACATTCGAAGCGGGTAAACGCTCCGCCAAAATCTCCTTTGTTATTACTGTCGTGCTTTCACTTCTCGCAGGAGTCCTCGTATGGTAAGCAACGCCTCCGCATTAGGACGCAATGGCGTACATGATTTC
>serA_gcvB     CAAACGTTCATATTGCCGCAATATTATTTTTTGATATGTTGAAAGGCGGATGCAAATCCGCACACAACATTTCAAAGACAGGATTGGGTAAATGGCAAAGGTATCGCTGGAGAAAGACAAGATT
>shiA_RyhB     TCAGCCATAAAATCATTTGCAGATGCGCTCTGTCGAGGTAGTCTCATAAGGTTCGTTTATAGATCGACGCAATGTGAGTTACCTTTTCCATACTAATTATAAAAAGACAGTACAAACAGGATCATTATGGACTCCACGCTCATCTCCACT
>shiA_RyhB     TCAGCCATAAAATCATTTGCAGATGCGCTCTGTCGAGGTAGTCTCATAAGGTTCGTTTATAGATCGACGCAATGTGAGTTACCTTTTCCATACTAATTATAAAAAGACAGTACAAACAGGATCATTATGGACTCCACGCTCATCTCCACT
>shiA_RyhB     TCAGCCATAAAATCATTTGCAGATGCGCTCTGTCGAGGTAGTCTCATAAGGTTCGTTTATAGATCGACGCAATGTGAGTTACCTTTTCCATACTAATTATAAAAAGACAGTACAAACAGGATCATTATGGACTCCACGCTCATCTCCACT
>shiA_RyhB     TCAGCCATAAAATCATTTGCAGATGCGCTCTGTCGAGGTAGTCTCATAAGGTTCGTTTATAGATCGACGCAATGTGAGTTACCTTTTCCATACTAATTATAAAAAGACAGTACAAACAGGATCATTATGGACTCCACGCTCATCTCCACT
>shiA_RyhB     TCAGCCATAAAATCATTTGCAGATGCGCTCTGTCGAGGTAGTCTCATAAGGTTCGTTTATAGATCGACGCAATGTGAGTTACCTTTTCCATACTAATTATAAAAAGACAGTACAAACAGGATCATTATGGACTCCACGCTCATCTCCACT
>shiA_RyhB     TCAGCCATAAAATCATTTGCAGATGCGCTCTGTCGAGGTAGTCTCATAAGGTTCGTTTATAGATCGACGCAATGTGAGTTACCTTTTCCATACTAATTATAAAAAGACAGTACAAACAGGATCATTATGGACTCCACGCTCATCTCCACT
>sodB_FnrS     CACCTTTTAATTTGCTACCCTATCCATACGCACAATAAGGCTATTGTACGTATGCAAATTAATAATAAAGGAGAGTAGCAATGTCATTCGAATTACCTGCACTACCATATGCTAAAGATGCTCTGGCACCGCACA
>sodB_FnrS     CACCTTTTAATTTGCTACCCTATCCATACGCACAATAAGGCTATTGTACGTATGCAAATTAATAATAAAGGAGAGTAGCAATGTCATTCGAATTACCTGCACTACCATATGCTAAAGATGCTCTGGCACCGCACA
>sodB_FnrS     CACCTTTTAATTTGCTACCCTATCCATACGCACAATAAGGCTATTGTACGTATGCAAATTAATAATAAAGGAGAGTAGCAATGTCATTCGAATTACCTGCACTACCATATGCTAAAGATGCTCTGGCACCGCACA
>sodB_FnrS     CACCTTTTAATTTGCTACCCTATCCATACGCACAATAAGGCTATTGTACGTATGCAAATTAATAATAAAGGAGAGTAGCAATGTCATTCGAATTACCTGCACTACCATATGCTAAAGATGCTCTGGCACCGCACA
>sodB_FnrS     CACCTTTTAATTTGCTACCCTATCCATACGCACAATAAGGCTATTGTACGTATGCAAATTAATAATAAAGGAGAGTAGCAATGTCATTCGAATTACCTGCACTACCATATGCTAAAGATGCTCTGGCACCGCACA
>sodB_RyhB     GCTACCCTATCCATACGCACAATAAGGCTATTGTACGTATGCAAATTAATAATAAAGGAGAGTAGCAATGTCATTCGAATTACCTGCACTACCATATGCTAAAGATGCTCTGGCACCGCACATTTCT
>sodB_RyhB     GCTACCCTATCCATACGCACAATAAGGCTATTGTACGTATGCAAATTAATAATAAAGGAGAGTAGCAATGTCATTCGAATTACCTGCACTACCATATGCTAAAGATGCTCTGGCACCGCACATTTCT
>sodB_RyhB     GCTACCCTATCCATACGCACAATAAGGCTATTGTACGTATGCAAATTAATAATAAAGGAGAGTAGCAATGTCATTCGAATTACCTGCACTACCATATGCTAAAGATGCTCTGGCACCGCACATTTCT
>sodB_RyhB     GCTACCCTATCCATACGCACAATAAGGCTATTGTACGTATGCAAATTAATAATAAAGGAGAGTAGCAATGTCATTCGAATTACCTGCACTACCATATGCTAAAGATGCTCTGGCACCGCACATTTCT
>sodB_RyhB     GCTACCCTATCCATACGCACAATAAGGCTATTGTACGTATGCAAATTAATAATAAAGGAGAGTAGCAATGTCATTCGAATTACCTGCACTACCATATGCTAAAGATGCTCTGGCACCGCACATTTCT
>srlA_spf      CTTTCAATCTGATTAGATTAGGTTGCCGTTTGGTAATAAAACAATAAATCCTGAAGGAGAGAACAATGATAGAAACCATTACTCATGGTGCAGAGTGGTTTATCGGGCTGTTCCA
>srlA_spf      CTTTCAATCTGATTAGATTAGGTTGCCGTTTGGTAATAAAACAATAAATCCTGAAGGAGAGAACAATGATAGAAACCATTACTCATGGTGCAGAGTGGTTTATCGGGCTGTTCCA
>srlA_spf      CTTTCAATCTGATTAGATTAGGTTGCCGTTTGGTAATAAAACAATAAATCCTGAAGGAGAGAACAATGATAGAAACCATTACTCATGGTGCAGAGTGGTTTATCGGGCTGTTCCA
>srlA_spf      CTTTCAATCTGATTAGATTAGGTTGCCGTTTGGTAATAAAACAATAAATCCTGAAGGAGAGAACAATGATAGAAACCATTACTCATGGTGCAGAGTGGTTTATCGGGCTGTTCCA
>srlA_spf      CTTTCAATCTGATTAGATTAGGTTGCCGTTTGGTAATAAAACAATAAATCCTGAAGGAGAGAACAATGATAGAAACCATTACTCATGGTGCAGAGTGGTTTATCGGGCTGTTCCA
>tisB_IstR     GTTACCCGGCGCACTAAGTCCTGGCTGAAACGGGTGGTGCCGTCAGCGCCTTAACCCCGCGTGAGCACACTGTGTTATGTCAACGAGCACAACGTTTCTCCTTGAGATACCGCGTGCACAACAGCTGGCAACAGGCAGCGGAAA
>tppB_gcvB     AAAGCACCCCCGTTAATATGGGATGTAAAAAAAGAGGTAAAAGTGTCCACTGCAAACCAAAAACCAACTGAAAGCGTCAGTTTGAACGCTTTCAAACAACCGAAGGCGTTCTATCTCATC
>tppB_gcvB     AAAGCACCCCCGTTAATATGGGATGTAAAAAAAGAGGTAAAAGTGTCCACTGCAAACCAAAAACCAACTGAAAGCGTCAGTTTGAACGCTTTCAAACAACCGAAGGCGTTCTATCTCATC
>tppB_gcvB     AAAGCACCCCCGTTAATATGGGATGTAAAAAAAGAGGTAAAAGTGTCCACTGCAAACCAAAAACCAACTGAAAGCGTCAGTTTGAACGCTTTCAAACAACCGAAGGCGTTCTATCTCATC
>tppB_gcvB     AAAGCACCCCCGTTAATATGGGATGTAAAAAAAGAGGTAAAAGTGTCCACTGCAAACCAAAAACCAACTGAAAGCGTCAGTTTGAACGCTTTCAAACAACCGAAGGCGTTCTATCTCATC
>tppB_gcvB     AAAGCACCCCCGTTAATATGGGATGTAAAAAAAGAGGTAAAAGTGTCCACTGCAAACCAAAAACCAACTGAAAGCGTCAGTTTGAACGCTTTCAAACAACCGAAGGCGTTCTATCTCATC
>tppB_gcvB     AAAGCACCCCCGTTAATATGGGATGTAAAAAAAGAGGTAAAAGTGTCCACTGCAAACCAAAAACCAACTGAAAGCGTCAGTTTGAACGCTTTCAAACAACCGAAGGCGTTCTATCTCATC
>tppB_gcvB     AAAGCACCCCCGTTAATATGGGATGTAAAAAAAGAGGTAAAAGTGTCCACTGCAAACCAAAAACCAACTGAAAGCGTCAGTTTGAACGCTTTCAAACAACCGAAGGCGTTCTATCTCATC
>xylF_spf      TGATTGTTACTTATTAAAGCTGTCCTCTAACTACAGAAGGCCCTACACCATGAAAATAAAGAACATTCTACTCACCCTTTGCACCTCACTCCTGCTTACCAACGTTGCTGCACACGCCAAAGAAGTCAAAATAGGTATG
>xylF_spf      TGATTGTTACTTATTAAAGCTGTCCTCTAACTACAGAAGGCCCTACACCATGAAAATAAAGAACATTCTACTCACCCTTTGCACCTCACTCCTGCTTACCAACGTTGCTGCACACGCCAAAGAAGTCAAAATAGGTATG
>xylF_spf      TGATTGTTACTTATTAAAGCTGTCCTCTAACTACAGAAGGCCCTACACCATGAAAATAAAGAACATTCTACTCACCCTTTGCACCTCACTCCTGCTTACCAACGTTGCTGCACACGCCAAAGAAGTCAAAATAGGTATG
```

Figure S2 cont.

```
>xylF_spf    TGATTGTTACTTATTAAAGCTGTCCTCTAACTACAGAAGGCCCTACACCATGAAAATAAAGAACATTCTACTCACCCTTTGCACCTCACTCCTGCTTACCAACGTTGCTGCACACGCCAAAGAAGTCAAAATAGGTATG
>xylF_spf    TGATTGTTACTTATTAAAGCTGTCCTCTAACTACAGAAGGCCCTACACCATGAAAATAAAGAACATTCTACTCACCCTTTGCACCTCACTCCTGCTTACCAACGTTGCTGCACACGCCAAAGAAGTCAAAATAGGTATG
>yaeC_gcvB   TCAGTTCGCAGGCGACCGCATCGTCCGGGCTGTCACTCGCAAGTAACGTTCAACACAACATAAATAATTGAAGAAGGAATAAGGTATGGCGTTCAAATTCAAAACCTTTGCGGCAGTGGG
>yaeC_gcvB   TCAGTTCGCAGGCGACCGCATCGTCCGGGCTGTCACTCGCAAGTAACGTTCAACACAACATAAATAATTGAAGAAGGAATAAGGTATGGCGTTCAAATTCAAAACCTTTGCGGCAGTGGG
>yaeC_gcvB   TCAGTTCGCAGGCGACCGCATCGTCCGGGCTGTCACTCGCAAGTAACGTTCAACACAACATAAATAATTGAAGAAGGAATAAGGTATGGCGTTCAAATTCAAAACCTTTGCGGCAGTGGG
>yaeC_gcvB   TCAGTTCGCAGGCGACCGCATCGTCCGGGCTGTCACTCGCAAGTAACGTTCAACACAACATAAATAATTGAAGAAGGAATAAGGTATGGCGTTCAAATTCAAAACCTTTGCGGCAGTGGG
>yaeC_gcvB   TCAGTTCGCAGGCGACCGCATCGTCCGGGCTGTCACTCGCAAGTAACGTTCAACACAACATAAATAATTGAAGAAGGAATAAGGTATGGCGTTCAAATTCAAAACCTTTGCGGCAGTGGG
>yaeC_gcvB   TCAGTTCGCAGGCGACCGCATCGTCCGGGCTGTCACTCGCAAGTAACGTTCAACACAACATAAATAATTGAAGAAGGAATAAGGTATGGCGTTCAAATTCAAAACCTTTGCGGCAGTGGG
>yaeC_gcvB   TCAGTTCGCAGGCGACCGCATCGTCCGGGCTGTCACTCGCAAGTAACGTTCAACACAACATAAATAATTGAAGAAGGAATAAGGTATGGCGTTCAAATTCAAAACCTTTGCGGCAGTGGG
>yahO_micF   TGGCGTTTATGCCCTGACTGAACTAATTATTAATCAACCCAATAATGTCGGTGGGTGATAGTGTGATAACAACTCTGGAGCCGTAATATGAAAATAATCTCTAAAATGTTAG
>ybdH_gcvB   TGCTAGTATTTGGCAATCAAGACGTTTAGATGTCTAAATAAAACAATAAGGACAACACAACATGCCTCACAATCCTATCCGCGTGGTCGTCGGCCCGGCTAACTACTTTTCAC
>ybdH_gcvB   TGCTAGTATTTGGCAATCAAGACGTTTAGATGTCTAAATAAAACAATAAGGACAACACAACATGCCTCACAATCCTATCCGCGTGGTCGTCGGCCCGGCTAACTACTTTTCAC
>ybdH_gcvB   TGCTAGTATTTGGCAATCAAGACGTTTAGATGTCTAAATAAAACAATAAGGACAACACAACATGCCTCACAATCCTATCCGCGTGGTCGTCGGCCCGGCTAACTACTTTTCAC
>ybdH_gcvB   TGCTAGTATTTGGCAATCAAGACGTTTAGATGTCTAAATAAAACAATAAGGACAACACAACATGCCTCACAATCCTATCCGCGTGGTCGTCGGCCCGGCTAACTACTTTTCAC
>ybdH_gcvB   TGCTAGTATTTGGCAATCAAGACGTTTAGATGTCTAAATAAAACAATAAGGACAACACAACATGCCTCACAATCCTATCCGCGTGGTCGTCGGCCCGGCTAACTACTTTTCAC
```

Figure S2 related to Figure 2. ARN sequences are positioned adjacent or overlapping seed sequences. Forty-six experimentally verified mRNA seed sequences were scanned for the presence of ARN3, ARN4, and ARN5 with increasing numbers of mismatched positions within the motif (indicated by **m** in bold above each block of sequences). ARN motifs (blue) within 100nt of the seed sequence (bold) are indicated. The sRNA and mRNA interacting pair are indicated in the left hand column. See also Figure 2J for a cumulative plot of Hfq bound ARN5m2 motifs relative to mRNA seeds.

Figure S3

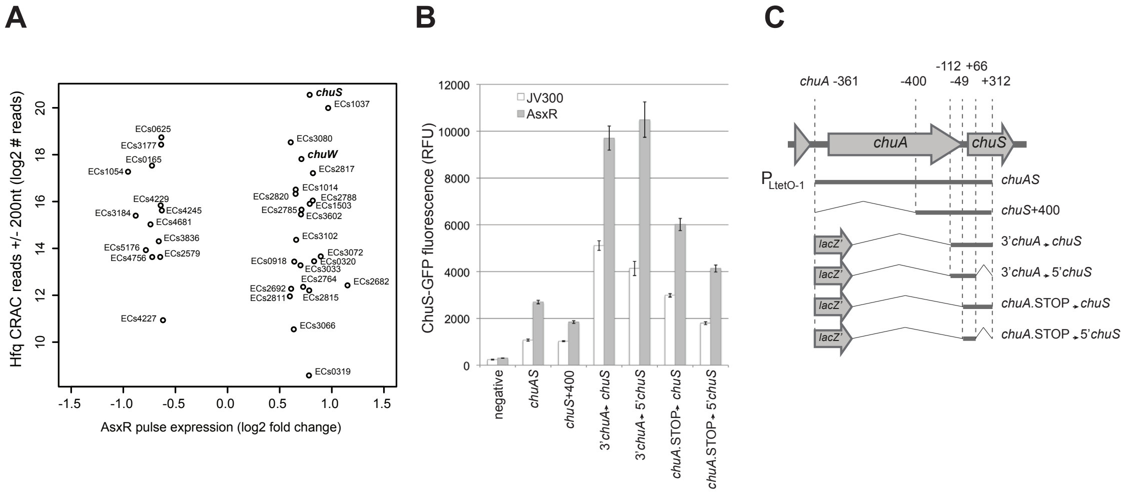

Figure S3, related to Figure 5. The Shiga toxin 2 locus encodes an anti-sRNA that stabilizes expression of the haem oxygenase, ChuS.

A. Microarray analysis of transcript stability after pulsed expression of AsxR (x-axis) was correlated with the amount of Hfq binding to features (within 200 base pairs; y-axis). The systematic names of features are given beside the data point and *chuS* and *chuW* are indicated with common names in bold. B&C. GFP translational fusions to the *chuS* coding sequence were constructed with varying lengths of upstream sequence (C). An upstream ribosomal binding site and short *lacZ’* fusion was included to allow translation coupling of *chuS* where indicated. (B) Fluorescence of the *chuS* translational fusions was measured with the control vector pJV300 (white) or vector expressing AsxR (grey). Error bars show SEM.

Figure S4

ARN5m2

AgvB	**CGATAACACAACAATATC**AGTATCTCATGCTATTGCCCGAACCCATTCGGGCATTTTT
AgvB	**CGATAACACAACAATATC**AGTATCTCATGCTATTGCCCGAACCCATTCGGGCATTTTT
AgvB	**CGATAACACAACAATATC**AGTATCTCATGCTATTGCCCGAACCCATTCGGGCATTTTT
AgvB	**CGATAACACAACAATATC**AGTATCTCATGCTATTGCCCGAACCCATTCGGGCATTTTT
AsxR	**CGATTATTAAAACG**AGTATCTCATGCAATTGCCCGAACCCACTCGGGCTTTTT
AsxR	**CGATTATTAAAACG**AGTATCTCATGCAATTGCCCGAACCCACTCGGGCTTTTT

ARN4m1

AgvB	**CGATAACACAACAATATC**AGTATCTCATGCTATTGCCCGAACCCATTCGGGCATTTTT
AgvB	**CGATAACACAACAATATC**AGTATCTCATGCTATTGCCCGAACCCATTCGGGCATTTTT
AsxR	**CGATTATTAAAACG**AGTATCTCATGCAATTGCCCGAACCCACTCGGGCTTTTT
Econc03	**AAAATGACAATGCA**AGTATCTCATGCTGTTGCCCGAACCCACTCGGGCTTTTT

Figure S4, related to Figures 4-7. ARNx motifs within anti-sRNA.

ARN5m2 and ARN4m1 motifs (blue) were identified in AgvB (EcOnco1), AsxR (EcOnc02), and EcOnc03. 5’ variable regions within respective anti-sRNA are indicated in bold.

SUPPLEMENTARY TABLES

Tables S1A-C, related to Figure 1 and Experimental Procedures.

Supplementary Table 1A: Top 100 CDS recovered from replicate *E. coli* O157 Hfq CRAC experiments.

	Systematic name	Common name	# reads									
			sample 1		sample 2		sample 3		sample 4		sample 5	
			sense	antisense	sense	antisense	sense	antisense	sense	antisense	sense	antisense
1	ECs3596	nlpD	571877	0	455412	0	507960	0	1771904	0	170920	0
2	ECs1881	pspA	901228	202	299771	729	2933767	372	927767	0	122599	0
3	tagA	tagA	189191	192424	285731	41656	243845	141554	681238	842774	74198	7363
4	cpxP	cpxP	141052	0	97373	0	120434	0	1483715	808	26021	0
5	ECs1526	ECs1526	0	211331	0	42714	29	203856	11	923140	0	11952
6	ECs4561	ECs4561	71288	0	172715	0	70619	0	150069	106	66806	0
7	ECs1812	ECs1812	177294	0	22537	0	81142	0	148750	508	9510	0
8	ECs0694	ybeJ	338341	0	27339	0	189793	0	129585	0	13814	0
9	ECs1488	ycfJ	173101	0	42433	0	140995	0	320684	0	18427	0
10	ECs3499	ECs3499	14	128136	265	21353	748	155072	725	153115	2	11988
11	ECs0535	ybaP	202	101976	0	4225	752	125447	28	202809	0	12643
12	ECs1741	adhE	74263	0	41047	629	108184	0	91450	217	14300	0
13	ECs0247	ykfE	12596	0	16655	0	98164	0	49575	0	12139	0
14	ECs1041	ompA	41162	1	50572	0	64170	398	92120	0	26636	0
15	ECs3782	ygfA	84051	0	13595	0	110904	1	158935	0	6489	0
16	ECs4555	ECs4555	56606	0	66463	0	66821	0	99517	0	20029	0
17	ECs1242	ECs1242	1456	26613	1114	7138	3342	73833	820	30859	0	8465
18	ECs3104	ompC	19035	0	16483	0	19133	0	60725	1	13856	0
19	ECs3595	rpoS	211189	0	21731	0	237939	0	64052	0	19226	0
20	ECs2263	ECs2263	108971	0	36772	0	48193	94	245270	3	8424	0
21	ECs1525	ECs1525	157054	0	44474	0	71551	113	310450	0	8763	0
22	ECs1180	ECs1180	38397	0	36237	0	138415	0	60988	83	5489	0
23	ECs3460	yfiA	85388	0	59061	0	86125	0	152724	0	19452	0
24	ECs1561	ECs1561	15013	0	42944	857	14645	0	73432	0	9514	0
25	espP	espP	24311	0	60891	3	32195	0	105550	1226	12485	0
26	ECs1814	ECs1814	15617	0	46633	3	84683	0	92477	103	11919	0
27	ECs1856	ECs1856	13286	0	66316	0	9978	1	102670	81	41260	0
28	ECs4551	ECs4551	14629	0	11328	0	15489	0	21293	0	6616	0
29	ECs3931	glgS	32229	0	5870	0	19238	0	65350	0	1	0
30	ECs2384	lpp	46428	0	15578	0	20797	0	50576	0	4021	4457
31	ECs5359	arcA	13353	0	10516	2	33468	0	124646	0	8544	0
32	ECs1037	rmf	28097	0	15788	0	51316	0	28601	0	5157	0
33	ECs4554	ECs4554	27025	0	16768	0	18610	0	39097	0	16208	0
34	ECs5432	ECs5432	17036	526	10451	1613	11312	469	70674	988	6182	0
35	ECs1883	pspC	22208	0	9801	0	25503	0	40450	0	6080	0
36	ECs1739	hns	16316	0	31827	1	25260	0	59993	100	744	0
37	ECs0160	yadR	10759	0	14764	0	25012	0	24615	0	6834	0
38	glnL	glnL	33020	0	8703	0	24864	0	81368	25	1088	0
39	ECs2844	ECs2844	9508	0	21061	0	15411	1036	49035	0	10990	0
40	ECs0149	dksA	19694	0	10315	0	10417	0	15224	0	3323	0
41	ECs1885	pspE	52561	0	11349	0	57168	0	68247	0	2916	0
42	ECs3270	b2390	5705	0	7221	0	13272	0	31384	451	6310	0
43	ECs4379	ECs4379	13779	2	21732	0	10433	0	35428	0	6005	0
44	ECs3328	b2466	2742	0	3188	0	5458	0	45453	0	6865	0
45	ECs1728	narK	80865	0	1548	738	14491	0	63550	393		
46	ECs3934	ECs3934	0	6596	0	17522	143	12252	151	22539	0	15542
47	ECs1663	ompT	11695	0	19008	0	15911	435	39323	216	1548	0
48	ECs2972	ECs2972	13176	0	11068	0	25424	0	16557	0	1870	1378
49	pspB	pspB	20976	0	6854	0	19747	0	33508	0	1940	0
50	ECs2845	ECs2845	8687	0	24446	0	14029	0	29168	36	3017	0
51	ECs4216	nirB	14032	0	28016	0	27604	0	46003	765		
52	pOSAK1_03	pOSAK1_03	7451	0	19540	0	11293	0	29084	1093	12190	0
53	tolB	tolB	6425	562	17506	0	11477	531	33136	547	170	0
54	ECs4571	ECs4571	30137	0	10154	0	16149	0	25560	0		
55	ECs2809	yeeX	10758	0	25492	0	17354	0	22578	0	1065	0
56	ECs1756	yciD	10960	0	3589	0	6717	0	11250	77	6328	0
57	ECs1067	ECs1067	8138	0	17985	0	9742	0	29491	0	9896	0
58	ECs2623	ECs2623	12532	0	7113	0	5990	0	17573	0	3499	0
59	ECs4556	ECs4556	16150	511	12508	0	5365	0	29321	257	163	0
60	ECs4572	ECs4572	10468	1	22475	0	6528	0	22870	0	5018	0
61	ECs1884	pspD	15464	0	5632	0	11128	0	18169	0	4389	0
62	ECs1954	ECs1954	7684	0	12537	0	10077	0	19545	108	5779	0
63	hlyB	hlyB	10745	1941	8636	471	15824	3100	20584	1852	126	1
64	uhpT	uhpT	12350	0	3648	0	9282	0	49408	0		
65	ECs2435	b1729	13704	0	3828	0	19786	394	2964	0		
66	ECs4557	ECs4557	12255	0	14665	800	6417	0	21258	0	3273	0
67	ECs2831	ECs2831	10683	0	10201	986	9583	341	25609	940	2788	0
68	ECs0494	hupB	16248	0	8244	0	11012	0	18551	0	1794	0
69	ECs1738	galU	8632	0	32998	0	9258	0	24668	339	8640	0
70	ECs4587	ECs4587	5917	0	31231	0	7946	0	20043	0	7049	0
71	glnA	glnA	12012	0	5859	0	9782	0	15223	0	1088	0
72	ECs4701	ilvL	12844	0	5023	0	13390	0	22990	0	899	0
73	ECs0752	sucB	4355	0	4599	0	11367	0	6639	0	2239	0
74	ECs1601	phoQ	43744	974	17747	0	127134	0	16322	818	1937	0
75	ECs3859	ECs3859	1825	0	246	0	5275	0	14116	0		
76	ECs2144	ydeH	3873	0	14666	0	3200	0	13941	0	5124	0
77	clpA	clpA	6928	0	11887	0	10769	0	17755	0	7641	0
78	ECs1669	minD	178	0		328	0	1255	1910	5244	0	
79	ECs3105	yojN	2211	0	5411	1	2948	0	11329	790		
80	ECs2390	ynhE	3364	0	2541	0	4113	0	8640	610	2	0
81	ECs0129	hpt				925	0	4798	0			
82	ECs1568	ECs1568	9229	1	11567	0	6034	0	19869	124	702	0
83	pO157p80	pO157p80	1750	2514	3326	822	8363	869	12974	7177	4725	3
84	ECs5135	frdA	13209	0	1532	0	9411	0	7712	0	4347	0
85	ECs0600	nfrA		1179	0	1	0	1354	0			
86	glnH	glnH	5334	0	6182	0	10172	0	8358	0		
87	hscB	hscB	1299	0	7964	0	9933	0	10267	0	4807	0
88	ECs4558	ECs4558	8857	0	1231	0	9411	0	10443	195	6497	0
89	ECs3745	ygeY	570	0								
90	ECs2839	ECs2839	5149	0	5894	0	3773	0	19604	0		
91	ECs4514	dfp	1088	0	25	0	804	0	1017	0	2	0
92	nirD	nirD	22055	0	574	0	10611	0	7589	0		
93	ECs0890	dps	9692	0	2512	0	4753	0	9035	0	1	0
94	ECs0624	fes	2767	0	3963	0	3443	0	11072	97		
95	ECs1204	ECs1204	17960	0	2067	0	7347	0	81965	102	2954	0
96	ECs3291	yfeK		1052	5	360	0		4417	0		
97	ECs3445	yfiD	21570	0	5856	0	9256	0	13934	0	884	0
98	ECs3846	ECs3846	559	0	402	298						
99	rrmJ	rrmJ	2310	0	1490	0	605	0	5680	0	441	0
100	ECs1914	ydaA	8172	0	4216	128	6515	0	17245	0	3181	0

Supplementary Table 1B: Top 100 intergenic regions recovered from replicate *E. coli* O157 Hfq CRAC experiments.

rank	Gene name	Flank CDS	# reads									
			sample 1		sample 2		sample 3		sample 4		sample 5	
			sense	Anti-sense	sense	Anti-sense	sense	Anti-sense	sense	Anti-sense	sense	Anti-sense
1	int_808	ECs0816..ECs0818	213049	446431	13508	56672	850386	587986	805446	828598	13198	44997
2	int_4750	polA..spf	0	502723	0	130181	0	729622	0	1942669	0	56570
3	int_1755	ECs1780..ECs1781	330301	11229	25949	3750	336462	10320	3093053	8782	8054	0
4	int_4805	ECs4839..yiiP	0	224231	0	179901	0	152250	0	2365028	0	33979
5	int_1921	ECs1960..ECs1961	223477	151477	10942	28069	541244	195114	2124832	168224	2009	14821
6	int_3437	ECs5503..ssrA	0	734955	0	26940	0	299803	0	247522	0	5974
7	int_4513	ECs4562..ECs4563	118226	1244	289706	4507	229682	6	152586	1284	48996	0
8	int_2699	ECs2746..ECs2748	249407	0	53745	0	120747	113	483834	0	23961	0
9	int_4873	ECs4902..tufB	0	204306	0	48901	0	204233	0	191282	0	10131
10	int_1185	ECs1206..ECs1207	113165	15377	16893	1360	74252	9334	1049436	11505	15779	5314
11	int_2215	ECs2255..ECs2257	159	638972	109	10181	202	207905	0	646467	1177	946
12	int_1337	ECs1362..ECs1364	0	218329	0	4071	0	57498	1277	458236	0	568
13	int_2152	ECs2189..ECs2190	112721	0	37959	0	61109	138	224177	88	12092	0
14	int_3732	ygfA..ECs5516	0	83385	0	13585	0	109919	0	156554	0	6489
15	int_1761	ECs1786..ECs1788	639881	179	9931	94	207645	251	645564	0	972	1121
16	int_1498	ECs1527..ECs1528	239630	351	36545	1	176412	477	529284	707	19881	0
17	int_4330	ECs4380..ECs4381	27661	0	8282	0	12418	2	134044	245	682	0
18	int_2223	ECs2263..ECs2264	134064	0	36400	0	56312	20	228459	9	6782	0
19	int_1927	ECs1966..ECs1967	639867	169	10013	107	207330	197	644695	0	971	1108
20	int_3501	ygaG..micA	378	16040	0	13484	835	25639	296	120350	0	2552
21	int_1071	ECs5404..ECs1088	26947	57784	6186	13413	41489	44519	90146	81379	5284	2104
22	int_690	sroC..ybeJ	332243	0	25813	0	183985	0	109209	0	9325	0
23	int_3216	yfeA..yfeC	50097	227	9489	0	39511	776	111756	1853	211	0
24	int_1082	ECs1100..ECs1101	956	56483	331	9115	349	56860	1537	160574	283	2438
25	int_1460	ycfJ..ycfQ	0	241206	0	30432	0	225193	0	427956	0	14640
26	int_3452	ECs3498..ECs3499	3143	222672	219	12103	9636	542125	5192	2129004	1110	1917
27	int_1209	ECs1231..ECs1232	81206	0	6030	0	70478	0	146246	0	3140	0
28	int_2837	yegQ..cyaR	0	62802	0	3736	0	145331	0	367789	0	684
29	int_5120	yjeR..yjeS	0	55174	0	8249	0	68016	26	65385	0	1381
30	int_4686	yifK..aslB	0	54115	0	22898	560	50713	494	84727	0	4966
31	int_1845	pspA..pspB	0	33353	0	3783	0	39154	0	58651	0	4824
32	int_4238	yhhX..ryhB	35599	525	7263	0	69481	529	106283	0	2649	0
33	int_5419	pO157p74..pO157p77	1166	15629	846	25175	5677	20367	1984	78377	0	8808
34	int_2912	ECs2972..ECs2973	19025	20062	2911	5516	20369	22410	16248	52927	68	369
35	int_533	micM..ybaP	0	100754	0	1969	0	123969	0	196161	0	12643
36	int_2610	ECs2646..pgsA	43180	0	11670	1441	47117	35	38667	1495	3798	0
37	int_5007	yjbO..qor	0	44120	0	4972	0	26740	0	77782	0	2488
38	int_2514	ryeA..ryeB	20794	10729	2516	2372	19071	10597	40568	29175	6	6
39	int_1182	ECs1203..ECs1204	0	66656	0	7949	0	18601	715	163078	0	13773
40	int_2675	ECs2718..ECs2721	245	20847	242	0	87	21547	133	32690	93	0
41	int_4857	oxyS..oxyR	3815	840	5126	0	10859	297	57012	0	11	1
42	int_2914	ECs2974..ECs2975	28890	0	5826	0	15211	0	100319	220	3479	0
43	int_1699	narK..narG	0	163056	0	3987	244	24618	0	104675		
44	int_220	dnaQ..ECs0212	0	26328	0	4973	0	13289	173	35598	0	3421
45	int_5415	pO157p68..pO157p69	6825	19267	7210	3346	15529	12283	11045	14738	3575	896
46	int_209	rrnH..rrnH	0	19558	6	4832	0	23512	0	24953	0	570
47	int_746	sucD..ECs0755	59	29189	0	1412	0	29922	0	34124	0	929
48	int_2123	ECs2161..ECs2162	241	21338	235	0	110	21591	100	32310	124	0
49	int_2884	ECs2942..ECs2945	235	21313	231	438	195	21528	390	33250	126	0
50	int_1229	ECs1251..ycdG	27994	548	13004	213	25525	0	35095	426	1623	0
51	int_4346	yhiW..gadY	0	29204	0	904	0	33839	0	43590	0	483
52	int_3505	yqaB..csrA	89037	350	4806	0	59289	187	51158	0	8796	0
53	int_532	ybaK..micM	0	26405	0	237	0	27823	479	24629	0	1241
54	int_3687	ECs3737..ECs3738	25867	0	566	0	25971	0	16491	93	65	0
55	int_4168	yhfC..nirB	0	19421	0	11868	0	23470	0	14078		
56	int_3212	ECs3269..ECs3270	0	1882	0	7738	0	12074	0	11997	0	2037
57	int_4968	ECs4993..ECs4994	15921	0	5032	0	11303	1	91585	187	2303	0
58	int_1495	ECs1524..ECs1526	0	21025	0	7431	0	21458	0	80663	0	4623
59	int_2550	yecK..cutC	9886	0	273	0	13005	0	41404	0	1	0
60	int_2012	ECs2048..ECs2049	15223	0	4942	0	12226	0	40069	635	4110	0
61	int_5310	ECs5328..yjjT	6075	227	1859	2656	6288	279	20523	2781		
62	int_3221	gltX..ECs3279	0	16035	974	4126	243	13332	0	16368	0	4
63	int_742	b0725..sucA	0	888	0	2069	0	256	0	4219	0	3
64	int_1895	recE..racC	0	2017	0	247	0	1374	0	5561		
65	int_1214	ECs1236..ECs1237	6697	5519	24	227	10190	5289	9056	5515	843	0
66	int_3414	yfiA..pheA	0	16357	0	4515	0	8103	0	22874	0	35
67	int_4739	rrnA..rrnA	0	19599	3	4828	0	23424	0	24800	0	543
68	int_1754	ECs1779..ECs1780	6193	0	27581	0	8083	0				
69	int_4093	rrnD..rrnD	17418	0	4687	8	18797	0	24099	0	110	0
70	int_4554	yicP..uhpT	6948	0	747	0	3806	1	33838	0		
71	int_1706	rttR_1..purU	29413	0	6247	0	20254	0	13132	0	138	0
72	int_4653	rrnC..yifA	0	12327	0	3190	0	29486	0	29183	0	719
73	int_4738	hemG..rrnA	0	11446	0	1243	0	15735	124	8024	0	703
74	int_3228	zipA..cysZ	977	0	1549	1716	6032	0	5048	101		
75	int_103	lpxC..yacA	0	5587	0	719	0	6570	0	22829	0	4140
76	int_2726	ECs2774..ECs2775	863	10915	144	61062	0	4922	1436	21375	0	36770
77	int_4866	murI..rrnB	0	11297	0	1363	0	14150	116	4833	0	723
78	int_4650	yieP..rrnC	0	11567	0	1458	0	14790	104	5402	0	4
79	int_198	yaeQ..yaeJ	0	2724	0	13104	0	7167	0	5056	0	3930
80	int_4091	rrnD..rrnD	16931	0	1157	0	9512	0	24232	0	1126	0
81	int_211	rrnH..yafB	413	27402	0	4999	0	13914	0	37324	0	4280
82	int_4170	nirD..nirC	0	21924	0	4	0	10912	0	4858		
83	int_4902	purH..rrnE	151	11283	0	1443	0	13908	93	4376	0	740
84	int_5125	mutL..miaA	0	885	0	1682	0	599	796	3660		
85	int_2734	nac..erfK	259	10849	0	61177	233	4908	107	21646	853	36768
86	int_3623	mltA.ECs3674	317	10250	0	8859	1	11543	0	23271	2	3309
87	int_5422	ECp088.2c..pO157p79	0	10167	0	11191	0	11864	1	22557	1	4215
88	int_2732	b1983..cbl	13842	10999	62232	61414	6746	5015	21998	21394	37522	36837
89	int_2980	mglB..galS	5859	0	5667	0	10089	0	37853	0	5	0
90	int_3643	aas..omrA	2157	0	7693	0	10556	0	47221	185	2	0
91	int_1818	yciH..ECs1856	4982	0	52736	0	4711	0	84784	0	24688	0
92	int_4094	rrnD..yrdA	9443	0	671	0	12807	0	2750	100	675	0
93	int_3644	omrA..omrB	4437	0	2096	0	8452	0	45085	0	556	0
94	int_1885	b1342..fnrS	0	12546	0	4146	0	14320	135	19515		
95	int_894	moeA..ECs0908	0	919	0	2061	0	1474	0	2041		
96	int_1072	ECs1088..ECs1089	4668	15	12	3	10866	167	17808	341		
97	int_2388	infC..thrS	825	0	283	0	997	0	5	0		
98	int_3019	yejM..yejO	0	9177	0	1878	0	11128	0	12202		
99	int_2787	ECs5479..ECs2840	3257	0	1158	0	1631	0	8830	0		
100	int_3618	ECs5511..ECs3669	0	26929	1125	618	0	5903	228	122808	0	4037

Supplementary Table 1C: Top sRNAs recovered from all five replicate *E. coli* O157 Hfq CRAC experiments.

rank	sRNA	# reads									
		sample 1		sample 2		sample 3		sample 4		sample 5	
		sense	antisense	sense	antisense	sense	antisense	sense	antisense	sense	antisense
1	spf	1765865	0	320067	0	1083232	0	6020137	0	160617	0
2	micM	594192	0	78189	0	587681	0	815665	0	65971	0
3	omrB	291335	0	173366	0	487442	0	2291431	0	22001	0
4	sroC	537693	0	97833	0	319057	0	672565	0	22337	0
5	ryhB	811573	0	49458	0	399231	0	1144734	0	9962	0
6	omrA	304748	0	170158	0	418754	0	825662	0	20435	0
7	cyaR	297053	0	4870	0	208543	0	445204	74	869	1
8	micA	80327	0	27819	0	88076	0	941407	313	3080	0
9	rprA	123318	0	52354	2	88770	0	1360487	0	11043	0
10	ssrA	734955	0	26940	0	299803	0	247522	0	5974	0
11	ryeA	19454	73261	2421	9012	30001	83888	42028	76084	12	2995
12	rybB	81523	0	8881	0	146377	0	164066	418	15908	0
13	micF	52518	0	19278	0	58265	0	192313	21	2105	0
14	fnrS	126374	0	17970	0	76698	0	274570	68	4728	0
15	glmZ	42113	0	8976	0	12365	0	105472	0	5023	0
16	arcZ	42631	1061	6576	0	54155	241	94767	2425	7050	1
17	oxyS	9755	0	7569	0	16029	0	121347	0	495	0
18	ryeB	20794	10572	2516	2372	19071	9830	40568	28078	6	6
19	isrA	286599	0	14089	0	161765	0	364144	175	1255	0
20	yejG_bcr	2250	0	6643	1	21393	0	19237	365		
21	mgrR	19830	0	1366	0	13190	0	40856	0	765	0
22	sroH	9112	0	2644	0	2509	395	12388	216	3	0
23	sibE	4585	0	6266	0	10329	0	7649	67		
24	rnpB	50206	0	1407	0	9523	0	63354	0	3269	0
25	ryfD	4882	0	3151	0	3877	0	9731	0	3298	0
26	sibB	5567	0	1302	0	7142	0	5494	0	1	0
27	rydC	1099	0	824	0	1437	0	1966	66	2	0

Table S2, related to Figure 4.

Supplementary Table 2: Orphan *E. coli* O157 Hfq binding sites predicted to encode small RNA.

Chromosome	Rho Term*	Peak start	Peak end	Num. pass†	strand	AUC‡	Name	Sp**	Northern	Homologues††	Δ*hfq*‡‡
chr	✓	1941783	1941835	5	-	77806407	EcOnc01a	Sp10 (AgvB1)	60nt	EcOnc01b (AgvB2)	Destabilised
chr	✓	1268494	1268542	5	-	40531943	EcOnc02	Sp5 (AsxR)	54nt		Destabilised
chr	✓	1774497	1774550	5	-	23594091	EcOnc03	Sp9	56/~54/51nt		Destabilised
chr	✓	2188592	2188649	5	-	5701677	EcOnc04	Sp11	~222nt	Ecs1525/Ecs2262/ Ecs2746/Ecs2189	
chr	✓	1179806	1179914	5	+	5595206	EcOnc05	Sp4	~222nt		
chr	✓	3486349	3486411	5	-	4773645	EcOnc06	Sp17	~177nt	5' ECs1961	
chr	✓	3306848	3307010	5	-	3293889	EcOnc07	core	~256nt		Stable
chr	✓	1179938	1180010	5	-	3161901	EcOnc08	Sp4	~271nt	EcOnc21/EcOnc42	
plasmid_pO157	✓	77357	77404	5	+	901905	EcOnc09	pO157			
chr	✓	2234239	2234287	4	+	33571791	EcOnc10	Sp12			
chr	✓	1946819	1946867	4	-	33244073	EcOnc11	Sp10			
chr	✓	1779522	1779569	4	-	33130917	EcOnc12	Sp9			
chr	✓	901332	901382	4	+	26841197	EcOnc13	Sp3	~187nt		
chr	✓	1422407	1422451	4	+	23250801	EcOnc14	SpLE1		Esr41***	
chr	✓	2925956	2926022	4	-	4245599	EcOnc15	Sp15 (stx1)			
plasmid_pOSAK1	✓	2534	2586	4	-	3973450	EcOnc16	pOSAK1			
plasmid_pO157	✓	604	651	4	-	2073595	EcOnc17	pO157			
plasmid_pO157	✓	73870	73948	4	+	702533	EcOnc18	pO157	~37nt		
chr	✓	4517084	4517138	4	-	499583	EcOnc19	pO157			
chr	✓	1255628	1255753	4	-	485948	EcOnc20	Sp5 (stx2)			
chr	✓	1180016	1180101	3	-	2708682	EcOnc21	Sp4	~271nt	EcOnc08/EcOnc42	
chr	✓	2902714	2902774	3	+	2220614	EcOnc22	Sp15 (stx1)	~85nt	EcOnc23/24	Destabilised
chr	✓	2676643	2676704	3	+	2208426	EcOnc23	Sp14		EcOnc22/24	
chr	✓	2166694	2166753	3	+	2199017	EcOnc24	Sp11		EcOnc22/23	
chr	✓	961892	961982	3	-	793148	EcOnc25	core			
plasmid_pO157	✓	66559	66614	3	-	722445	EcOnc26	pO157			
chr	✓	1268411	1268490	3	+	503860	EcOnc27	Sp5 (stx2)	~235/247nt		247nt destabilised
chr	✓	1774391	1774436	3	+	466209	EcOnc28	Sp9			
chr	✓	4400137	4400196	3	-	381056	EcOnc29	core			
plasmid_pO157	✓	86331	86411	3	+	262855	EcOnc30	pO157			
chr	✓	1308663	1308739	3	-	209649	EcOnc31	Sp5 (stx2)			
chr	×	901574	901662	5	-	64660579	EcOnc32	Sp3	~112nt		
chr	×	1557089	1557136	5	-	25562373	EcOnc33	Sp6	~71nt		
plasmid_pO157	×	956	1004	5	-	24820901	EcOnc34	pO157			
chr	×	1186583	1186656	5	+	8159142	EcOnc35	Sp4			
chr	×	1301433	1301515	5	-	3859351	EcOnc36	Sp5			
chr	×	5077340	5077410	5	-	3370971	EcOnc37	Sp18			
chr	×	2032853	2032902	5	-	1638579	EcOnc38	core	~256nt		~4nt shorter
plasmid_pO157	×	73864	73908	5	-	610603	EcOnc39	pO157			
chr	×	1397649	1397699	4	+	3534015	EcOnc40	SpLE1			
chr	×	1418246	1418295	4	+	2415419	EcOnc41	SpLE1			
chr	×	2924036	2924118	4	+	2235798	EcOnc42	Sp15 (stx1)	~271nt	EcOnc21/EcOnc08	
chr	×	1308356	1308404	4	-	823541	EcOnc43	Sp5 (stx2)			
chr	×	1397056	1397117	4	-	513135	EcOnc44	SpLE1			
plasmid_pO157	×	78999	79065	4	+	466779	EcOnc45	pO157			
chr	×	2896476	2896522	4	-	342840	EcOnc47	Sp15 (stx1)			
chr	×	3104106	3104149	4	+	243215	EcOnc48	core			
chr	×	1772764	1772808	3	-	1228800	EcOnc49	Sp9			
chr	×	1266899	1266967	3	-	1098268	EcOnc50	Sp5 (stx2)	~78nt		Destabilised
chr	×	2237479	2237555	3	+	1091434	EcOnc51	Sp12			
chr	×	1181998	1182075	3	-	1027603	EcOnc52	Sp4			
chr	×	1292386	1292438	3	-	784285	EcOnc53	Sp5 (stx2)	Not detected		
chr	×	1577126	1577187	3	-	655723	EcOnc54	Sp6			
chr	×	690728	690809	3	-	588272	EcOnc55	S-loop45			
plasmid_pO157	×	2902	2949	3	-	517176	EcOnc56	pO157			
plasmid_pO157	×	70463	70518	3	-	498236	EcOnc57	pO157			
plasmid_pO157	×	86141	86249	3	+	433086	EcOnc58	pO157			
chr	×	2340964	2341010	3	+	340212	EcOnc59	core			
plasmid_pO157	×	73796	73848	3	+	310802	EcOnc60	pO157			
chr	×	3863130	3863181	3	+	223329	EcOnc61	SpLE3			
chr	×	1735063	1735106	3	-	193501	EcOnc62	core			
chr	×	4178965	4179013	3	+	163297	EcOnc63	core			
chr	×	1381282	1381341	3	+	151016	EcOnc64	SpLE1			

*Presence of a Rho-independent terminator within 200 nt of the 3' edge of the peak. Rho-independent termonators were predicted using RNAmotif (Mackie *et al* 2001)and the Rho-independent terminator descriptor described by Lesnik *et al* 2001.

†The number of Hfq CRAC datasets that an overlapping peak was identified.

‡Area under the curve (peak). Cumulative score of reads per base +/- 20nt from the peak maxma. NB: Not a measure of reads. Used to rank peaks.

**Sp-loop. Where the predicted sRNA falls within a pathogenicity island (S-loop) or pathogenicity island encoding a prophage (Sp-loop), the pathogenicity island designation is given. Coordinates to pathogenicity islands are available at http://genome.bio.titech.ac.jp/cgi-bin/o157/loop.pl?table=loop

††Repeats of predicted sRNAs encoded within the *E. coli* O157:H7 str. Sakai genome are given. Where homologues or repeats are not predicted and assigned an EcOnc number, the position (5' or 3') is indicated relative to the nearest ORF (ECs). Where homologues or repeats fall within ORFs, these are given.

‡‡Stability of predicted sRNA in *E. coli* O157:H7 str. Sakai Δ*hfq*.

***Published while in review. Sudo, N., Soma, A., Muto, A., Iyoda, S., Suh, M., Kurihara, N., Abe, H., Tobe, T., Ogura, Y., Hayashi, T., Kurokawa, K., Ohnishi, M., and Sekine, Y. (2014). A novel small regulatory RNA enhances cell motility in enterohemorrhagic *Escherichia coli*. J Gen Appl Microbiol *60*, 44-50.

Tables S3A-C, related to Experimental Procedures.

Supplementary Table 3A: Strains used in this study

Serotype	Strain	Genotype	Reference
E. coli O157:H7	Sakai	Δ*stx1 stx2A::kan*, Kan^R	Dahan et al., 2004
	Sakai Δ*hfq*	Δ*hfq*	This study
	Sakai *hfq*::HTF	*hfq*::HTF	This study
	Sakai Δ*agvB2* Δ*agvB1*	Δ*agvB2* Δ*agvB1::tetRA*, Tet^R	This study
	Sakai Δ*agvB2* Δ*agvB1::agvB1*	Δ*agvB2* Δ*agvB1::agvB1,* genomic deletion *agvB* repaired by marker rescue	This study
	TUV93-0	ΔBP-933W (*stx2*Φ) ΔCP-933V (*stx1*Φ)	Campellone et al., 2004
E. coli K12	MG1655		Blattner et al., 1997
	MG1655 *hfq*::HTF	MG1655 *hfq*::HTF	This study
	Top10F'	F´{*lac*Iq, Tn*10*(TetR)} *mcr*A Δ(*mrr-hsd*RMS-*mcr*BC) Φ80*lac*ZΔM15 Δ*lac*X74 *rec*A1 *ara*D139 Δ(*ara leu*) 7697 *gal*U *gal*K *rps*L (StrR) *end*A1 *nup*G	Invitrogen
	DH5α	*fhuA2 lac(del)U169 phoA glnV44* Φ80' *lacZ(del)M15 gyrA96 recA1 relA1 endA1 thi-1 hsdR17*	Taylor et al., 1993

Supplementary Table 3B: Plasmids used in this study

Plasmid	Description	Reference
pTOF25	Temperature sensitive allelic exchange vector. Cm^R	Merlin et al., 2002
pTOF24	Temperature sensitive allelic exchange vector. Cm^R	Merlin et al., 2002
pCP20	Temperature sensitive construct for expression of FLIP recombinase. Cm^R	Cherepanov and Wackernagel, 1995
pTOF25::*hfq*-HTF::*tetRA*	Allelic exchange vector used to insert HTF tag. Cm^R Tet^R	This study
pTOF24::*agvB1<>tetRA*	Allelic exchange vector used to delete *agvB1*	This study
pTOF24::*agvB2<>tetRA*	Allelic exchange vector used to delete *agvB2*	This study
pMI	MicF$_{sal}$ expression construct. Amp^R	Corcoran et al., 2012
pOmpF	Constitutively transcribed *ompF*$_{sal}$-GFP translational fusion. Cm^R	Corcoran et al., 2012
pJL18-1	Constitutively transcribed *dppA*$_{sal}$-GFP fusion. Cm^R.	Sharma et al., 2007
pXG10::DppA.G1	G1 mutant of pJL18-1	This study
pXG10SF::DppA$_{EHEC}$	Constitutively transcribed *dppA*$_{EHEC}$-sfGFP fusion. Cm^R.	This study
pZA21MCS	P$_{LtetO-1}$ expression vector. p15A replicon, Kan^R.	Expressys, Germany
pZE12*luc*	P$_{LlacO-1}$ luciferase expression vector. ColE1 replicon, Amp^R	Expressys, Germany
pJV300	Scrambled RNA control for pZE12 expressed sRNAs	Sittka et al., 2007
pXG0	P$_{LtetO-1}$ luciferase expression vector. SC101 replicon, Cm^R	Urban and Vogel, 2007
pXG10SF	P$_{LtetO-1}$ superfolderGFP fusion vector. SC101 replicon, Cm^R	Corcoran et al., 2012
pXG30SF	P$_{LtetO-1}$ superfolderGFP fusion vector. SC101 replicon, Cm^R. Contains upstream FLAG-lacZ ORF for translational coupling.	Corcoran et al., 2012
pXG10SF::*chuAS*	-361 *chuA*→ +312 *chuS*	This study
pXG10SF::*chuS*+400	-400→ +312 *chuS*	This study
pXG30SF::3'*chuA*→*chuS*	-112→+312 *chuS*	This study
pXG30SF::3'*chuA*→5'*chuS*	-112→+66 *chuS*	This study
pXG30SF::*chuA*.STOP→*chuS*	-49→+312 *chuS*	This study
pXG30SF::*chuA*.STOP→5'*chuS*	-49→+66 *chuS*	This study
pXG30SF::3'*chuA*→5'*chuS*.F1	F1 mutant of pXG30SF::3'*chuA*→5'*chuS*	This study
pZA21::RyhB	pZA21 with RyhB cloned at the transcriptional +1 site	This study
pZA21::FnrS	pZA21 with FnrS cloned at the transcriptional +1 site	This study
pZA21::FnrS.S1	S1 mutant of pZA21::FnrS	This study
pZA21::FnrS.F1	F1 mutant of pZA21::FnrS	This study
pZA21::GcvB	pZA21 with GcvB cloned at the transcriptional +1 site	This study
pZA21::GcvB.G1	G1 mutant of pZA21::GcvB	This study
pZA21::RyeB	pZA21 with RyeB cloned at the transcriptional +1	This study

	site	
pZE12::AgvB (pAgvB)	pZE12 with AgvB (EcOnc01) cloned at +1	This study
pZE12::AsxR	pZE12 with AsxR (EcOnc02) cloned at +1 site	This study
pZE12::AsxR.S1	S1 mutant of pZE12::AsxR	This study
pZE12::EcOnc03	pZE12 with EcOnc03 cloned at +1	This study
pBAD+1	pBAD*myc*His A with MCS removed from +1 to terminator.	This study
pBAD+1::AsxR	AsxR cloned at the +1 site of pBAD+1	This study

Supplementary Table 3C: Oligonucleotides used in this study

**Primers**	**Sequence**	**Purpose**
hfq.5.NotI.F	aaaaaGCGGCCGCCGAAGCGGCAGATAACCTGG	Generating 5' SOE PCR product
hfq.5.BamHI_link.R	CCGTTCCAAGGATCCAAGAGCGTTCGGTTTCTTCGCTGTCCTG	Generating 5' SOE PCR product
hfq.3.BamHI_link.F	CGCTCTTGGATCCTTGGAACGGGGTTTCGGGCTGTTTTTTTTACACGGGGAGC	Generating 3' SOE PCR product
hfq.3.NotI.R	aaaaaGCGGCCGCTCTTTCAAGGTGGGTCCAGC	Generating 3' SOE PCR product
agvB1.PstI.F	tgggggCTGCAG AGCAGACGGCCAGCAGAACG	Generating 5' SOE PCR product
agvB.NotI.5.R	ccgttccaagcggccgcaagagcgTGTGGATTTTAACCAGGGTT	Generating 5' SOE PCR product
agvB1.NotI.3.F	cgctcttgcggccgcttggaacggACCCATAAAAAAGCCCCTCCGGAGAG	Generating 3' SOE PCR product
agvB1.SalI.R	tgggggGTCGAC CGGCGCATATCAACCACAGAGC	Generating 3' SOE PCR product
agvB2.PstI.F	tgggggCTGCAG AGCACACCGCCAGCAACACA	Generating 5' SOE PCR product. Use with agvB.NotI.5.R
agvB2.NotI.3.F	cgctcttgcggccgcttggaacggACCCATAAAAAATGCCCCTCCGGAGAG	Generating 3' SOE PCR product
agvB2.SalI.R	tgggggGTCGAC GCTGTGGAATTATTGTTTGTGC	Generating 3' SOE PCR product
agvB1.ext.R	GCAATCGGTCAGTGGTTCGAC	Confirming insertion
agvB2.ext.R	TATCGTCATTGCTATATTTC	Confirming insertion
agvB1-comp-for	GTCTCGGTACCCGACCTGCAGAGCAGACGGCCAGCAGAACG	Marker rescue of Δ*agvB1*::*tetRA*
agvB1-comp-rev	TCCCATTCGCCACCGGTCGACCGGCGCATATCAACCACAGAGC	Marker rescue of Δ*agvB1*::*tetRA*
chuA.+361.NsiI.F	gttttATGCATTGTTTATTGTGTTAATGGTGG	Cloning ChuAS fusion
chuS+400.NsiI.F	gttttATGCATGTGGCCTATAACCGTACCC	Cloning ChuAS fusion
chuA.3'.NsiI.F	gttttATGCATGTCGCCGCAAGGCATCCCAC	Cloning ChuAS fusion
chuA.STOP.NsiI.F	gttttATGCATTAATCATCTGCCCCGATATTTCG	Cloning ChuAS fusion
chuS.5'.NheI.R	gttttGCTAGCGATGTCACGCGCGTACTTTCC	Cloning ChuAS fusion
chuS.NheI.R	gttttGCTAGCATTGAGAAACAGACGTAAATC	Cloning ChuAS fusion
chuS.F1.F	CTGTATGAACCACTACACACGCTGGCTTGAG	Generating F1 mutant of ChuAS fusion
chuS.F1.5P.R	5P-TCTCTTCCTTCCAGATAAATGC	Generating F1 mutant of ChuAS fusion
DppA.sal.G1.R	TTGCCCTTTGCAAAGGCTTTTACCG	Generating G1 mutant of $DppA_{sal}$ fusion (pJL18-1)
DppA.sal.G1.5P.F	5P-TTGTACATACATCACAATTGGAGCAGAAGAATGA	Generating G1 mutant of $DppA_{sal}$ fusion (pJL18-1)
DppA.EHEC.NsiI.F	gttttATGCAT tcacgaggggcattttatgg	Generating $DppA_{EHEC}$ XG10SF fusion
DppA.EHEC.NheI.R	gtttt GCTAGC CATCCCTGACTTTTTCAAGG	Generating $DppA_{EHEC}$ XG10SF fusion
ZE12.5P.R	5P-GTGCTCAGTATCTTGTTATCC	To clone anti-sRNA by PCR
EcOnc01.ZE12.F	CGATAACACAACAATATCAGTATCTCATGCTATTGCCCGAACCCATTCGGGCATTTTTGGCATCAAATAAAACGAAAG	To clone EcOnc01 by PCR
EcOnc01.G1.F	CGATAACACTTGTATATCAGTATCTCATGCTATTG	Generating G1 mutant of EcOnc01
AsxR.ZE12.F	CGATTATTAAAACGAGTATCTCATGCAATTGCCCGAACCCACTCGGGCTTTTTGGCATCAAATAAAACGAAAG	To clone AsxR by PCR
AsxR.S1.F	CGATTAAGTAAACGAGTATCTCATGCAATTGCCCG	Generating S1 mutant of AsxR
ZA21MCS.5P.R	5P-GTGCTCAGTATCTCTATCACTGA	Amplifying ZA21 to clone into +1
ZA21MCS.HindIII.F	gtttt AAGCTT GGTACGCGTGCTAGAGGCATC	Amplifying ZA21 to clone into +1
GcvB.5P.F	5P-ACTTCCTGAG CCGGAACGAA	Amplify GcvB for ZA21
GcvB.HindIII.R	gaaaaaAAGCTTAAAAAAAGCACCGCAATTAGGCGGTGC	Amplify GcvB for ZA21
GcvB.G1.5P.F	5P-TGTACAAGTGTTTGCAATTGG	Generate G1 mutant of GcvB
GcvB.G1.R	CAACATCACAACCGTAAGCCA	Generate G1 mutant of GcvB
FnrS.5P.F	5P-GCAGGTGAATGCAACGTCAAG	Amplify FnrS for ZA21

FnrS.HindIII.R	gaaaaaAAGCTTAAAAAGCCGACTCATCAAAGTCGGCG	Amplify FnrS for ZA21
FnrS.S1.F	ACTTGAGTCGGCTTTTTAAGCTTGGTACGCGTG	Generate S1 mutant of FnrS
FnrS.S1.5P.R	5P-AAGTCGGCGTCGTACGAATCAATTGTGC	Generate S1 mutant of FnrS
FnrS.F1.F	ACAGTTACTTCCTTTTTTGAATTACTGCATAGCAC	Generate F1 mutant of FnrS
FnrS.F1.5P.R	ATATGGAGCGCAACGCCCATCGCTTG	Generate F1 mutant of FnrS
RyhB.5P.F	5P-GCGATCAGGAAGACCCTCGC	Amplify RyhB for ZA21
RyhB.HindIII.R	gaaaaaAAGCTTAAAAAGCCAGCACCCGGCTGGC	Amplify RyhB for ZA21
BAD+1.5P.R	5P-TAT GGA GAA ACA GTA GTA GAG AG	Amplify pBAD for sRNA cloning
BAD+1.XbaI.F	aaaaa TCTAGA TTTGCCTGGCGGCAGTAGCG	Amplify pBAD for sRNA cloning
BAD+1.AsxR.F	CGATTATTAAAACGAGTATCTCATGCAATTGCCCGAACCCA CTCGGGCTTTTTTTTGCCTGGCGGCAGTAGCG	Amplify pBAD+1 with AsxR
P5	AATGATACGGCGACCACCGAGATCTACACTCTTTCCCTACA CGACGCTCTTCCGATCT	Forward primer to amplify RACE products and CRAC cDNA libraries
PE_miRCat	CAAGCAGAAGACGGCATACGAGATCGGTCTCGGCATTCCT GGCCTTGGCACCCGAGAATTCC	To amplify CRAC cDNA libraries
L5 linker	5'invddT-ACACrGrArCrGrCrUrCrUrUrCrCrGrArUrCrU-barcode	5' linker for RLM-RACE and CRAC. barcode = 2-11nt of unique sequence
EcOnc01.5.RACE.R	CATGAGATACTGATATTGTT	EcOnc01 5'RLM-RACE
EcOnc02.5.RACE.R	TAGCAGGGGCCTTTTTACATG	AsxR 5'RLM-RACE
EcOnc03.5.RACE.R	CATGAGATACTTGCATTGTC	EcOnc03 5'RLM-RACE
map.5.RACE.R	CATTGTCATTGGACTAAACAT	Map 5'RLM-RACE
DppA_sal.T7.F	GGATCCTAATACGACTCACTATAGGGAGAGGA ATGAGGGGCATTTTATGGAG	Add T7 promoter for IVT
DppA_sal.R	CAGCATCCCTGACTTCTTCAAG	Add T7 promoter for IVT
GcvB.T7.F	GGATCCTAATACGACTCACTATAGGGAGAGGA ACTTCCTGAGCCGGAACGAAAAG	Add T7 promoter for IVT
GcvB.R	AAAAAAAGCACCGCAATTAGGCGGTGC	Add T7 promoter for IVT
AgvB.T7.F	GGATCCTAATACGACTCACTATAGGGAGAGGA CGATAACACAACAATATCAG	Add T7 promoter for IVT
AgvB.R	AAAAAATGCCCGAATGGGTTCGGGC	Add T7 promoter for IVT
FnrS.T7.F	GGATCCTAATACGACTCACTATAGGGAGAGGA GCAGGTGAATGCAACGTCAAG	Add T7 promoter for IVT
FnrS.R	AAAAAGCCGACTCATCAAAGTCGGCG	Add T7 promoter for IVT
**Probe**	**Sequence**	**Target**
EcOnc01	GCAATAGCATGAGATACTGATATTGTTGTGTTG	EcOnc01
EcOnc02	GCAATTGCATGAGATACTCGTTTTAATAATCGAA	EcOnc02
EcOnc03	ACAGCATGAGATACTTGCATTGTCATTTTTATCG	EcOnc03
EcOnc04	TTGAGAAAAACAGAAATAACA CTTTTGTGGCAAAG	EcOnc04
EcOnc05	AATCCGCGATTATCCCATATACCTACTCGCTGATT	EcOnc05
EcOnc06	GTATCAGCACCTGAATCCGCGATTATCCCATATAC	EcOnc06
EcOnc07	GGTTCTTGCTGTTTGTACTTTGTCTCAGGAATTAC	EcOnc07
EcOnc08/21/42	AATAATATCCGACAGTAATCACTCTGCGCAATAGC	EcOnc08/21/42
EcOnc13	TTTTCTAACACATCTATTATCAGACCGGCAACAAC	EcOnc13
EcOnc18	TTTGGTGATATCTACGCCTGGTTCGGTCGCGTACT	EcOnc18
EcOnc22/23/24	GGGAGAGAACGATGAAGATTAACGTTATGGAGTTA	EcOnc22/23/24
EcOnc27	GGCCTTGGTATATGCCTAATCTCTGTATACTGCAT	EcOnc27
EcOnc32	ATATTCCCCGTGGAGAAATGATATGTAACACACAT	EcOnc32
EcOnc33	AAATGT CGTTATATCCAAAGCAAAACATGCAGGAC	EcOnc33
EcOnc38	AGATAAGAGTAAACAAACCAACAGCAGCAAAGACA	EcOnc38
EcOnc50	GCTGATTACTTCAGCCAAAAGGAACACCTGTATAT	EcOnc50
EcOnc53	AAGTAGAAGGTTAGCGCCTCTCTGTAAAAGGAGTC	EcOnc53
AsxR_muts	GTGGGTTCGGGCAATTGCATGAGATACTCGTTT	Targets AsxR outside S1 mutation. For use with cloned constructs in DH5alpha
EcOnc01_muts	AATGGGTTCGGGCAATAGCATGAGATACTGATAT	Targets EcOnc01 outside G1 mutation. For use with cloned constructs in Top10F'
sfGFP_probe	CATTGAACACCATAGGTCAGAGTAGTGACAAGTGT	superfolderGFP
eGFP_probe	CCATGATATAGACGTTGTGGCTGTTGTAGT	eGFP
GcvB_probe	GGTGCTACATTAATCACTATGGACAGACAGGGTAA	GcvB
FnrS_probe	CGTACGAATCAATTGTGCTATGCAGTAATTCAAA	FnrS

SUPPLEMENTARY TABLE REFERENCES

Blattner, F.R., Plunkett, G., 3rd, Bloch, C.A., Perna, N.T., Burland, V., Riley, M., Collado-Vides, J., Glasner, J.D., Rode, C.K., Mayhew, G.F., *et al.* (1997). The complete genome sequence of *Escherichia coli* K-12. Science *277*, 1453-1462.

Campellone, K.G., Robbins, D., and Leong, J.M. (2004). EspFU is a translocated EHEC effector that interacts with Tir and N-WASP and promotes Nck-independent actin assembly. Dev Cell *7*, 217-228.

Cherepanov, P.P., and Wackernagel, W. (1995). Gene disruption in Escherichia coli: TcR and KmR cassettes with the option of Flp-catalyzed excision of the antibiotic-resistance determinant. Gene *158*, 9-14.

Corcoran, C.P., Podkaminski, D., Papenfort, K., Urban, J.H., Hinton, J.C., and Vogel, J. (2012). Superfolder GFP reporters validate diverse new mRNA targets of the classic porin regulator, MicF RNA. Mol Microbiol *84*, 428-445.

Dahan, S., Knutton, S., Shaw, R.K., Crepin, V.F., Dougan, G., and Frankel, G. (2004). Transcriptome of enterohemorrhagic *Escherichia coli* O157 adhering to eukaryotic plasma membranes. Infect Immun *72*, 5452-5459.

Merlin, C., McAteer, S., and Masters, M. (2002). Tools for characterization of *Escherichia coli* genes of unknown function. J Bacteriol *184*, 4573-4581.

Sharma, C.M., Darfeuille, F., Plantinga, T.H., and Vogel, J. (2007). A small RNA regulates multiple ABC transporter mRNAs by targeting C/A-rich elements inside and upstream of ribosome-binding sites. Genes Dev *21*, 2804-2817.

Sittka, A., Pfeiffer, V., Tedin, K., and Vogel, J. (2007). The RNA chaperone Hfq is essential for the virulence of *Salmonella typhimurium*. Mol Microbiol *63*, 193-217.

Taylor, R.G., Walker, D.C., and McInnes, R.R. (1993). *E. coli* host strains significantly affect the quality of small scale plasmid DNA preparations used for sequencing. Nucleic Acids Res *21*, 1677-1678.

Urban, J.H., and Vogel, J. (2007). Translational control and target recognition by *Escherichia coli* small RNAs in vivo. Nucleic Acids Res *35*, 1018-1037.

EXTENDED EXPERIMENTAL PROCEDURES

Strain and plasmid construction

E. coli O157:H7 str. Sakai stx- is a Shiga toxin negative derivative of the sequenced isolate O157:H7 str. Sakai (Dahan et al., 2004). For genetic manipulations strains were grown in LB broth or plates supplemented with ampicillin (50μg/ml), kanamycin (50μg/ml), tetracycline (15μg/ml), or chloramphenicol (25 μg/ml) where appropriate. Bacterial strains, plasmids and oligonucleotides used are listed in Supplementary Tables 3A-C.

Hfq was tagged chromosomally in both *E. coli* O157:H7 and K12 using the pTOF series of allelic exchange vectors (Merlin et al., 2002). The *hfq* stop codon was replaced with a BamHI site by SOE PCR. 5' and 3' PCR products were generated using the primer pairs hfq.5.NotI.F & hfq.5.BamHI_link.R and hfq.3.NotI.R & hfq.3.BamHI_link.F. SOE PCR products were 'sewn' together by amplifying with hfq.5.NotI.F and hfq.3.NotI.R using 1ul of each gel purified PCR product as template. The SOE PCR product was gel extracted and cloned into pTOF25 using NotI. The HTF::*tetRA* tag was inserted into the BamHI site of pTOF25::*hfq* to create an in frame fusion between *hfq* and the HTF affinity tag. pTOF25::*hfq*-HTF::*tetRA* was used for allelic

exchange as previously described (Tree et al., 2011). The *tetRA* cassette is flanked by FRT sites and was removed using FLIP recombinase (pCP20)(Merlin et al., 2002).

For deletion of *agvB1* and *agvB2*, flanking regions were amplified from *E. coli* O157:H7 str. Sakai using primers agvB1.PstI.F & agvB.NotI.5.R, agvB1.NotI.3.F & agvB1.SalI.R, agvB2.PstI.F & agvB.NotI.5.R, and agvB2.NotI.3.F & agvB2.SalI.R. Flanking regions were similarly sewn together by SOE PCR and cloned into pTOF24. A *tetRA* cassette was inserted from pTOF1 and allelic exchange performed as above. Allelic exchanges for *agvB1* and *agvB2* were performed sequentially, removing the *tetRA* cassette using pCP20 as previously. Marker rescue was used to repair the *agvB1::tetRA* insertion. Flanking primers agvB1-comp-for & agvB1-comp-rev were used to generate the wild type PCR product that was cloned into pTOF24. After primary integration into the Δ*agvB2*::FRT Δ*agvB1*::*tetRA* strain, integrates were subjected to repeated rounds of growth at the permissive temperature for pTOF24 replication (30°C) to select against integrates. Colonies were screened for lose of tetracycline resistance and sensitive colonies were sequenced to confer reversion to the wild type *agvB1* sequence.

A three plasmid system was used to express anti-sRNA, sRNA, and mRNA-sfGFP translational fusions. The pXGSF series of plasmids described by Corcoran *et al* (2012) were used to construct translational *chuS* fusions to sfGFP under the control of the P_{tetO-1} promoter. sRNAs were cloned into the P_{tetO-1} controlled expression vector pZA21MCS (Expressys). Anti-sRNA were cloned into the P_{lacO-1} controlled expression vector pZE12*luc* as previous described for sRNAs by Urban & Vogel (2007). For pulsed expression studies, the sequence of AsxR was cloned into pBAD*myc*His A at the transcription +1 site to generate pBAD+1::AsxR. Plasmids and oligonucleotides used in this study are listed in Supplementary Tables 3B and 3C

To construct mRNA reporter fusions, reporter sequences were amplified using primers listed in Supplementary Table 3C and cloned into pXG10SF or pXG30SF using NsiI and NheI restriction enzymes (Fermentas). Positive clones were confirmed by PCR and verified by sequencing. To clone anti-sRNA into the $P_{LlacO-1}$ expression vector pZE12*luc*, anti-sRNA sequences were incorporated into the 5' end of the forward PCR primer (Supplementary Table 3C). pZE12 was amplified using ZE12.5P.R (5' end is at the transcriptional +1 site) and the anti-sRNA primer, gel purified and self-ligated. Transformants were screened by PCR and sequenced to confirm insertion of the anti-sRNA. For construction of sRNA expression constructs, pZA21MCS was amplified using ZA21MCS.5P.R and ZA21MCS.HindIII.F to generate a linear backbone for cloning. sRNAs were amplified using primers listed in Supp Table 4C. ZA21MCS and sRNA PCR products were digested with HindIII, ligated, and transformed. Transformants were screened by PCR and verified by sequencing. To construct pZA21::GcvB, ligations were transformed into Top10F' to repress transcription of GcvB during construction. Point mutations were introduced into appropriate vectors by PCR amplification of

constructs using mutagenic primers listed in Supplementary Table 3C and were re-circularised by self-ligation.

For pulsed expression studies, AsxR was cloned under P_{ara} control by amplifying pBAD*myc*His A using BAD+1.5P.R and BAD+1.AsxR.F (Supplementary Table 3C). The PCR product was gel purified, self-ligated and transformed into DH5α to generate pAsxR. Transformants were screened by PCR and verified by sequencing. The control plasmid pBAD+1 was constructed similarly by self-ligation of the PCR product generated from pBAD+1.5P.R and pBAD+1.XbaI.F. Expression of AsxR after 10min of arabinose induction (0.2%) was confirmed by northern blot.

Functional assay for Hfq-HTF activity

We used the previously described OmpF-MicF mRNA-sRNA pair to assess the function of our Hfq-HTF fusion protein (Corcoran et al. 2012). Translational repression of OmpF-GFP by MicF requires Hfq, and this activity was used to verify that HTF tagged Hfq was functional. Overexpression of MicF repressed 82% of OmpF translation in the presence of wild type Hfq, and 16% in an Hfq delete background (Supplementary Figure S1). Translation of OmpF showed derepression in the Hfq-HTF strain (4.2 fold increase relative to wild type) indicating that the HTF tag reduces activity of OmpF (by reducing affinity or stability), however expression of MicF repressed OmpF translation by 75% demonstrating that Hfq-HTF is functional and mediates riboregulation.

UV crosslinking and analysis of cDNA (CRAC)

Hfq CRAC was performed essentially as described by Granneman et al. (2011). Control and tagged strains were cultured in 2L of MEM-HEPES media (Sigma, M72781) 250 nM $Fe(NO_3)_2$ and 0.1% glucose, or LB media to a final OD_{600} of 0.8 before UV crosslinking with 1800mJ of UV-C. Harvested cells were divided into 1g aliquots that were subsequently used for single purifications. Cell pellets were mechanically disrupted in 1V Lysis Buffer (50mM Tris.HCl (pH 7.8), 1.5mM $MgCl_2$, 150mM NaCl, 0.1% Nonidet P-40, and 5mM β-mercaptoethanol, 1 tablet 'cOmplete' EDTA free protease inhibitor (Roche)/50ml) and 3V zirconia beads (Thistle Scientific). Cell lysates were cleared by centrifugation (20 min at 4000 *g* followed by 20 min at 14000 *g* for the cleared supernatant) and incubated overnight at 4°C with 200μL of anti-FLAG M2 affinity gel (Sigma, A2220). Hfq bound anti-FLAG resin was washed twice with TNM1000 buffer (50mM Tris.HCl pH7.8, 1M NaCl, 0.1% NP-40, 5mM β-mercaptoethanol) and twice in TMN150 (50mM Tris.HCl pH7.8, 150mM NaCl, 0.1% NP-40, 5mM β-mercaptoethanol), resuspended in 500μl of TNM150 and incubated with 20-30U of TEV protease for 2hrs at 18°C . Hfq-RNA complexes were eluted by centrifugation. To "trim" RNA crosslinked to Hfq, complexes were digested with 0.085 U/ml of RNaceIT (Stratagene) for 5 min at 37°C and

inactivated in 6M-guanidine hydrochloride. Eluates were adjusted to 300mM NaCl and 10mM imidazole and added to pre-washed Ni-NTA resin (Qiagen). The Ni-NTA bound complexes were washed twice with 500μl wash buffer I (6M Guanidine-HCl, 50mM Tris-HCl pH 7.8, 300 mM NaCl, 0.1% NP-40, and 5mM β-mercaptoethanol) and three times with 1xPNK buffer (50mM Tris-HCl pH 7.8, 10mM $MgCl_2$, 0.5% NP-40, and 5mM β-mercaptoethanol). The subsequent phosphatase, linker ligation and phosphorylation reactions were performed in 80 μl reaction volumes on column. 3' ends were dephosphorylated by incubating for 45min at 37°C with thermosensitive alkaline phophatase (TSAP, Promega) and RNasin (Promega) in PNK reaction buffer (50mM Tris-HCl pH 7.8, 10mM $MgCl_2$, and 10mM β-mercaptoethanol). The resin was washed once in 400μl wash buffer I and three times in 400μl 1xPNK buffer. miRCat-33 (IDT) 3' linkers were added by incubating at 25°C for 6hrs in PNK reaction buffer containing 10μM miRCat-33 linker (IDT), RNasin, and T4 RNA ligase I (NEB). Ligation reactions were washed once with wash buffer I and three times with 1xPNK buffer. The 5' end of bound RNAs radiolabelled by phosphorylation with T4 PNK (Sigma) and ^{32}P-γATP in PNK reaction buffer for 40 min at 37°C, after which 100mM of cold ATP was added to complete 5' end phosphorylation. The resin was again washed once with wash buffer I and three times with 1xPNK buffer. The 5' linker was ligated by incubation with 10mM ATP, RNasin, L5 linker (Supplementary Table 3C), and T4 RNA ligase I in PNK reaction buffer at 16°C overnight. The ligation reaction was washed three times with wash buffer II (50mM Tris-HCl pH 7.8, 50mM NaCl, 10mM imidazole, 0.1% NP-40, 5mM β-mercaptoethanol). Hfq-RNA complexes were eluted by incubation with 2x200μl of wash buffer II supplemented with 150mM imidazole. Complexes were precipitated with 100 μl of TCA and washed with ice cold acetone. Protein-RNA pellets were resuspended in 1xNuPAGE loading buffer and separated using a 4-12% NuPAGE gradient polyacrylamide gel (Invitrogen). Complexes were transferred to nitrocellulose membranes and imaged by autoradiography. Smears of Hfq-RNA were cut from the nitrocellulose membrane just above the molecular weight for Hfq alone Supplementary Figure S1) and protein digested from the membrane by incubation with 100μg of Protease K in wash buffer II containing 1% SDS and 5mM EDTA for 2hrs at 55°C. 50 μl of 3M sodium acetate pH 5.2 was added to Proteinase K supernatant and RNA extracted using phenol:chloroform:isoamylalcohol and ethanol precipitation. The RNA pellet was resuspended in 13μl of RT buffer I (miRCat RT oligo and 5mM dNTPs) and reverse transcribed using Superscript III as per manufacturers instructions. cDNA was amplified using Takara LA Taq, P5 and PE_miRCat PCR primers (Supplementary Table 3C), and 2 μl of cDNA. cDNAs were amplified for 20-24 cycles to minimize bias in amplicons. 3-10 PCR reactions were pooled and ethanol precipitated. PCR products were separated on a 3% metaphor agarose gel and smeared amplicons above primer dimers indicated in control samples were gel extracted (Supplementary Figure S1) using a MinElute

gel extraction Kit (Qiagen). To test the quality of the CRAC library, 2μl of PCR product was cloned using a TOPO TA cloning kit for sequencing and 10-96 clones were sequenced by Sanger sequencing. High throughput sequencing was performed using Illumina's Solexa GAxII and HiSeq2000 platforms. Sequencing data is available at GEO (ncbi.nlm.nih.gov/geo/) under the accession number GSE46118 within the superseries GSE46120.

In silico analysis of Hfq crosslinked sequences

Analysis of reads bound by Hfq was performed using the pyCRAC software package {Webb, 2014 #298}, BEDTools (Quinlan and Hall, 2010), awk and Perl scripts previously described (Helwak et al., 2013; Wlotzka et al., 2010), and custom Python scripts. Data were plotted using R and Excel. Total genomic data were plotted using circos (Krzywinski et al., 2009) and the Integrated Genome Browser (Nicol et al., 2009).

Motif analysis. Three to six nucleotide k-mers that were enriched in Hfq bound reads (transcriptome-wide) were identified using the pyMotif module of the pyCRAC package {Webb, 2014 #298}. The top 10 k-mers in each dataset were used to generate a Logo using GLAM2 from the MEME suite of tools (Frith et al., 2008). Frequency plots of Hfq bound reads, read clusters, or deletions at genomic features (including CDS, motifs, and start codons) were constructed using pyBinCollector (pyCRAC package), and custom Perl scripts. Nucleotide frequency and structure scores around maximal crosslinking sites within sRNA were calculated using custom Python scripts. The secondary structure of 21 sRNAs reported to bind Hfq (ccb.bmi.ac.cn/srnatarbase/index.php) were predicted using hybrid-ss-min (UNAfold package) (Markham and Zuker, 2008). Structure scores around maximal crosslinking sites were calculated from the dot-bracket structure assigning +1 paired and -1 for unpaired. Statistical significance was calculated from data with randomly assigned crosslinking sites using a Monte-Carlo approach whereby the random frequency of nucleotide occurrence or structure score was calculate from 1000 iterations of randomly assigned maximal crosslinking sites using $p=r+1/n+1$, where r = iterations and n = # of iterations greater than the test value, to calculate the probability (p) of randomly obtaining a nucleotide enrichment or structure score. The false discovery rate was calculated as $q=p/r$ where r = number of tests.

Non-genomically encoded A-tails. Non-genomically encoded oligo(A) tails were identified as previously described (Wlotzka et al., 2010) excepting only tails composed completely of (A) were retained.

Experimentally verified mRNA and sRNA seed sequences. The coordinates for experimentally verified sRNA and mRNA seed sequences were extracted from sRNATarbase (Cao et al., 2010) for MG1655. K12 coordinates were converted to O157 str. Sakai coordinates using blastn and custom Python scripts. Recent studies have defined multiple mRNA targets for GcvB (Sharma et al., 2011), Spot42 (Beisel et al., 2012), and MicF (Corcoran et al., 2012)

and these were similarly converted to Sakai coordinates. Where Salmonella sequences were not identical to O157 sequences, duplex formation between the sRNA and mRNA was confirmed using IntaRNA (Busch et al., 2008). A total of 46 sRNA:mRNA seed pairs were used for data analysis. Where necessary overlapping sRNA seed sequences were condensed into sRNA seed regions by combining overlapping seed sequences into a single seed region using mergeBed, this yielded 21 sRNA seed regions. sRNA that were confirmed to bind Hfq were similarly extracted from sRNATarbase and used to identify enriched features at Hfq binding sites.

Identification of sRNAs. Peaks of Hfq binding were identified using custom awk scripts previously described (Helwak et al., 2013) excepting an arbitrary cluster threshold of 1000 (area under curve) was used and peak width set to +/- 20 nt of the peak maxima. Peaks present in at least 3/5 datasets were identified using mergeBed (Quinlan and Hall, 2010) and retained for further analysis. To identify sRNAs bound by Hfq: peaks within or <100nt from the sense strand of a transcribed genomic feature were excluded using intersectBed. Similarly, tRNA encoded within S-loops were also removed from the dataset using positions defined by GtRNAdb (gtrnadb.ucsc.edu) (Chan and Lowe, 2009). Rho-independent terminators were identified using RNAmotif (Macke et al., 2001) and the terminator descriptor defined by Lesnik et al. (2001). Peaks were considered to be associated with a terminator if a predicted terminator was within <200nt of the 3' end. Peaks >100nt from a genomic feature, that were not predicted to be tRNA, were considered potential sRNA with or without a predicted terminator.

In silico prediction of sRNA and anti-sRNA targets. IntaRNA software (Busch et al., 2008) was used to predict interactions between anti-sRNA, sRNA, and mRNAs.

5' RLM-RACE using tobacco acid pyrophophatase (TAP)

5' RLM-RACE was used to map the 5' end of transcripts and distinguish primary triphosphate from mono phosphorylated 5' ends. Total RNA was isolated using Trizol reagent as per manufactures instructions (Invitrogen). Six micrograms of total RNA was incubated in 1X TAP buffer and +/- 1U of TAP (Epicentre) for 1hr at 37°C in a total volume of 10μL. 2μL of TAP treated RNA was transferred into an 8μL ligation reaction containing 200pmol of L5 Solexa linker, 1X RNA ligase buffer and 20U of T4 RNA ligase and incubated at 16°C overnight. Ligation reactions were precipitated in 500μL ice cold ethanol and 20μL 3M NaAc pH5.2 and incubated at -80°C for 30min, centrifuged at 14000 *g*, aspirated, washed with 500μL of 70% ethanol, aspirated and air dried. Precipitated ligation reactions were resuspended in 10μL of [10μM random hexamers, 1mM dNTPs] and reverse transcribed using Superscript III as per manufactures instructions. 2μL of cDNA was PCR amplified using a touchdown PCR protocol (Don et al., 1991) with P5 primer and a gene specific primer (Supplementary Table 3C). PCR products were separated on a 3% Metaphor agarose TBE gel. Bands were excised and purified

using a Qiagen minElute gel extraction kit and cloned using a TOPO TA Cloning Kit for Sequencing (Invitrogen). 10-20 clones were sequenced for each purified band.

Northern blot analysis

Total RNA was extracted by GTC-Phenol extraction. Five micrograms of total RNA was separated on either a 1.5% agarose BPTE-glyoxal gel or, for short RNA fragments, an 8% polyacrylamide TBE-Urea gel and transferred to a nylon membrane and UV crosslinked. Membranes were pre-hybridised in 5ml of UltraHyb Oligo Hyb (Ambion) and probed with 10pmol of ^{32}P end labeled 35mer DNA oligo (Supplementary Table 3C). Membranes were washed 3x in 2x SSPE + 0.1% SDS for 10min, and visualized using a Fuji BAS-MP 2040 Phosphor screen and FujiFilm FLA-5100 Scanner.

Microarray analysis of AsxR

Cultures of *E. coli* O157:H7 str. TUV93-0 containing pAsxR or pBAD+1 were grown for 16hr in LB and diluted 1/100 into MEM-HEPES supplemented with 0.1% glucose and 250nM $FeCl_3$. Cells were induced with 0.2% L-arabinose at OD_{600} 0.8 for 10min and 10ml of cells harvested and stabilised in an equal volume of RNAProtect. Total RNA was extracted using a Qiagen RNeasy mini Kit and labeled using the Superscript Plus indirect labeling system (Invitrogen) as per manufactures instructions. Labelled cDNA was hybridized to custom oligonucleotide arrays (UBEC Array 3, University of Birmingham, UK) using a Maui hybridisation machine (Maui). Microarrays were scanned using an Axon 4100A autoloader scanner and data analysed using Genespring 7.3.1 software. Array data is available at GEO (ncbi.nlm.nih.gov/geo/) under the accession number GSE46113 within the superseries GSE46120 .

Fluorescent reporters of translation

The three plasmid system for expression of GFP and sfGFP translational fusions in anti-sRNA and sRNA expressing backgrounds were performed in *E. coli* DH5α, for *chu* operon and fragment fusions, and *E. coli* Top10F' for $DppA_{Sal}$ fusions as this background permitted controlled expression of GcvB. Cultures containing GFP and superfolderGFP fusions were cultured overnight in 5ml of LB and diluted 1/100 in M9 supplemented with 100 μg/ml of L-leucine for Top10F' cultures. M9 cultures were grown overnight at 37°C and fluorescence measured using an Infinite M200 microplate reader (Tecan). Fluorescence measurements were normalized to OD_{600}. For constructs expressed from Top10F' backgrounds, 0.25 ng/μl of anhydrotetracycline and 1mM IPTG was added to induce transcription from P_{tetO-1} and P_{lacO-1} respectively. FACS analysis was performed using a FacsCaliber flow cytometer (Becton Dickenson). For analysis of the $DppA_{EHEC}$ translational fusion in *E. coli* O157:H7 str. Sakai and derivatives, five transformants were inoculated into 5ml of LB for overnight growth. Cultures

were diluted 1/100 into MEM-HEPES supplemented with 250 nM $Fe(NO_3)_2$ and 0.1% glucose and grown to OD_{600} ~0.6. Fluorescence was measured using a FLUOstar Optima fluorescence plate reader (BMG Labtech, Germany) and normalized to OD_{600}.

Competitive index experiments. Overnight LB cultures of *E. coli* O157 str. Sakai or derivatives were prepared and adjusted to OD_{600} 0.7. To compete and the WT and the isogenic Δ*agvB* Δ*agvB2* mutant, 10μl of each was added to 5ml of LB, 5ml of MEM-HEPES (supplemented with supplemented with 250 nM $Fe(NO_3)_2$ and 0.1% glucose), or 1ml of 10% bovine terminal rectal mucus diluted in sterile water. The terminal rectal mucus was collected from calves subject to post-mortems carried out at the Moredun Research Institute. Six batches of mucus were prepared, with a single batch made up of mucus collected from five different animals. Cultures were grown overnight with shaking at 37°C and 10μl transferred into fresh media of the same for overnight growth. Cultures were serially diluted and plated on LB plates containing kanamycin (both strains) or kanamycin + tetracycline (Δ*agvB1* Δ*agvB2* mutant only). The isogenic Δ*agvB1* Δ*agvB2* mutant and its *agvB1* chromosomal complement were competed in diluted bovine terminal rectal mucus only, again with selection for both strains on kanamycin and colony testing for tetracycline resistance (Δ*agvB1* Δ*agvB2* mutant only). The competitive index for culture was calculated by dividing the ratio of the competing strains in the output by their ratio in the input.

EMSA analysis of AgvB, GcvB, and DppA mRNA interactions with Hfq
Hfq was purified from *E. coli* str. MG1655 *hfq*-HTF::*tetRA* using the hexaHis tag. Two litres of LB culture were harvested by centrifugation and resuspended in 30ml of Buffer A (500mM NaCl, 50mM Tris-HCl pH7.5, 0.1% Triton, 10% glycerol, 20mM imidazole) with one tablet of 'cOmplete' protease inhibitor (Roche). Cells were incubated on ice for 30min with 3mg/ml of lysozyme (Sigma), and sonicated. Cell debris was removed by centrifuging at 20000*g* for 20 min. Supernatants were filtered through a 0.22 μm filter and incubated at 18°C for 1 hrs with 20μl of GST-TEV protease (5U/μl). Hfq-His was purified using an AKTA FLPC (Edinburgh Protein Purification Facility) and His HiTrap column with a elution gradient of increasing imidazole from 20mM to 500mM.

AgvB, GcvB, and a fragment of DppA mRNA encompassing the 5' UTR + 5nt of CDS was *in vitro* transcribed using T7 RNA polymerase (100ng PCR product, 0.5mM rNTPs, 5mM DTT, 100ng/ml BSA, 1X transcription buffer, 20U T7 polymerase). IVT reactions were depleted of DNA template by incubation with DNase I for 15min at 37°C and RNA purified by phenol:chloroform extraction and ethanol precipitation. RNAs were separated on an 8% polyacrylamide TBE-6M Urea gels and full length products extracted from gel slices, crushed, and soaked overnight at 4°C in 500 μl of Elution Buffer (10mM Magnesium acetate, 0.5M

Ammonium acetate, 1mM EDTA). Gel fragments were removed from the supernatant by centrifugation and RNAs recovered by phenol:chlorform extraction and ethanol precipitation. 100 – 160pmol of RNA was dephosphorylated using calf intestinal phosphatase (Promega), and again phenol chloroform extracted and ethanol precipitated. RNAs were end labelled using polynucleotide kinase and ATP-γ^{32}P. Labelled, full length transcripts were purified from an 8% polyacrylamide TBE-6M Urea gel as previously.

For analysis of Hfq binding to single RNAs; ~40pmol of labelled RNA was incubated with increasing Hfq in 1X Binding Buffer (10mM Tris-HCl [pH 7.4], 0.1mM EDTA, 10mM NH_4Cl, 10mM NaCl, 10mM KCl), 1mg/ml tRNA, and 4% glycerol + bromophenol blue). Reactions were incubated at room temperature for 5 min and separated on a native 5% polyacrylamide 0.5X TBE gel. For analysis of complexes formed in the presence of multiple RNAs and to allow stable duplex formation, Binding buffer was replaced with Duplex Buffer (40mM Tris-Acetate, 0.5mM Magnesium Acetate, 100mM NaCl). 0.5X TBE was also replaced with 1X Duplex Buffer in both native 5% polyacrylamide gels and running buffer. Approximately 40pmol of ^{32}P labelled RNA was incubated either 500nM (AgvB and GcvB) or 50nM Hfq (DppA) in the presence of a 50 fold excess of unlabelled RNA. Reactions were incubated at room temperature for 15min and separated on polyacrylamide gels. Gels were run at 7.5 V/cm to limit heating of the gel, dried onto Whatmann filter paper and visualized.

For western analysis of Hfq in EMSA gels, dried gels were assembled into a BioRad Trans-Blot Cell and submerged in 1X NuPAGE MOPS SDS running buffer (Invitrogen). The assembled apparatus was incubated overnight at 4°C to allow rehydration of the EMSA gel before transfer. His tagged Hfq was detected using monoclonal anti-His antibody (Sigma H1029).

For control assays to verify that Hfq is required for efficient duplex formation between AgvB and GcvB, binding reactions were carried out in Duplex buffer (as above) excepting 100nM of unlabelled RNAs were used in the reaction. Binding reactions were separated on 6% native duplex buffered polyacrylamide gels as previously and AgvB and GcvB detected by northern blot with probes EcOnc01 and GcvB_probe (Supplementary Table 3C). For control assays for Hfq migration with and without purified AgvB, binding reactions were run on a 6% native duplex buffered polyacrylamide gel and transferred to nitrocellulose using a Mini Trans-Blot Cell (BioRad). His tagged Hfq was detected using a monoclonal anti-polyHistidine-peroxidase conjugated antibody (Sigma).

SUPPLEMENTARY REFERENCES

Beisel, C.L., Updegrove, T.B., Janson, B.J., and Storz, G. (2012). Multiple factors dictate target selection by Hfq-binding small RNAs. EMBO J *31*, 1961-1974.

Busch, A., Richter, A.S., and Backofen, R. (2008). IntaRNA: efficient prediction of bacterial sRNA targets incorporating target site accessibility and seed regions. Bioinformatics *24*, 2849-2856.

Cao, Y., Wu, J., Liu, Q., Zhao, Y., Ying, X., Cha, L., Wang, L., and Li, W. (2010). sRNATarBase: a comprehensive database of bacterial sRNA targets verified by experiments. RNA *16*, 2051-2057.

Chan, P.P., and Lowe, T.M. (2009). GtRNAdb: a database of transfer RNA genes detected in genomic sequence. Nucleic Acids Res *37*, D93-97.

Corcoran, C.P., Podkaminski, D., Papenfort, K., Urban, J.H., Hinton, J.C., and Vogel, J. (2012). Superfolder GFP reporters validate diverse new mRNA targets of the classic porin regulator, MicF RNA. Mol Microbiol *84*, 428-445.

Dahan, S., Knutton, S., Shaw, R.K., Crepin, V.F., Dougan, G., and Frankel, G. (2004). Transcriptome of enterohemorrhagic *Escherichia coli* O157 adhering to eukaryotic plasma membranes. Infect Immun *72*, 5452-5459.

Don, R.H., Cox, P.T., Wainwright, B.J., Baker, K., and Mattick, J.S. (1991). 'Touchdown' PCR to circumvent spurious priming during gene amplification. Nucleic Acids Res *19*, 4008.

Frith, M.C., Saunders, N.F., Kobe, B., and Bailey, T.L. (2008). Discovering sequence motifs with arbitrary insertions and deletions. PLoS Comput Biol *4*, e1000071.

Granneman, S., Petfalski, E., and Tollervey, D. (2011). A cluster of ribosome synthesis factors regulate pre-rRNA folding and 5.8S rRNA maturation by the Rat1 exonuclease. EMBO J *30*, 4006-4019.

Helwak, A., Kudla, G., Dudnakova, T., and Tollervey, D. (2013). Mapping the human miRNA interactome by CLASH reveals frequent noncanonical binding. Cell *153*, 654 - 665.

Krzywinski, M., Schein, J., Birol, I., Connors, J., Gascoyne, R., Horsman, D., Jones, S.J., and Marra, M.A. (2009). Circos: an information aesthetic for comparative genomics. Genome Res *19*, 1639-1645.

Lesnik, E.A., Sampath, R., Levene, H.B., Henderson, T.J., McNeil, J.A., and Ecker, D.J. (2001). Prediction of Rho-independent transcriptional terminators in *Escherichia coli*. Nucleic Acids Res *29*, 3583-3594.

Macke, T.J., Ecker, D.J., Gutell, R.R., Gautheret, D., Case, D.A., and Sampath, R. (2001). RNAMotif, an RNA secondary structure definition and search algorithm. Nucleic Acids Res *29*, 4724-4735.

Markham, N.R., and Zuker, M. (2008). UNAFold: software for nucleic acid folding and hybridization. Methods Mol Biol *453*, 3-31.

Merlin, C., McAteer, S., and Masters, M. (2002). Tools for characterization of *Escherichia coli* genes of unknown function. J Bacteriol *184*, 4573-4581.

Nicol, J.W., Helt, G.A., Blanchard, S.G., Jr., Raja, A., and Loraine, A.E. (2009). The Integrated Genome Browser: free software for distribution and exploration of genome-scale datasets. Bioinformatics *25*, 2730-2731.

Quinlan, A.R., and Hall, I.M. (2010). BEDTools: a flexible suite of utilities for comparing genomic features. Bioinformatics *26*, 841-842.

Sharma, C.M., Papenfort, K., Pernitzsch, S.R., Mollenkopf, H.J., Hinton, J.C., and Vogel, J. (2011). Pervasive post-transcriptional control of genes involved in amino acid metabolism by the Hfq-dependent GcvB small RNA. Mol Microbiol *81*, 1144-1165.

Tree, J.J., Roe, A.J., Flockhart, A., McAteer, S.P., Xu, X., Shaw, D., Mahajan, A., Beatson, S.A., Best, A., Lotz, S.*, et al.* (2011). Transcriptional regulators of the GAD acid stress island are carried by effector protein-encoding prophages and indirectly control type III secretion in enterohemorrhagic *Escherichia coli* O157:H7. Mol Microbiol *80*, 1349-1365.

Urban, J.H., and Vogel, J. (2007). Translational control and target recognition by *Escherichia coli* small RNAs in vivo. Nucleic Acids Res *35*, 1018-1037

Wlotzka, W., Kudla, G., Granneman, S., and Tollervey, D. (2010). The nuclear RNA polymerase II surveillance system targets polymerase III transcripts. EMBO J *30*, 1790-1803.
